# Marine Indole Alkaloids—Isolation, Structure and Bioactivities

**DOI:** 10.3390/md19120658

**Published:** 2021-11-24

**Authors:** Yong Hu, Siling Chen, Fang Yang, Shuai Dong

**Affiliations:** Key Laboratory of Tropical Biological Resources of Ministry of Education, School of Pharmaceutical Sciences, Hainan University, Haikou 570228, China; hy15260001800@163.com (Y.H.); siling0420@163.com (S.C.); yf200110237@163.com (F.Y.)

**Keywords:** marine alkaloids, indoles, natural products, cytotoxicity, antibacterial, bioactivities

## Abstract

Indole alkaloids are heterocyclic natural products with extensive pharmacological activities. As an important source of lead compounds, many clinical drugs have been derived from natural indole compounds. Marine indole alkaloids, from unique marine environments with high pressure, high salt and low temperature, exhibit structural diversity with various bioactivities, which attracts the attention of drug researchers. This article is a continuation of the previous two comprehensive reviews and covers the literature on marine indole alkaloids published from 2015 to 2021, with 472 new or structure-revised compounds categorized by sources into marine microorganisms, invertebrates, and plant-derived. The structures and bioactivities demonstrated in this article will benefit the synthesis and pharmacological activity study for marine indole alkaloids on their way to clinical drugs.

## 1. Introduction

Marine natural products have incomparable skeleton diversity and novelty relative to terrestrial source ones. They often exhibit superexcellent physiological activities and occupy an important position in today’s pharmaceutical industry as a continuously rich source of potential drugs [1,2,3,4,5]. The diversity of their structure enables them to have a broader range of pharmacological activities and action mechanisms, such as neuroprotection, analgesia, smoking cessation, antibacterial, antiviral, antitumor, antihypotension, and antihyperlipidemia [6].

The indole nucleus is one of the most crucial ring systems in nature. It has been termed a “privileged structure” in respect of pharmaceutical development. Viibryd (vilazodone, neurological disorders), decapeptyl (triptorelin, hormonal disorders), symdeko (tezacaftor and lvacaftor, genetic disorders), cialis (tadalafil, sexual health), cubicin (daptomycin, anti-bacterial), zepatier (elbasvir and grazoprevir, infectious diseases), tagrisso (osimertinib, oncology), sutent (sunitinib, oncology), zoladex (goserelin, oncology), alecensa (alectinib, oncology) and lupron (leuprolide, oncology) are all indole-containing top 200 small molecule pharmaceuticals by retail sales in 2018, which were summarized by Njarðarson Group (The University of Arizona, https://njardarson.lab.arizona.edu, 30 October 2021). Due to the high market occupancy and diverse physiological activities, indole alkaloids are now a research hotspot for pharmacologists. In recent years, pharmacological activities of indole alkaloids have been reviewed, including indole alkaloids with anti-diabetic activity [7], anti-malarial potential [8], anti-depression and anti-anxiety activity [9], antitumor and anti-drug-resistant cancer activity [10,11], and immune-regulatory activity [12]. This review focuses on marine indole alkaloids discovered since 2015, when the last comprehensive review, covering the time from 2003 to 2015, was reported by Netz and Opatz [13]. In this review, the newly isolated and structure-revised indole alkaloids from 2005 to 2021, 472 in total, are reported by the classification of sources. All the chemical structures are drawn in this review, and the bioactivities are discussed. The general information of the cell lines mentioned in this review are listed in Table S1. The sources and bioactivities of all the reviewed marine indole alkaloids are summarized in Tables S2 and S3. The structures of these marine indole alkaloids were elucidated by various spectroscopic techniques. High-resolution mass spectrometer (HRMS) and 1D/2D nuclear magnetic resonance (1D/2D NMR) are the primary techniques for structure determination. Ultraviolet (UV) and infrared (IR) data are also used as auxiliary proofs. For compounds with chiral centers, the absolute configurations could be determined by specific rotation, electronic circular dichroism (ECD), X-ray single-crystal diffraction and Marfey’s method, etc. During the discovery of natural products, structures were mistaken especially for absolute configurations, which happened occasionally. Chemical total synthesis of the natural product and comparing the NMR spectroscopy between the synthetic product and the natural product is another precise but complex and expensive method for structure determination and revision. 

## 2. Marine Microorganisms

### 2.1. Marine-Sourced Bacteria 

Marine-sourced bacteria are one of the richest producers of bioactive natural products. There are 64 new indole metabolites isolated from marine-sourced bacteria, including 38 from sediment-sourced bacteria and 12 from sponge-sourced bacteria. If classified by the source of bacterial species, most of the indole alkaloids are found from actinomycetes.

#### 2.1.1. Sediment-Sourced Bacteria

**Figure 1:**
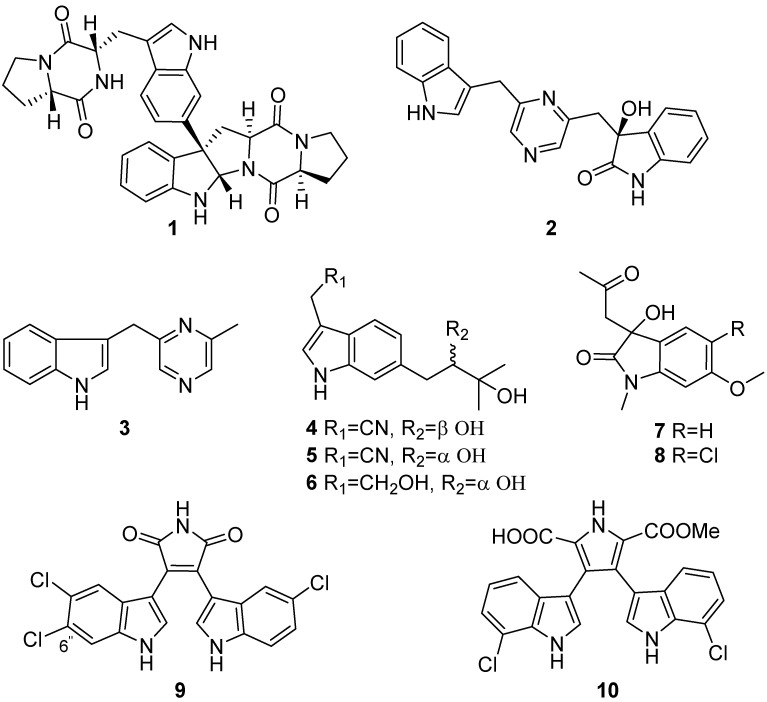
Chemical structures of **1**–**10**.

Isonaseseazine B (**1**), an antimicrobial diketopiperazine dimer, was isolated from *Streptomyces* sp. SMA-1 by bioassay-guided separation (Figure 1). *Streptomyces* sp. SMA-1 was one of the 613 actinobacterial strains isolated from the sediments collected from the Yellow Sea, China [14]. Indolepyrazines A (**2**) and B (**3**) were isolated from *Acinetobacter* sp. ZZ1275, and they showed antimicrobial activities against methicillin-resistant *Staphylococcus aureus* (MRSA), *Escherichia coli* (*E. coli*), and *Candida albicans* with minimum inhibitory concentration (MIC) values of 12 μg/mL, 8–10 μg/mL, and 12–14 μg/mL, respectively. Indolepyrazine A (**2**) is the first indole-pyrazine-oxindole alkaloid, and both **2** and **3** are the first reported natural products isolated from marine-derived *Acinetobacter* species [15]. Streptoprenylindoles A–C (**4**–**6**) were acquired from *Streptomyces* sp. ZZ820. Streptoprenylindoles A and B were enantiomers that were separated by the preparation of Mosher’s method. No inhibiting activities of the streptoprenylindoles were reported for the tested MRSA and *E. coli* [16]. 3-hydroxy-N-methyl-2-oxindole (**7**–**8**) were obtained from marine *Salinispora arenicola* strain from sediments of Brazil, and they showed no antibacterial activity against Gram-positive (*Enterococcus faecalis* and *Staphylococcus aureus*) and Gram-negative (*E. coli*) bacteria strains [17]. Two new chlorinated bisindole alkaloids, dionemycin (**9**) and 6-OMe-7′,7″-dichorochromopyrrolic acid (**10**) were isolated from the deep-sea derived *Streptomyces* sp. SCSIO 11791. In vitro antibacterial and cytotoxic assays revealed that compound **9** shows anti-staphylococcal activity with a MIC range of 1–2 μg/mL against six clinic strains of MRSA isolated from human and pig. The cytotoxicity of the trichloro-bisindole **9** was evaluated on human cancer cell lines NCI-H460, MDA-MB-231, HCT-116, HepG-2, and noncancerous MCF10A with IC_50_ values ranging from 3.1 to 11.2 μM. Structure–activity relationship analysis of compounds **9**, **10**, and seven known analogs showed C-6″ chlorine as an essential pharmacophore in their cytotoxic activities [18]. 

**Figure 2:**
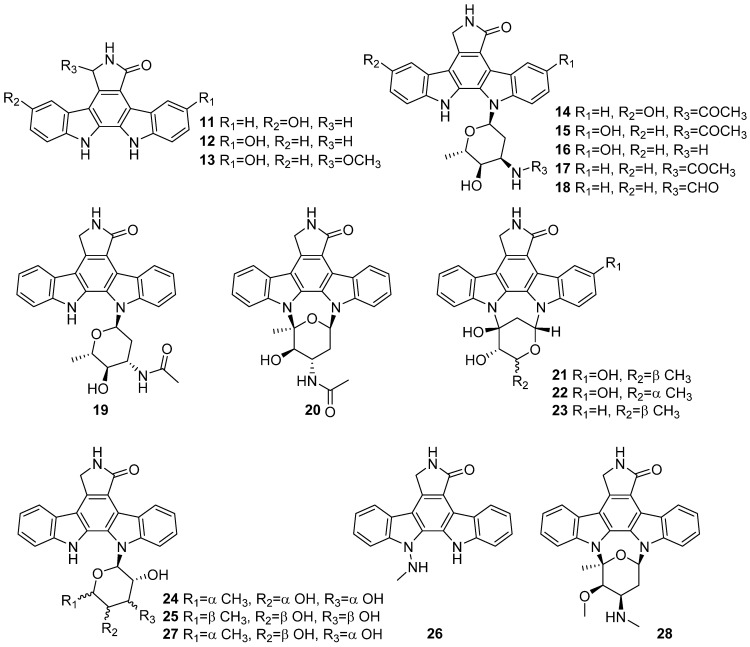
Chemical structures of **11**–**28**.

A total of 18 new indolocarbazole alkaloids (**11**–**28**) isolated from *Streptomyces* sp. DT-A61, A65, A68, A22, OUCMDZ-3118 and *bingchenggensis* ULS14 were reported in a roll during 2018 and 2019 (Figure 2). Compounds **11**–**25** were evaluated for cytotoxic activity against PC3 cell line. Compound **20** showed the strongest cytotoxic activity against PC3 with an IC_50_ value of 0.15 μM, and the other indolocarbazoles exhibited moderate activities against the PC3 (IC_50_ = 0.8–41.3 μM). Compounds **11**–**25** were also tested for various enzyme inhibition activities of protein kinase C and bruton tyrosine kinase. Compound **12** displayed significant and selective inhibition against ROCK2 and the other indolocarbazole also showed moderate inhibition activities to different kinases. Compound **26** showed moderate activity with IC_50_ values of 0.91–1.84 μM for the tested protein kinases enzyme inhibition activities. Compound **27** was moderately effective against the A549 and MCF-7 cell lines with IC_50_ values of 1.2–1.6 μM. The IC_50_ of **28** against the HeLa cell line was 0.075 μg/mL [19,20,21,22,23,24]. 

**Figure 3:**
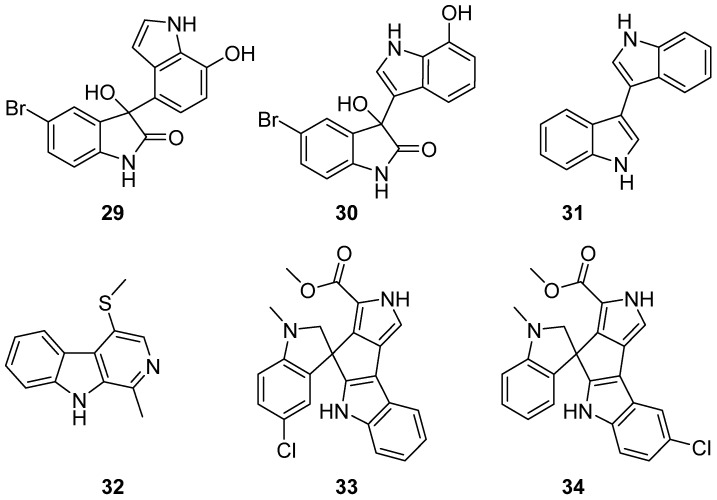
Chemical structures of **29**–**34**.

Two new brominated bis-indole metabolites, 5-bromometagenediindole B (**29**), and 5-bromometagenediindole C (**30**) were separated under the guidance of LC-MS from the 25D7 clone derived *E*. *coli* fermentation broth, in which 5-bromoindole was added. 5-Bromometagenediindole B (**29**) demonstrated moderately cytotoxic activity against MCF-7, B16, CNE-2, BEL-7402, and HT-1080 tumor cell lines in vitro (Figure 3) [25]. 3,3′-bis-indole (**31**) were isolated from sediment-derived actinomycete *Nocardiopsis* sp. G057 as a natural product for the first time. Compound **31** exhibited antimicrobial activity against several strains of bacteria, and the yeast *Candida albicans* with values of MIC ranging from 64 to 256 µg/mL. Cytotoxic evaluation of compound **31** against four cancer cell lines (KB, LU-1, HepG-2, and MCF-7) indicated that **31** produced a weak inhibition against KB and LU cell lines (IC_50_ = 12.5 and 25.6 µg/mL) [26]. 1-methyl-4-methylthio-β-carboline (**32**) was tracked by the GNPS MS^2^ fragmentation pattern analysis tool and separated by a scale-up liquid culture of *Achromobacter spanius*. No bioactivity was reported [27]. Spiroindimicins E (**33**) and F (**34**) were identified by combined genomics-metabolomics profiling of marine *Streptomyces* sp. MP131-18, and demonstrated the potential of actinomycetes in combinatorial biosynthesis of secondary metabolites [28].

**Figure 4:**
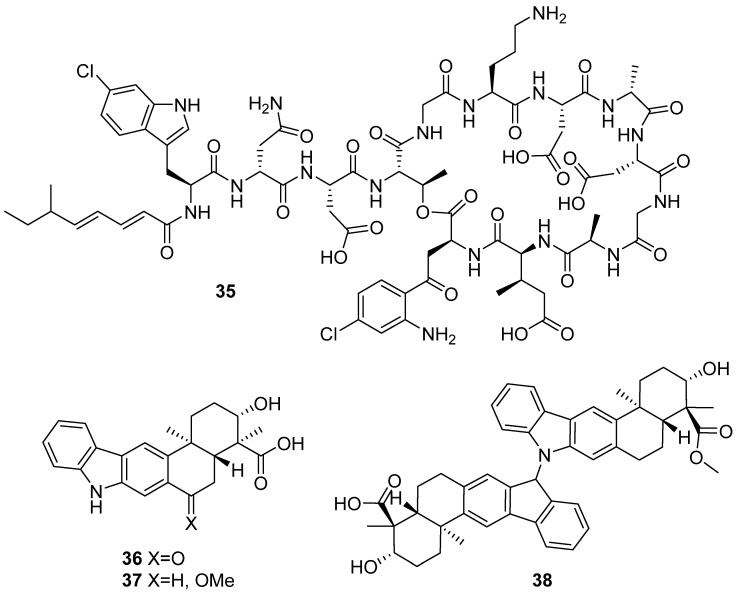
Chemical structures of **35**–**38**.

Taromycin B (**35**) was produced by heterologous expression of the activated taromycin biosynthetic gene clusters from marine actinomycete *Saccharomonospora* sp. CNQ-490. It showed potent activity against methicillin-resistant *Staphylococcus aureus* and vancomycin-resistant *Enterococcus faecium* (Figure 4) [29]. New xiamycin analogs (**36**–**38**) were isolated via genome mining of *Streptomyces xinghaiensis* NRRL B-24674^T^, and the bioactivity was not evaluated [30].

#### 2.1.2. Sponge-Sourced Bacteria

**Figure 5:**
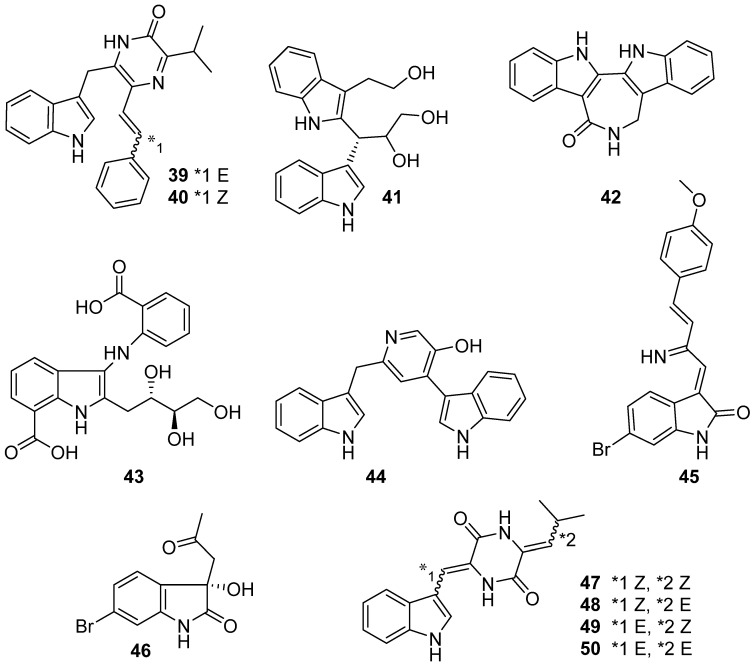
Chemical structures of **39**–**50**.

Enhypyrazinones A and B (**39** and **40**), from a marine-derived myxobacterium *Enhygromyxa* sp., showed weak activity (MIC values > 128 µg/mL) against *E. coli*, methicillin-resistant *Staphylococcus aureus* (MRSA), and methicillin-sensitive *Staphylococcus aureus* (MSSA) (Figure 5) [31]. Investigation of the bioactive secondary metabolites of the sponge-derived actinomycete *Rubrobacter radiotolerans* led to the isolation and characterization of another new naturally rare dimeric indole derivative (**41**). Compound **41** showed moderate antichlamydial activity with IC_50_ values of 46.5–96.4 μM against different *Chlamydia* [32]. Rhodozepinone (**42**), a new azepino-diindole alkaloid, was isolated and identified from the broth culture of *Rhodococcus* sp. UA13, which had been previously recovered from the Red Sea sponge *Callyspongia aff. Implexa.* Rhodozepinone (**42**) exhibited significant antibacterial and antitrypanosomal activities against *Staphylococcus aureus* NCTC 8325 (IC_50_ = 8.9 µg/mL) and *Trypanosoma brucei brucei* TC221 [IC_50_ = 16.3 (48 h) and 11.8 (72 h) µg/mL], respectively [33]. Anthranoside C (**43**), discovered from actinomycete *Streptomyces* sp. CMN-62, which was originated from a marine sponge, could inhibit influenza H1N1 virus with an IC_50_ value of 171 μM (ribavirin as positive control, IC_50_ = 133 μM) [34]. *Lysinibacillus fusiformis* was one of the 48 sponge-associated microbes identified from *Halichondria okadai* by testing 720 kinds of culture conditions. Lysiformine (**44**) was isolated from *Lysinibacillus fusiformis*, and displayed cytotoxicity toward mouse leukemia P388 cells with an estimated IC_50_ value of 10 μM [35]. Saccharomonosporine A (**45**), a novel brominated oxo-indole alkaloid and convolutamydine F (**46**) were isolated from *Saccharomonospora* sp. UR22 and *Dietzia* sp. UR66 co-culture. Compound **45** was a potential Pim-1 kinase inhibitor that mediate the tumor cell growth inhibitory effect with an IC_50_ value of 0.3 ± 0.02 µM on Pim-1 kinase and significant antiproliferative activity against HT-29, (IC_50_ = 3.6 µM) and HL-60, (IC_50_ = 2.8 µM) [36]. Photopiperazines A–D (**47**–**50**) were isolated from sponge-derived actinomycete AJS-327. The cytotoxicity of photopiperazines A–D mixture was evaluated on four cancer cell lines. It showed 12,000-fold selective toxicity toward U87 and SKOV3 than MDA-MB-231 and HCT-116 cell lines with the IC_50_ values of 0.41 nΜ and 0.75 nM, respectively [37].

#### 2.1.3. Miscellaneous

**Figure 6:**
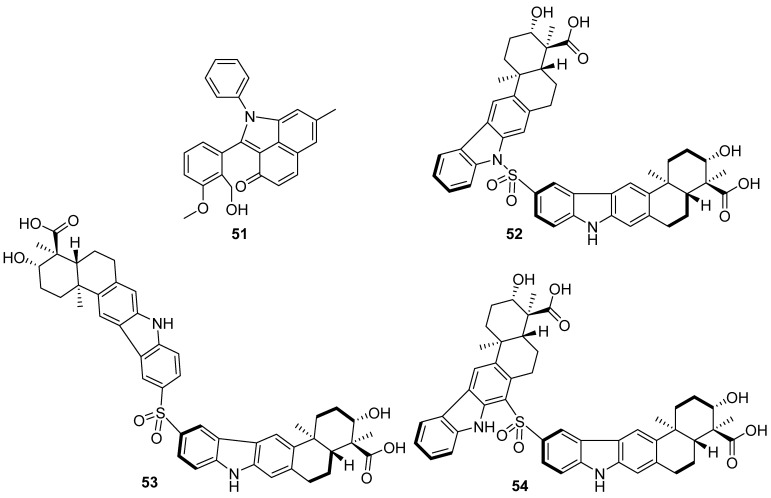
Chemical structures of **51**–**54**.

A new phenylamine-incorporated angucyclinone (**51**) was discovered from marine *Streptomyces* sp. PKU-MA00218 (Figure 6). Compound **51** was produced from a nonenzymatic conversion of the type II PKS-produced precursor. In addition, 18 new phenylamine-incorporated angucyclinone derivatives were prepared by the efficient nonenzymatic conversion under mild conditions. All 19 compounds showed different degrees of activity on nuclear factor erythroid 2-related factor 2 (Nrf2) transcription in HepG2 cells at 10 μM [38]. Sulfadixiamycins A–C (**52**–**54**) are a new class of antibiotics featuring sulfanilamide and dapsone substructures firstly reported from natural sources. They were discovered from recombinant *Streptomyces* species harboring the entire xiamycin biosynthesis gene cluster and exhibited moderate antimycobacterial activities and potent antibiotic activities even against multidrug-resistant bacteria [39].

**Figure 7:**
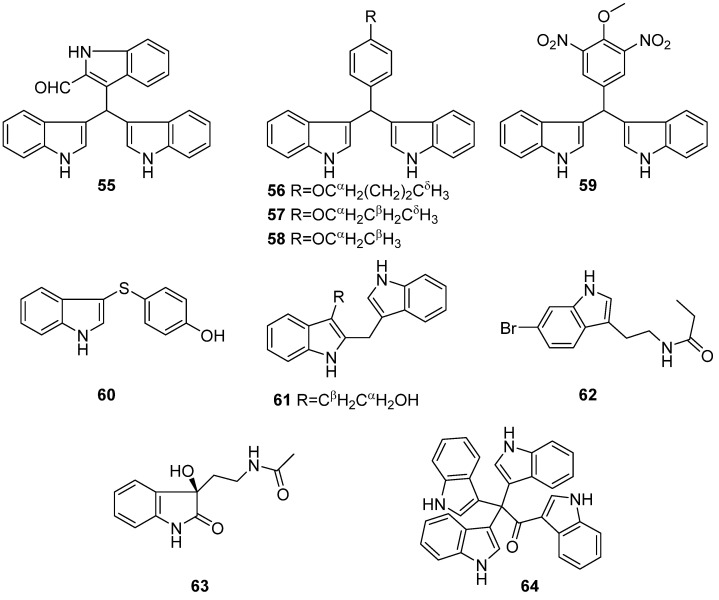
Chemical structures of **55**–**64**.

Two psychrotolerant bacterial strains *Vibrio splendidus* T262 and *Arthrobacter psychrochitiniphilus* T406 were isolated from the gastrointestinal tract of a fish and the excrement of penguins near the South Orkney Islands in Antarctica. Seven new indole alkaloids, trisindolal (**55**), turbomycin C–F (**56**–**59**), 4-(1H-indol-3-yl-sulfanyl) phenol (**60**), and 2-(indol-3-ylmethyl)-indol-3-ylethanol (**61**) were obtained from T262 and T406 (Figure 7). Trisindolal (**55**) was active against the peronosporomycetes *Botrytis cinerea* and *Phytophthora infestans*, and showed pronounced potency and selectivity in a panel of 11 human tumor cell lines derived from 10 different tumor histotypes [40]. 6-Bromo-N-propionyltryptamine (**62**) were isolated and identified from a marine bacterium *Pseudoalteromonas rubra* QD1-2 and exhibited weak 5-HT_2A_ receptor antagonist activity (~10% inhibition, 10 μM) [41]. Another simple indole alkaloid (**63**) was isolated from the deep-sea-derived bacterium *Bacillus subterrneus* 11593 and displayed no anti-allergic bioactivity [42]. Compound **64** was isolated from *Pseudovibrio denitrificans* strain isolated from seawater. It showed cytotoxic effect against L929 cells (EC_50_ = 7 μM) and A549 cells (EC_50_ = 8 μM) [43].

### 2.2. Marine-Sourced Fungi

Marine fungi are important components of marine microorganisms, and they are the main source of marine natural products. Among them, Cephalosporin C is the brightest star molecule as the first marine antibiotic [44,45]. In this part, 257 new indole alkaloids were summarized, including 93 from sediment-derived fungi, 62 from coral-derived fungi, 19 from bivalve-mollusk-derived fungi, 20 from Mangrove-sourced fungi, 16 from marine alga endophytic fungi, and 20 from sponge-sourced fungi.

#### 2.2.1. Sediment-Sourced Fungi

**Figure 8:**
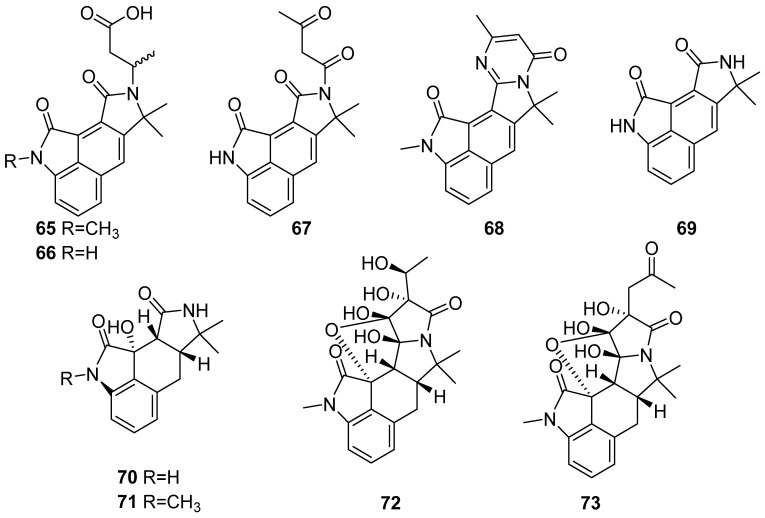
Chemical structures of **65**–**73**.

Cyclopiamides B–J (**65**–**73**), nine new oxindole alkaloids were isolated from the sediment-derived fungus *Penicllium commune* DFFSCS026 (−3563 m in the South China Sea) (Figure 8). Compounds **65**–**73** and positive control (ochratoxin A) displayed lethal activity on brine shrimp with LC_50_ values of 25.2, 38.5, 14.1, 24.8, 25.6, 34.7, 16.4, 33.5, 26.7 and 6.2 mg/mL, respectively. Compounds **65**–**73** showed no cytotoxicity towards human carcinoma HepG-2 and HeLa cell lines at a concentration of 100 mg/mL, and no anti-influenza virus H1N1 activity under their largest concentration of non-toxic towards the tested MDCK cell [46]. 

**Figure 9:**
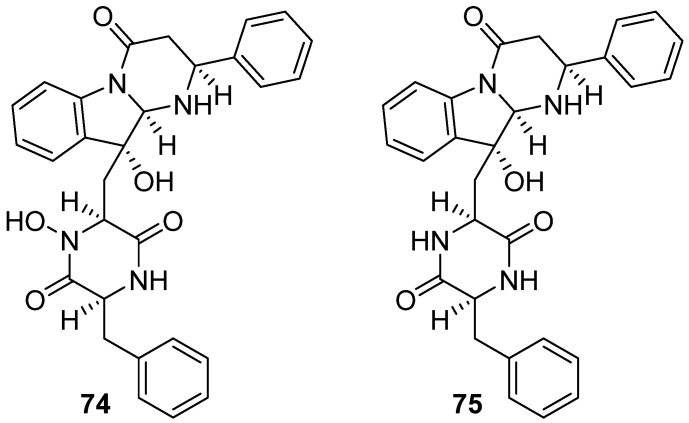
Chemical structures of **74**–**75**.

Haenamindole (**74**) and citreoindole (**75**) were isolated from a South China Sea deep-sea fungus, *Penicillium citrinum* MF006 (Figure 9) [47]. The structures of the rare alkaloids **74** and **75** were revised based on detailed spectroscopic and C_3_ Marfey’s analysis [48,49]. 

**Figure 10:**
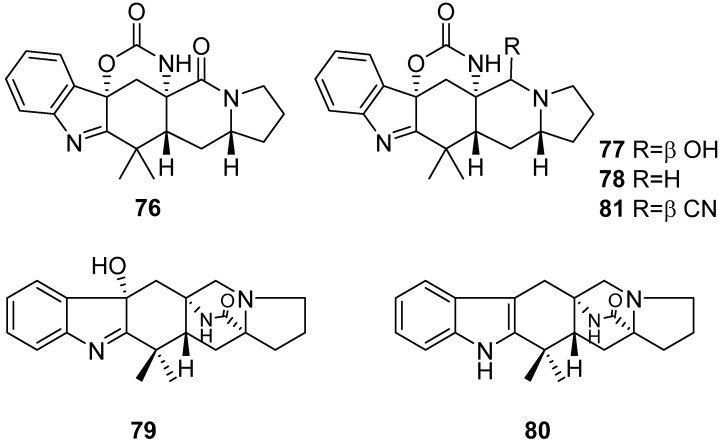
Chemical structures of **76**–**81**.

Penicimutamides A–E (**76**–**80**) and a structure-revised aspeverin (**81**), six new prenylated indole alkaloids, were isolated from a diethyl sulfate mutagenesis mutant of the marine-derived fungus *Penicillium purpurogenum* G59. Compound **76** and compounds **78**–**80** displayed less than 28.5% inhibition rates on human K562, HL-60, HeLa and BGC-823 cell lines at the concentration of 100 µg/mL, while compound **77** showed remarkable inhibition rates (77.3–92.7% at 100 µg/mL) and the further determined IC_50_ values ranged from 20 to 52 µg mL^−1^ (Figure 10) [50,51]. 

**Figure 11:**
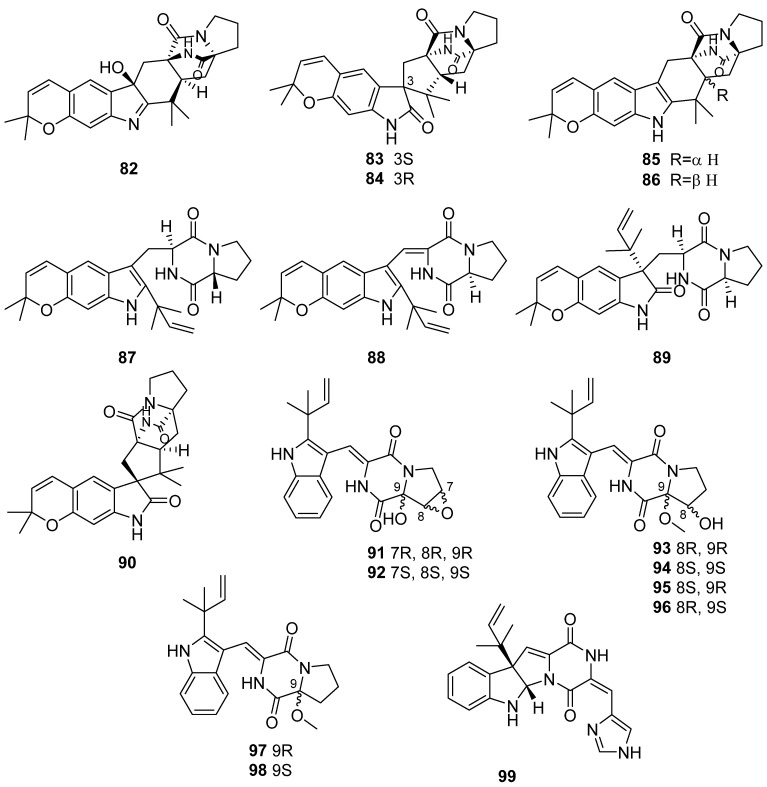
Chemical structures of **82**–**99**.

Asperversiamides A–H (**82**–**89**) and structure-revised iso-notoamide B (**90**) were isolated from the marine-derived fungus *Aspergillus versicolor* (Figure 11). Asperversiamide G (**88**) exhibited a potent anti-inflammatory activity with an IC_50_ value of 5.39 μM against iNOS (Figure 10) [52]. Eight new diketopiperazines (**91**–**98**) were isolated from a marine-derived fungus *Aspergillus versicolor* MF180151, and they showed no activity against the tested pathogens (*Candida albicans*, *Bacillus subtilis*, *Staphylococcus aureus*, *Methicillin-resistant S. aureus*, *Pseudomonas aeruginosa*, and *Bacillus Calmette-Guérin*) [53]. Roquefortine J (**99**) was founded in *Penicillium granulatum* MCCC 3A00475 and with an IC_50_ value of 19.5 μM against HepG2 tumor cells [54].

**Figure 12:**
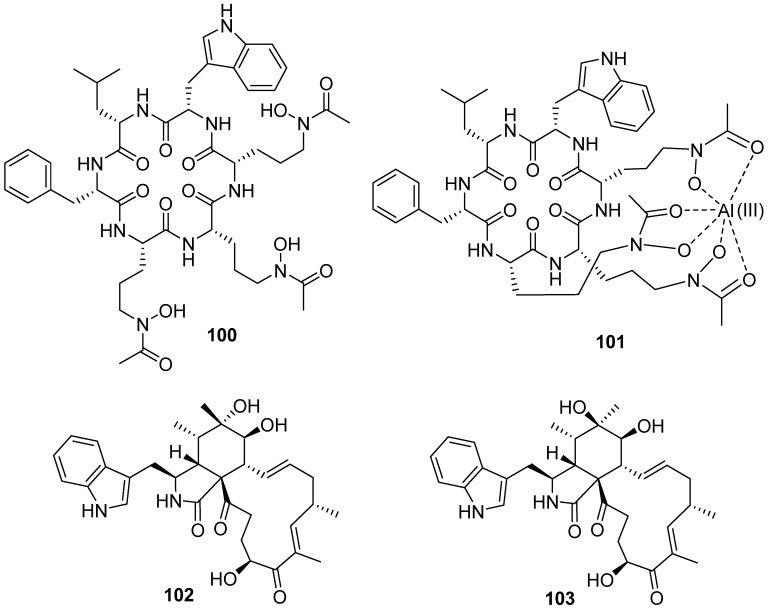
Chemical structures of **100**–**103**.

Acremonpeptide D (**100**), together with Al(III)-acremonpeptide D (**101**) were obtained from the marine fungus *Acremonium persicinum* SCSIO 115 (Figure 12). In vitro bioassays revealed Al(III)-acremonpeptide D (**101**) as moderate antiviral agents for herpes simplex virus 1 with an EC_50_ value of 14 μM (Figure 11) [55]. Cytoglobosins H (**102**) and I (**103**) were isolated from the deep-sea-derived fungus *Chaetomium globosum*, which was obtained from a deep-sea sediment sample (−2500 m depth) of the Indian Ocean. They showed weak to no cytotoxicity against MDA-MB-231, LNCaP and B16-F10 cell lines (IC_50_ > 9 μM) [56].

**Figure 13:**
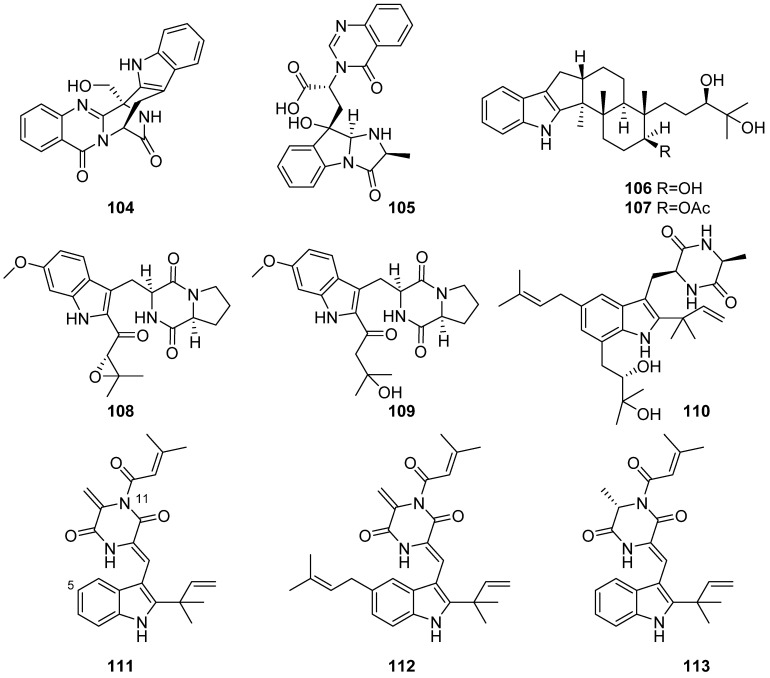
Chemical structures of **104**–**113**.

Two alkaloids, fumigatosides E (**104**) and F (**105**) were isolated from deep-sea derived fungal *Aspergillus fumigatus* SCSIO 41012 (Figure 13). Compound **104** showed significant antifungal activity against *Fusarium oxysporum* f. sp. *momordicae* with MIC at 1.56 µg/mL, and compound **105** exhibited significant activity against *A. baumanii* with a MIC value of 6.25 µg/mL [57]. Penijanthines C and D (**106** and **107**) were isolated from the marine-derived fungus *Penicillium janthinellum*. They displayed significant anti-*Vibrio* activity (MIC values ranging from 3.1 to 50.0 µM) against three pathogenic *Vibrio* spp. [58]. Two new compounds 19S,20-epoxy-18-oxotryprostatin A (**108**) and 20-hydroxy-18-oxotryprostatin A (**109**) were discovered from the marine-derived fungus *Aspergillus fumigatus* MF071 from the Bohai Sea sediment. For the limited amounts of **108** and **109**, no activities have been evaluated yet [59]. Four indole diketopiperazine alkaloids aspechinulins A–D (**111**–**113** and **110**) were isolated from the sediment-derived fungus *Aspergillus* sp. FS445. Compounds **111**–**113** represented the first examples of indole diketopiperazine derivatives constructing a C-5 unit at 11-NH through an imide linkage. Compound **113** exhibited the most potent inhibitory activities against NO production with the IC_50_ value of 20 μM, which was as effective as the positive control aminoguanidine (IC_50_ = 23.7 μM) [60]. 

**Figure 14:**
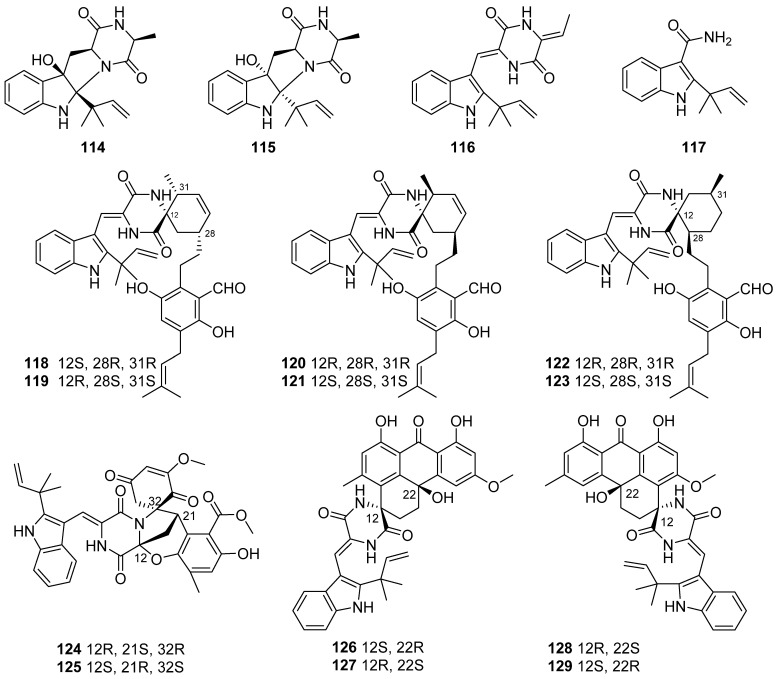
Chemical structures of **114**–**129**.

Three new prenylated indole 2,5-diketopiperazine alkaloids (**114**–**116**), one new indole alkaloid (**117**), and six pairs of new spirocyclic alkaloid enantiomers eurotinoids A–C (**118**–**123**) and variecolortins A–C (**124**–**129**) were characterized from the sediment-derived fungus *Eurotium* sp. SCSIO F452 (Figure 14). Compound **116** and all the spirocyclic alkaloids **118**–**123** showed significant radical scavenging activities against DPPH with IC_50_ values ranging from 3.7 to 24.9 µM. None of the alkaloids (**114**–**129**) exhibited obvious cytotoxicity against SF-268 and HepG2 cell lines. Interestingly, (+)-enantiomers (**118**, **120** and **122**) exhibited more potent activities than the corresponding (−)-enantiomers (**119**, **121** and **123**). (+)-enantiomer **124** exhibited stronger antioxidative activity than (−)-enantiomer **125**, while (+)-enantiomers (**126** and **128**) showed more potent cytotoxicities against SF-268 and HepG2 cell lines than (−)-enantiomers (**127** and **129**), which indicated that different enantiomers might result in different biological activities [61,62,63]. 

**Figure 15:**
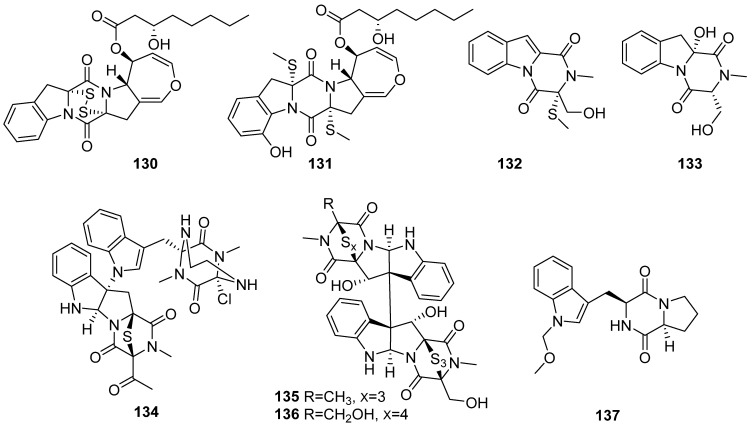
Chemical structures of **130**–**137**.

Graphiumins I (**130**) and J (**131**) were isolated from the culture broth of the marine-derived fungus *Graphium* sp. OPMF00224 (Figure 15). Compounds **130** and **131** inhibited yellow pigment production by MRSA with IC_50_ values of 63.5 and 76.5 µg/mL, respectively, without inhibiting its growth, even at 250 µg/mL [64]. Dichotocejpins A and C (**132** and **133**) were isolated from the culture of the deep-sea sediment-derived fungus *Dichotomomyces cejpii* FS110. Compound **132** exhibited excellent inhibitory activity against α-glucosidase with an IC_50_ of 138 µM [65]. Cristazine (**134**) was isolated from the mudflat-sediment-derived fungus *Chaetomium cristatum*. It displayed potent radical-scavenging activity against 2,2-diphenyl-1-picrylhydrazyl (DPPH), with IC_50_ values of 19 μM, and cytotoxic activity against HeLa cells, with an IC_50_ value of 0.5 μM [66]. Chetracins E and F (**135** and **136**) were isolated from the fungus *Acrostalagmus luteoalbus* HDN13-530 and showed potent cytotoxic effects on A549, HCT-116, K562, H1975 and HL-60 with IC_50_ values ranging from 0.2 to 3.6 µM [67]. In the study of fungal and bacterial co-cultivation, a new indole alkaloid brevianamide X (**137**) was isolated from the *Aspergillus fumigatus* MR2012 fermentation [68].

**Figure 16:**
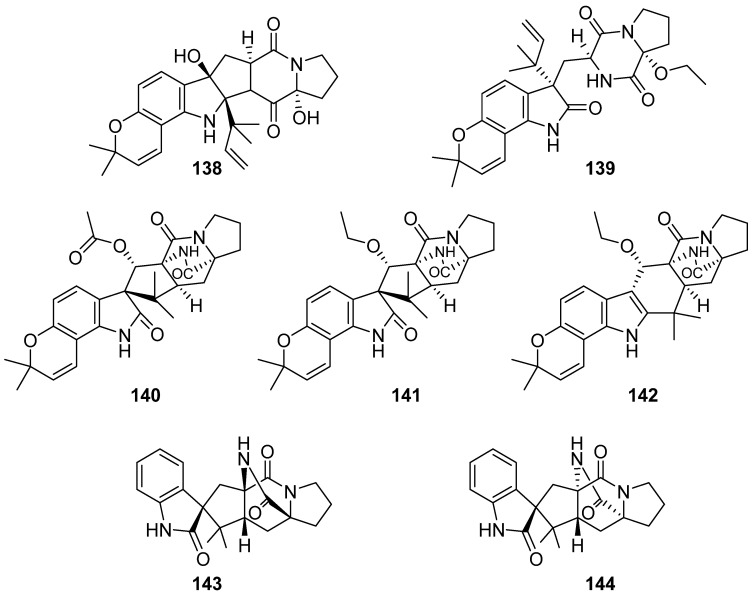
Chemical structures of **138**–**144**.

Five new prenylated indole alkaloids, 17-hydroxynotoamide D (**138**), 17-O-ethylnotoamide M (**139**), 10-O-acetylsclerotiamide (**140**), 10-O-ethylsclerotiamide (**141**) and 10-O-ethylnotoamide R (**142**) were isolated from the co-culture of marine-sediment-derived fungi *Aspergillus sulphureus* KMM 4640 and *Isaria felina* KMM 4639 (Figure 16). Compound **139** inhibited the colony formation of human prostate cancer cells 22Rv1 at non-cytotoxic concentration of 10 μM [69]. Two brevianamides (**143** and **144**) were isolated from the deep-sea-derived fungus *Penicillium brevicompactum* DFFSCS025. Both of them exhibited no cytotoxicity against HCT116 and no antibacterial or antifungal activities against *Streptococcus mutans*, *S. sobrinus* and *Fusarium oxysporum* f. sp. *cubense* Race 1 and Race 4. Compound **143** showed no antilarval activity in the larval settlement bioassay [70].

**Figure 17:**
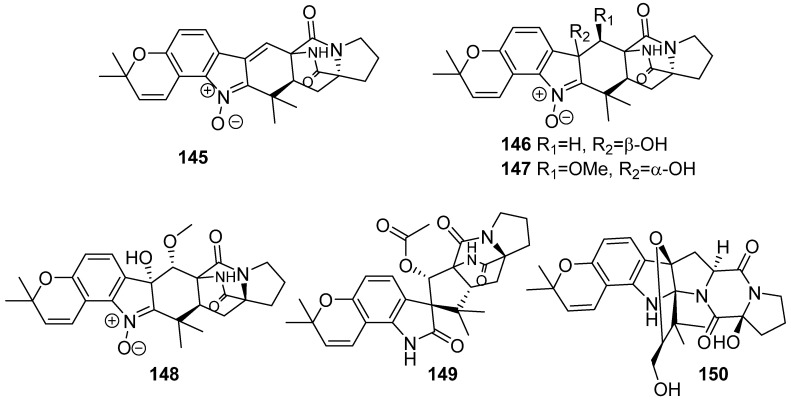
Chemical structures of **145**–**150**.

Six new prenylated indole diketopiperazine alkaloids, asperthrins A–F (**145**–**150**), were isolated from the marine-derived fungus *Aspergillus* sp. YJ191021 (Figure 17). Compound **145** exhibited moderate antifungal and antibacterial activities against *Vibrio anguillarum*, *Xanthomonas oryzae pv. Oryzicola*, and *Rhizoctonia solani* with MIC values of 8, 12.5, and 25 µg/mL, respectively. Furthermore, **145** displayed notable anti-inflammatory activity with an IC_50_ value of 1.46 ± 0.21 µM in *Propionibacterium acnes* induced human monocyte cell line (THP-1) [71]. 

**Figure 18:**
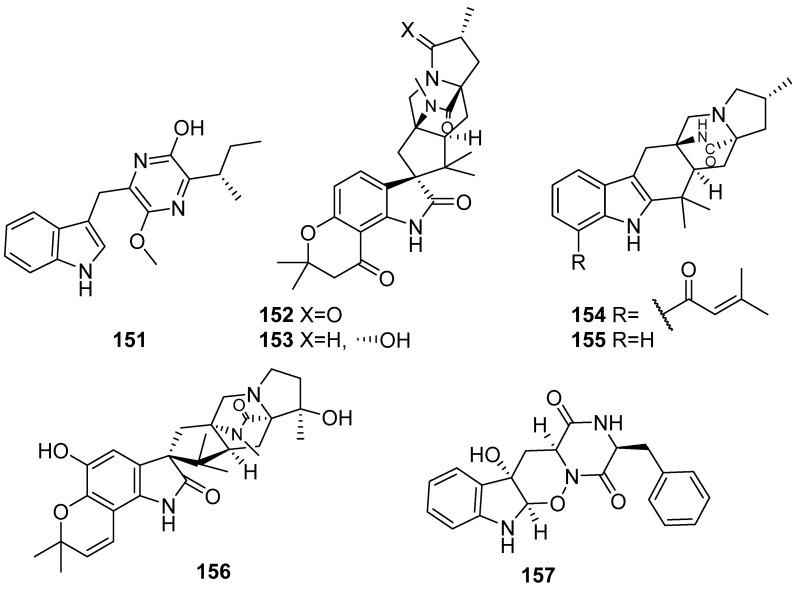
Chemical structures of **151**–**157**.

Seven new indole marine natural products (**151**–**157**) were isolated from four mangrove swamp-derived fungi (Figure 18). Trypilepyrazinol (**151**) was isolated from the fungus *Penicillium* sp. IMB17-046. Trypilepyrazinol exhibited inhibitory activities against HIV-1 and HCV with IC_50_ values of 4.6 and 7.7 µM, respectively. It also showed antibacterial activities against *Helicobacter pylori*. (including the drug-sensitive strain G27 and the drug-resistant strain 159), but inactive against Gram-positive *Staphylococcus aureus* and *Bacillus subtilis* and *Gram-negative Pseudomonas aeruginosa* and *Klebsiella pneumonia* [72]. Four new prenylated indole alkaloids (**152**–**155**) were isolated from *Penicillium* sp. SCSIO041218 and inactive to the tested anti-allergic bioactivity on IgE-mediated rat mast RBL-2H3 cells [73]. A new prenylated indole alkaloid, named paraherquamide J (**156**), was isolated from another mangrove rhizosphere soil-derived fungus *Penicillium janthinellum* HK1-6. No activity was found for the tested antibacterial, topoisomerase I (topo I) inhibitory activities and lethality towards brine shrimp *Artemia salina* [74]. Raistrickindole A (**157**), a new indole diketopiperazine alkaloid, was isolated from the fungus *Penicillium raistrickii* IMB17-034. Compounds **157** showed anti-HCV activity with an EC_50_ value of 5.7 μM in the in vitro inhibitory assay against the hepatitis C virus life cycle [75].

#### 2.2.2. Coral-Sourced Fungi

**Figure 19:**
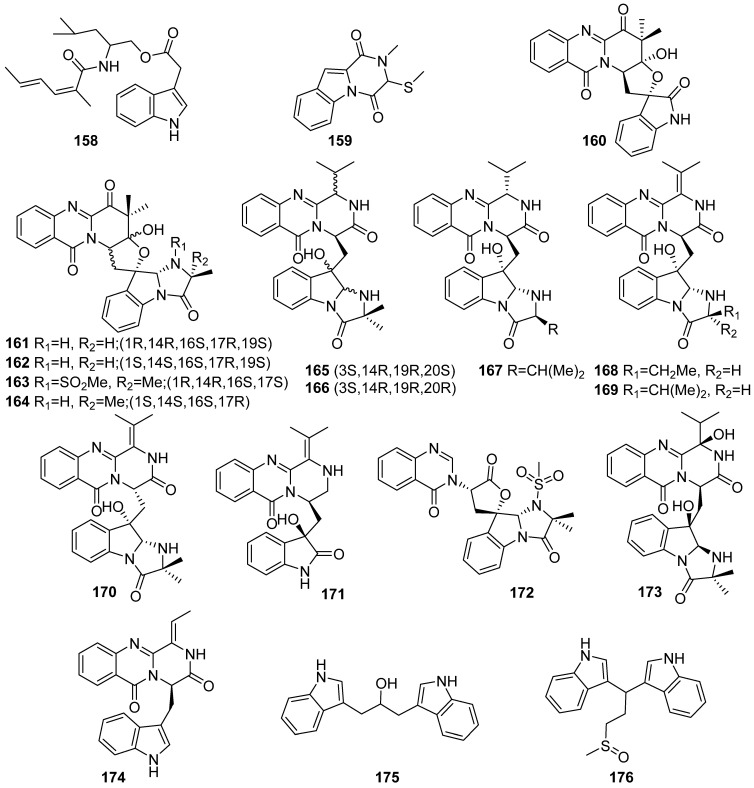
Chemical structures of **158**–**176**.

Wen-Jian Lan and co-workers have conducted a great deal of research focused on the chemical diversity of fungi associated with soft coral. In recent years, nineteen marine indole alkaloids (**158**–**176**) have been identified by an amino acid-directed strategy, which is a method of feeding various amino acids to marine fungi (Figure 19). Utilizing this strategy, dichotomocej D (**158**) and dichocerazine A (**159**) were isolated from L-tryptophan and L-phenylalanine fed *Dichotomomyces cejpii* F31-1, scedapins A–E (**161**–**164**, **160**) and scequinadolines A–G (**165**–**171**), scetryptoquivaline A (**172**), scequinadoline I (**173**), and scequinadoline J (**174**) were isolated from *Scedosporium apiospermum* F41-1 by the same amino acid-directed strategy. Another two new bisindole alkaloids, pseudoindoles A–B (**175**–**176**), were isolated from L-tryptophan, L-phenylalanine, L-methionine, and L-threonine fed *Pseudallescheria boydii* F44-1. Among these compounds, scedapin C (**163**) and scequinadoline D (**168**) displayed significant antiviral activity against hepatitis C and scequinadoline J (**174**), and they promote triglyceride accumulation in 3T3-L1 cells [76,77,78,79]. 

**Figure 20:**
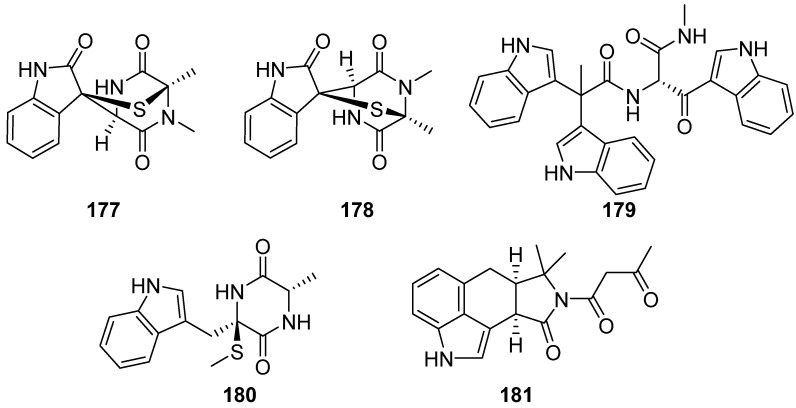
Chemical structures of **177**–**181**.

Between the years 2015 and 2016, Wen-Jian Lan and co-workers also isolated five indole alkaloids (**177**–**181**) directly from coral-derived fungi, including Pseudellones A–D (**177**–**180**) from *Pseudallescheria ellipsoidea* F42-3 and **181** from *Pseudallescheria boydii*. No significant tested bioactivity was reported (Figure 20) [80,81,82].

**Figure 21:**
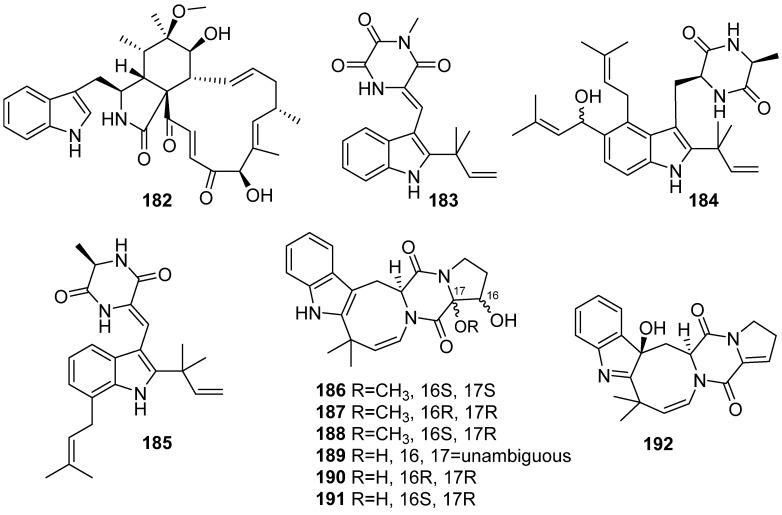
Chemical structures of **182**–**192**.

A new cytochalasin, 6-O-methyl-chaetoglobosin Q (**182**), was isolated from the coral-associated fungus *Chaetomium globosum* C2F17, and no bioactivity was reported (Figure 21) [83]. Three new indole diketopiperazine alkaloids, 11-methylneoechinulin E (**183**), variecolorin M (**184**) and (+)-variecolorin G (**185**) were isolated from a soft coral-associated epiphytic fungus *Aspergillus* sp. EGF 15-0-3. They all have no in vitro toxicity against NCI-H1975/GR cell line at the concentration of 50 µM [84]. Seven new deoxyisoaustamide derivatives (**186**–**192**) were isolated from the coral-derived fungus *Penicillium dimorphosporum* KMM 4689. Compounds **189**–**191** revealed a statistical increase in PQ (paraquat)-treated Neuro-2a cell viability by 30–39% at a concentration of 1 µM [85]. 

**Figure 22:**
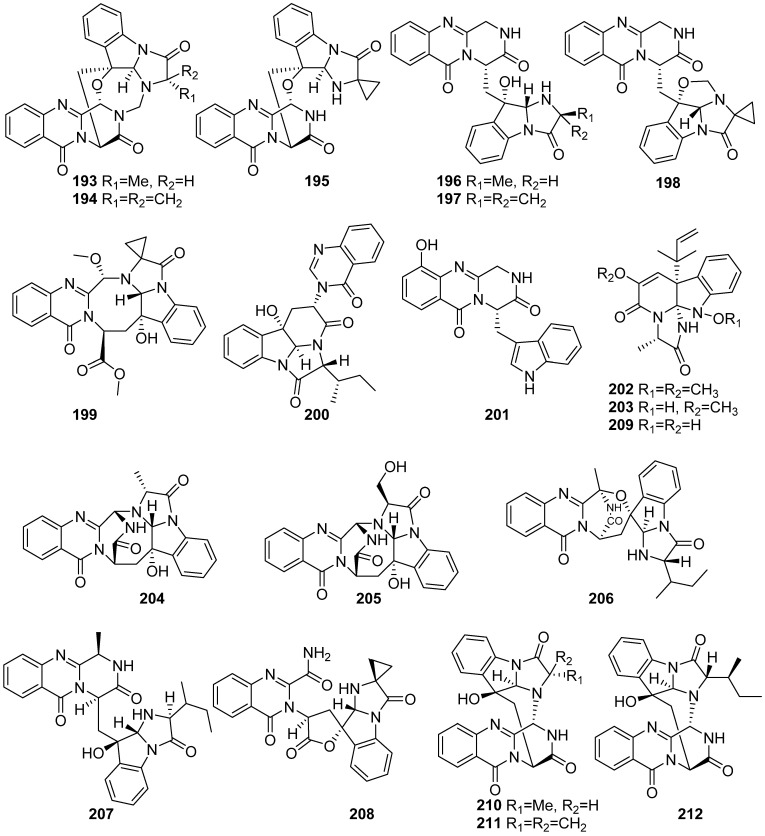
Chemical structures of **193**–**212**.

Seventeen fumiquinazoline-type alkaloids, versiquinazolines A–Q (**193**–**209**), were isolated from the gorgonian-derived fungus *Aspergillus versicolor* LZD-14-1 and the structures of cottoquinazolines B, D and C (**210**–**212**) were revised to enantiomers (Figure 22). Compounds **193**, **194**, **199**, **203**, **208** and **209** exhibited inhibitory activities against thioredoxin reductase (IC_50_ values ranging from 12 to 20 μM) [86,87]. 

**Figure 23:**
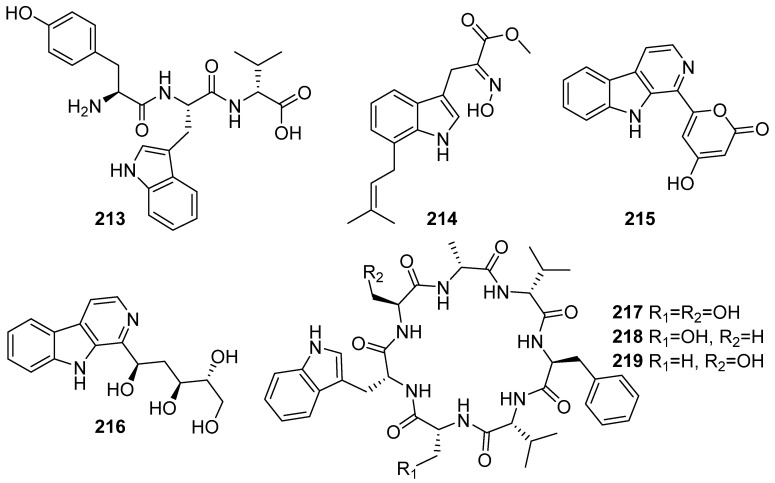
Chemical structures of **213**–**219**.

Aspergillipeptide E (**213**) was isolated from *Aspergillus* sp. SCSIO 41501 and **213** showed evident antiviral activity against herpes simplex virus type 1 (HSV-1) with an IC_50_ value of 19.8 μM under the non-cytotoxic concentrations against a Vero cell line (Figure 23) [88]. Luteoride E (**214**) was isolated and identified from a coral-associated fungus *Aspergillus terreus*. Luteoride E inhibited no α-Glucosidase inhibitory activity and with moderate inhibitory activity against LPS-induced NO production [89]. Aspergillspins A-B (**215**–**216**) were isolated from the marine gorgonian-derived fungus *Aspergillus* sp. SCSIO 41501. They exhibited no cytotoxicity activities against the tested HL-60, HepG2 and MCF-7 cell lines and no antibacterial activities against *Bacillus subtilis* and *E. coli* [90]. Three new cycloheptapeptides, asperversiamides A–C (**217**–**219**), were isolated from coral-derived fungus *Aspergillus versicolor* CHNSCLM-0063 under the guidance of molecular networking and ^1^H NMR. Asperversiamides A–C (**217**–**219**) exhibited potent inhibitory activity against *M. marinum* [91].

#### 2.2.3. Mollusk-Sourced Fungi

**Figure 24:**
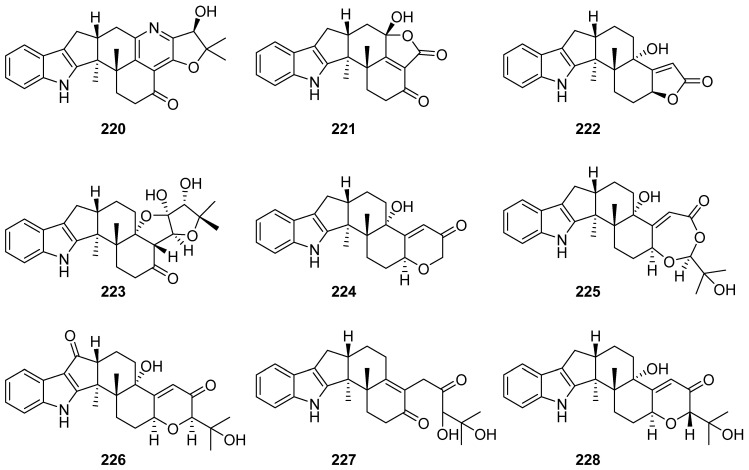
Chemical structures of **220**–**228**.

From the *Penicillium* sp. KFD28, an endophytic fungus in a bivalve mollusk *Meretrix lusoria* collected from Haikou Bay, eleven new indole-diterpenoids named penerpenes B–H and J (**220**–**226** and **227**) and epipaxilline (**228**) were isolated in Du-Qiang Luo and You-Xing Zhao groups (Figure 24). All the compounds showed potent to moderate inhibitory activity toward protein tyrosine phosphatase PTP1B with IC_50_ ranging from 1.7 to 31.8 μM. Compounds **220**, **226** and **227** showed inhibitory activity toward protein tyrosine phosphatase TCPTP with IC_50_ values of 5.0, 4.5, 35 and 14.7 μM, respectively [92,93,94]. 

**Figure 25:**
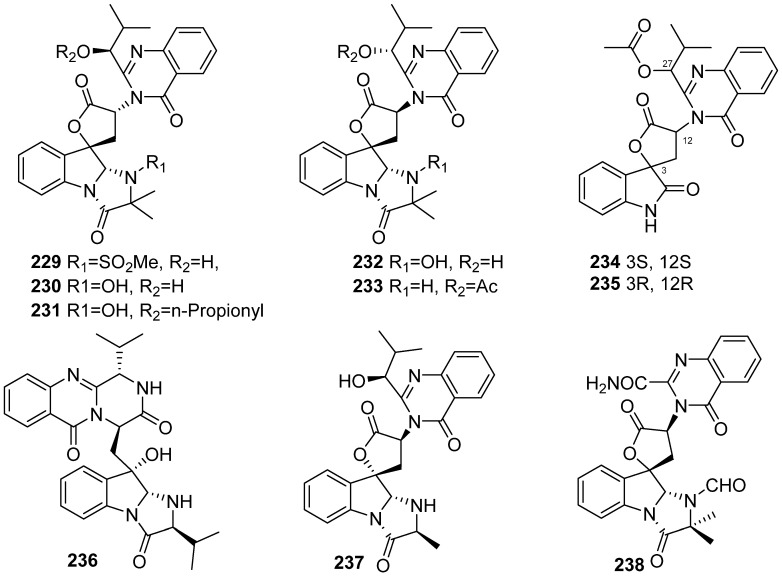
Chemical structures of **229**–**238**.

Seven new quinazoline-containing indole alkaloids named aspertoryadins A–G (**229**, **230**, **232**, **233**, **231**, **234** and **235**) were isolated from the marine-derived fungus *Aspergillus* sp. HNMF114, which was separated from the bivalve mollusk *Sanguinolaria chinensis* (Figure 25). Compound **229** bears an aminosulfonyl group in the structure, which is rarely encountered in natural products. Compounds **234** and **235** exhibited quorum sensing inhibitory activity against *Chromobacterium violaceum* CV026 with MIC values of 32, 32 and 16 μg/well, respectively [95]. A continuous work by feeding tryptophan to the marine-derived fungus *Aspergillus* sp. HNMF114 was carried out, another three new quinazoline-containing indole alkaloids aspertoryadins H–J (**236**–**238**) were obtained. The biological activity of these compounds against the insect ryanodine receptor (RyR) was tested using HEK cells stably expressing RyR from *Spodoptera frugiperda* (sfRyR) or RyR1 from rabbit (rRyR1) and R-CEPIA1er. Alkaloids **236**–**238** only showed a weak activation effect on sfRyR, which reduced the [Ca^2+^]_ER_ by less than 7% [96]. 

#### 2.2.4. Mangrove-Sourced Fungi

**Figure 26:**
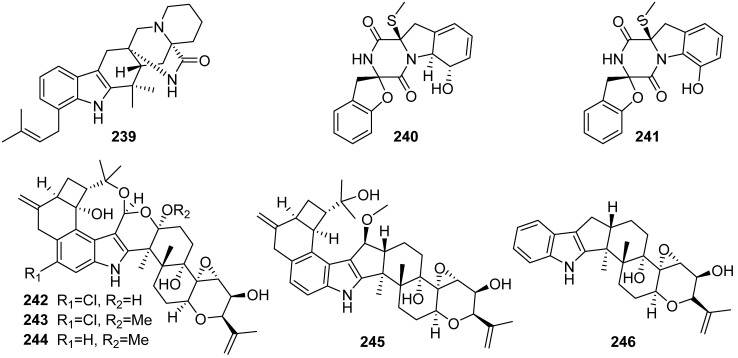
Chemical structures of **239**–**246**.

One of Bin-Gui Wang group’s studies focusing on the exploration of structurally unique and biologically active natural products from the mangrove-derived fungi isolated ten new indole alkaloids (Figure 26). Penioxamide A (**239**), a new prenylated indole alkaloid possessing a piperidine moiety, was isolated and identified from *Penicillium oxalicum* EN-201, an endophytic fungus obtained from the inner tissue of the fresh leaves of marine mangrove plant *Rhizophora stylosa*. Compound **239** bore the rare anti-relative configuration in the bicyclo[2.2.2]diazaoctane ring and showed potent brine shrimp lethality with LD_50_ values of 5.6 μM [97]. Three new diketopiperazines, including spirobrocazines A–B (**240**–**241**), were characterized from the mangrove-derived *Penicillium brocae* MA-231. Both **240** and **241** possess a 6/5/6/5/6 cyclic system. Compound **240** showed moderate and nonselective antimicrobial activity against *E. coli*, *S. aureus* and *Vibrio harveyi*, with MIC values of 32.0, 16.0 and 64.0 μg/mL, respectively, whereas no bioactivity was reported for the dearomatized **241** [98]. Six new indole-diterpenes rhizovarins A–C, E and F (**242**–**244**, **245** and **246**) were identified from *M. irregularis* QEN-189, an endophytic fungus isolated from the fresh inner tissue of the marine mangrove plant *Rhizophora stylosa*. Compounds **242** and **243** showed activities against human A549 and HL-60 cancer cell lines with IC_50_ values ranging from 5.0 to 11.5 μM, and compound **245** exhibited activity against the A-549 cancer cell line with an IC_50_ value of 9.2 μM [99].

**Figure 27:**
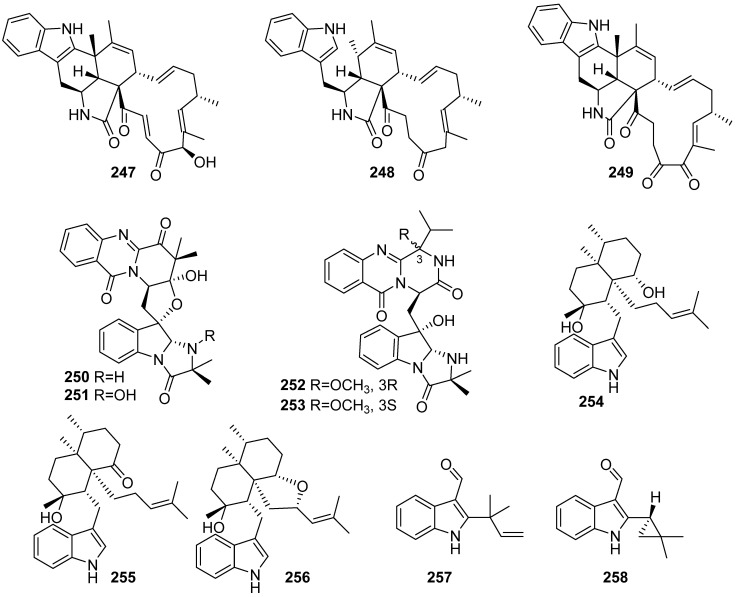
Chemical structures of **247**–**258**.

Three novel chaetoglobosins, named penochalasins I–K (**247**–**249**), were isolated from the culture of *Penicillium chrysogenum* V11 (Figure 27). Compound **248** greatly inhibited *C. gloeosporioides* (MIC = 25.08 µM), showing an antifungal activity higher than carbendazim. Compound **247** exhibited marked cytotoxicity against MDA-MB-435 and SGC-7901 cells (IC_50_ < 10 µM) [100]. Compound **249** displayed significant inhibitory activities against *C. gloeosporioides* and *R. solani* (MICs = 6.13, 12.26 μM, respectively), and it also exhibited potent cytotoxicity against MDA-MB-435, SGC-7901 and A549 cells (IC_50_ < 10 μM) [101]. Neosartoryadins A (**250**) and B (**251**), with a unique 6/6/6/5 quinazoline ring system connected directly to a 6/5/5 imidazoindolone ring, together with compounds **252** and **253** were isolated from the endophytic fungus *Neosartorya udagawae* HDN13-313. Compounds **250** and **251** exhibited inhibitory effects against influenza A virus (H1N1) with IC_50_ values of 66 μM and 58 μM by the cytopathic effect (CPE) inhibition assay, which were smaller than the positive control ribavirin with the IC_50_ of 94 μM [102]. Three new indole diterpenes, penicilindoles A–C (**254**–**256**), were isolated from the mangrove-derived fungus *Eupenicillium* sp. HJ002. After evaluating cytotoxic and antibacterial activities in vitro, penicilindole A (**254**) showed cytotoxic activity against human A549 and HepG-2 cell lines with IC_50_ values of 5.5 and 1.5 μM, respectively [103]. Two prenylated indole 3-carbaldehydes (**257** and **258**) were purified from mangrove-derived endophytic fungus *Eurotium* *chevalieri* KUFA 0006, and they significantly inhibited the biofilm production in *S. aureus* [104].

#### 2.2.5. Alga-Sourced Fungi

**Figure 28:**
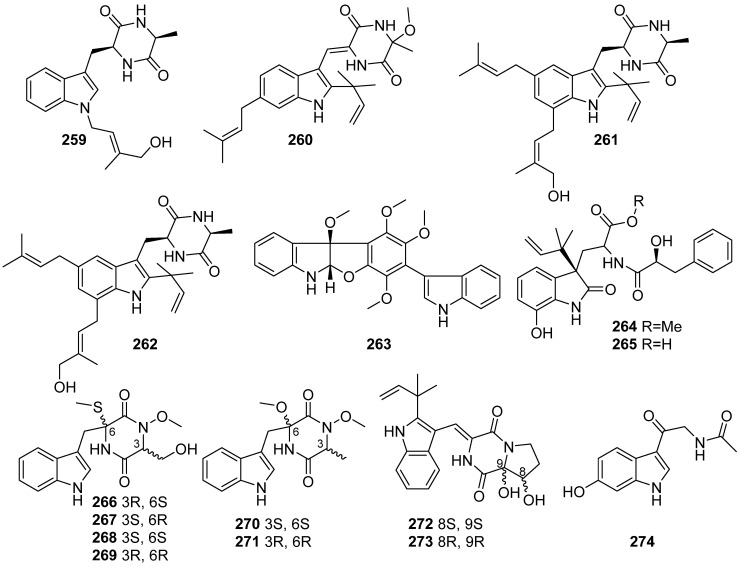
Chemical structures of **259**–**274**.

Bin-Gui Wang’s group investigated the bioactive secondary metabolites of marine alga endophytic fungus. Thirteen new indole alkaloids were isolated, including four indolediketopiperazine alkaloids (**259**–**262**) from *Eurotium cristatum* EN-220, varioloid C (**263**) from *Paecilomyces variotii* EN-291, 4-epi-seco-shornephine A methyl ester (**264**) and 4-epi-seco-shornephine A carboxylic acid (**265**) from *Aspergillus alabamensis* EN-547 and three pairs of new N-methoxy-containing indolediketopiperazine enantiomers, acrozines A–C (**266**–**271**) from *Acrostalagmus luteoalbus* TK-43 (Figure 28). Compound **260** exhibited potent lethal activity against brine shrimp (LD_50_ = 19.4 μg/mL) and weak nematicidal effect against *Panagrellus redivivus* (LD_50_ = 110.3 μg/mL). Compound **263** exhibited cytotoxicity against A549, HCT116 and HepG-2 cell lines (2.5–6.4 μg/mL). Compounds **264**–**265** showed inhibitions against human pathogens *E. coli* and *M. luteus* and aquatic bacteria *Ed. ictaluri* and *V. alginolyticus* with MIC values ranging from 16 to 64 µg/mL [105,106,107,108,109]. **272** and **273** were isolated from chemical-epigenetic cultures of *Aspergillus versicolor* OUCMDZ-2738 with 10 µM vorinostat (SAHA), and no antibacterial activity against the eight tested pathogenic microorganisms [108]. A new melatonin analog 6-hydroxy-N-acetyl-β-oxotryptamine (**274**) was isolated from the marine-derived fungus *Penicillium* sp. KMM 4672. It was not cytotoxic against neuroblastoma Neuro2a cells up to 100 µM and scavenged DPPH radicals by 48% at 100 µM. Compound **274** demonstrated increased cell viability in both 6-OHDA and PQ-induced neuronal cell damage models [110].

#### 2.2.6. Sponge-Sourced Fungi 

**Figure 29:**
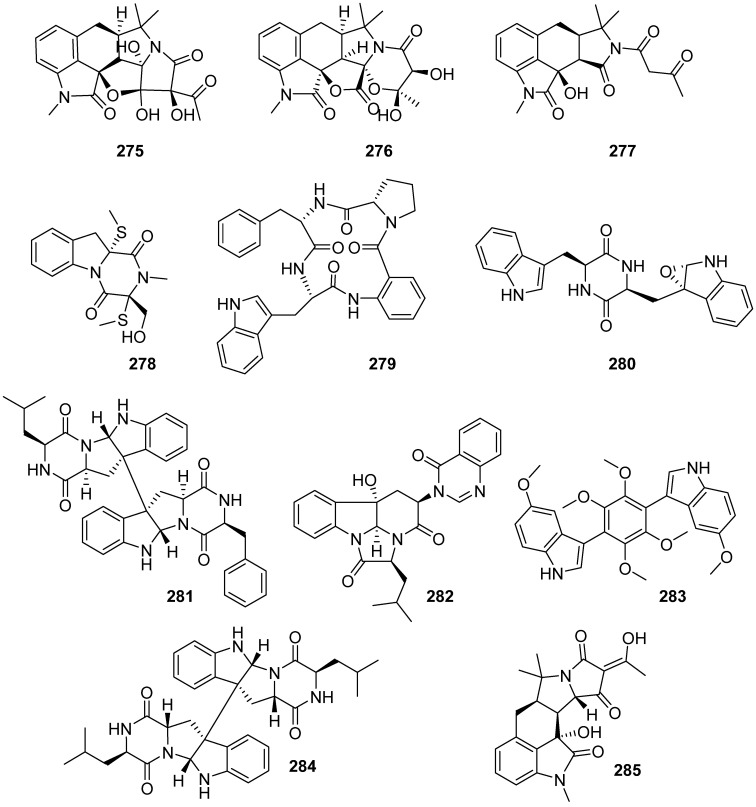
Chemical structures of **275**–**285**.

Speradines B–D (**275**–**277**) were isolated from the sponge-derived fungus *Aspergillus flavus* MXH-X104 (Figure 29). Oxindoles **275**–**277** showed no activities on all the bioassay (cytotoxicities on P388, BEL-7402, A-549, Hela and HL-60 cells; inhibitory effects on H1N1 and HIV viruses, and antimicrobial activities on *Mycobacterium phlei*, *Staphylococcus aureus*, *Colibacillus sp.* and *Blastomyces albicans*) [111]. Methylthio-gliotoxin derivative **278** was firstly characteried from a sponge-derived fungus *Ascomycota Dichotomomyces cejpii* and the fungus was isolated from sponge *Callyspongia* cf. *C. flammea*. This study validates the anti-proliferative mechanisms of the newly isolated natural epipolythiodiketopiperazines via the inhibition of TNFα-induced NF-κB activity [112]. Sartoryglabramide B (**279**) and fellutanine A (**280**) were isolated from sponge-associated fungus *Neosartorya glabra* KUFA 0702, and they exhibited no antibacterial and antifungal activities [113]. A new diketopiperazine dimer designated as SF5280-415 (**281**) was isolated from an ethyl acetate extract of the marine-derived fungus *Aspergillus* sp. SF-5280. Compound **281** showed inhibitory effects against protein tyrosine phosphatase 1b (PTP1B) with an IC_50_ value of 14.2 ± 0.7 μM [114]. Isopropylchaetominine (**282**) was isolated from fungus *Aspergillus carneus* using the OSMAC (one strain many compounds) approach, and it showed potent cytotoxicity against the mouse lymphoma cell line L5178Y with IC_50_ values of 0.4 μM [115]. Candidusin D (**283**) was isolated from the cultures of the marine sponge-associated fungus *Aspergillus candidus* KUFA 0062, and it was tested on various activities (antibacterial activity, biofilm formation inhibition activity and cytotoxic activity). Candidusin D showed cytotoxicity to all cell lines tested (HepG2, HT-29, HCT-116, A549, A375, MCF-7, U251 and T98G) except for T98G and HepG2 at the concentration of 100 µM [116]. Diketopiperazine dimer (**284**) was isolated from fungus *Aspergillus violaceofuscus* and it showed anti-inflammatory activity against IL-10 expression of the LPS-induced THP-1 cells with an inhibitory rate of 78.1% at a concentration of 10 µM [117]. 3-Hydroxysperadine A (**285**) was isolated from HMP-F28 induced extracellular alkalinization and H_2_O_2_ production in tobacco cell suspensions by a bioassay-guided fractionation and purification, and no activity was reported [118].

**Figure 30:**
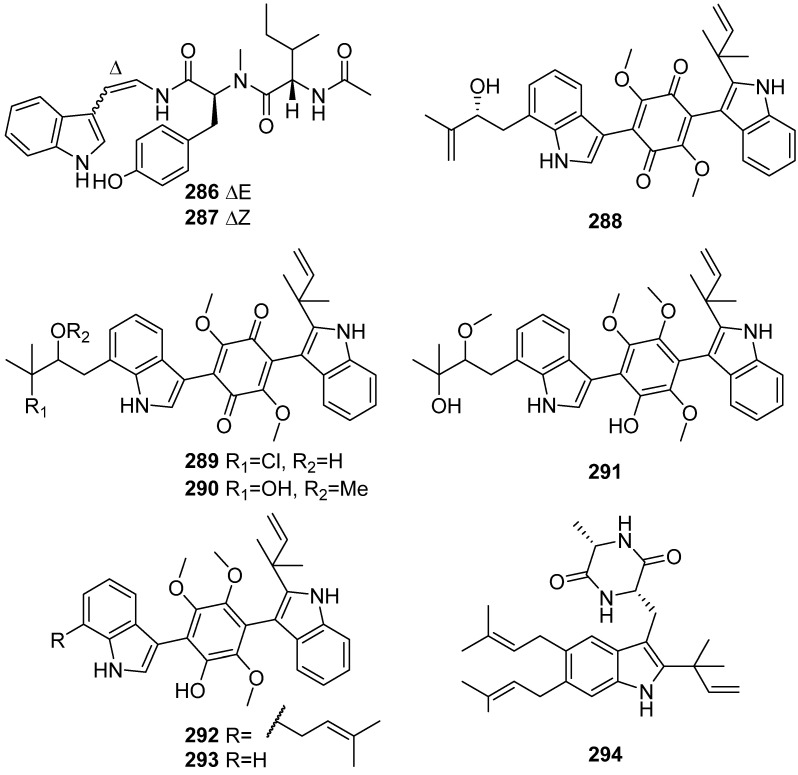
Chemical structures of **286**–**294**.

Aspergillamides C and D (**286** and **287**) were obtained from the marine sponge-derived fungus *Aspergillus terreus* SCSIO 41008 (Figure 30) [119]. Asterriquinones I–K (**288**–**290**), three new bis-indolylquinones, and asterriquinols G–I (**291**–**293**), three new bis-indolylbenzenoids, were isolated from the sponge-derived fungus *Aspergillus* sp. SCSIO 41,018. Asterriquinones I–K (**288**–**290**) displayed cytotoxic activities against K562, BEL-7042, SGC-7901, A549 and Hela cell lines [120]. A diketopiperazine–indole alkaloid fintiamin (**294**) was isolated from fungus *Eurotium* sp. It showed an affinity for the cannabinoid CB1 receptor at low micromolar concentrations [121].

#### 2.2.7. Miscellaneous

**Figure 31:**
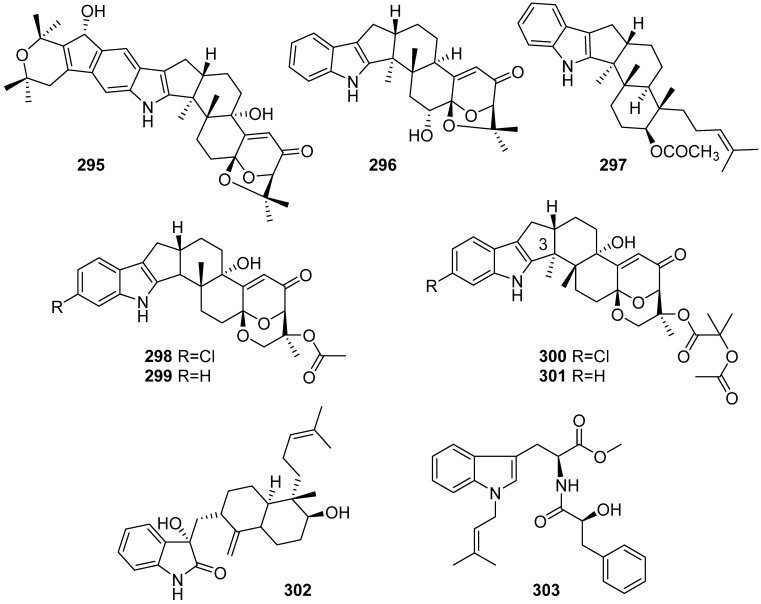
Chemical structures of **294**–**303**.

Three new indolediterpenoids, 22-hydroxylshearinine F (**295**), 6-hydroxylpaspalinine (**296**) and 7-O-acetylemindole SB (**297**), were isolated from the sea-anemone-derived fungus *Penicillium* sp. AS-79. Only **296** exhibited activity against the aquatic pathogen *Vibrio parahaemolyticus* with a MIC value of 64.0 µg/mL, which was much bigger than the positive control chloromycetin with a MIC value of 0.5 µg/mL (Figure 31) [122]. Four new indole-diterpene alkaloids, asperindoles A–D (**298**–**301**), were isolated from the marine-derived fungus *Aspergillus* sp., associated with an unidentified colonial ascidian. Asperindole A (**298**) exhibited cytotoxic activity against PC-3, LNCaP and 22Rv1 with IC_50_ values of 69.4 μM, 47.8 μM and 4.86 μM, and induced apoptosis in 22Rv1 at the concentration of 0.3125 μM. Furthermore, 22Rv1 cells treated with asperindole A (**298**) for 48 h revealed an S-phase arrest [123]. Penicindopene A (**302**), a new indole diterpene, was isolated from the deep-sea fungus *Penicillium* sp. YPCMAC1. Compound **302** represented the first example of indole diterpene possessing a 3-hydroxyl-2-indolone moiety, and it exhibited moderate cytotoxicities against A549 and HeLa cell lines with IC_50_ values of 15.2 and 20.5 μM, respectively [124]. Misszrtine A (**303**) was isolated from marine sponge-derived fungus *Aspergillus* sp. SCSIO XWS03F03. Compound **303** represents the first example of N-isopentenyl tryptophan methyl ester with a phenylpropanoic amide arm, which exhibited a potent antagonistic activity on HL60 (IC_50_ = 3.1 µM) and LNCaP (IC_50_ = 4.9 µM) cell lines [125]. 

**Figure 32:**
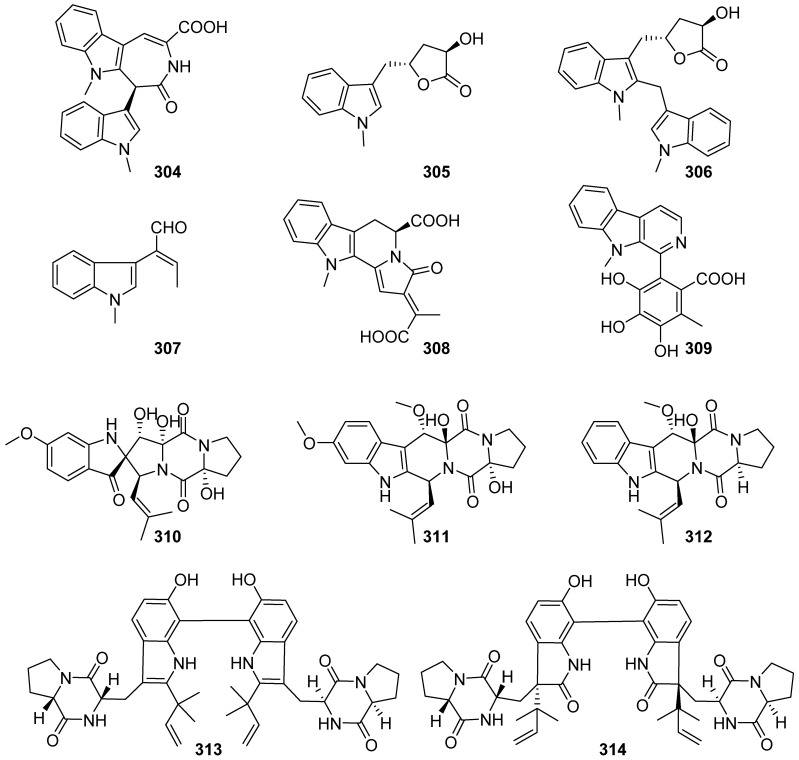
Chemical structures of **304**–**314**.

Chaetoindolones A–D (**304**–**307**), 19-O-desmethylchaetogline A (**308**) and 20-O-desmethylchaetogline F (**309**) were produced by the marine fish-derived fungus *Chaetomium globosum* 1C51 through biotransformation (Figure 32). Alkaloids **304**, **306**, **308**, and **309** showed antibacterial activities with MIC ranging from 8 to 128 μg/mL against *Xanthomonas oryzae pv. oryzae*, *Ralstonia solanacearum*, *Xanthomonas oryzae pv. oryzicola* and *Pseudomonas syringae pv. lachrymans*. Chaetoindolone A (**304**) was shown to inhibit the growth of the rice-pathogenic bacteria *Xanthomonas oryzae pv. oryzae* both in vitro and in vivo [126]. Three indole-diketopiperazines, spirotryprostatin G (**310**, an oxindole derivative), cyclotryprostatin F (**311**) and cyclotryprostatin G (**312**), were obtained by large-scale cultivation of the marine-derived fungus *Penicillium brasilianum* HBU-136 with the aid of genomic analysis. Compound **310** displayed selective cytotoxicities against the HL-60 cell line with an IC_50_ value of 6.0 μM, whereas compounds **311** and **312** exhibited activities against the MCF-7 cell line with the IC_50_ values of 7.6 and 10.8 μM, respectively. However, all of the metabolites appeared to be inactive in antibacterial and antifungal assays (MIC > 25 μM) [127]. Chemical investigation of secondary metabolites from the marine-derived fungus *Aspergillus austroafricanus* Y32-2 resulted in isolating two new prenylated indole alkaloid homodimers, di-6-hydroxydeoxybrevianamide E (**313**) and dinotoamide J (**314**). Each compound was evaluated for proangiogenic, anti-inflammatory effects in zebrafish model and cytotoxicity for HepG-2 human liver carcinoma cells. As a result, compound **314** exhibited proangiogenic activity in a PTK787-induced vascular injury zebrafish model in a dose-dependent manner [128].

**Figure 33:**
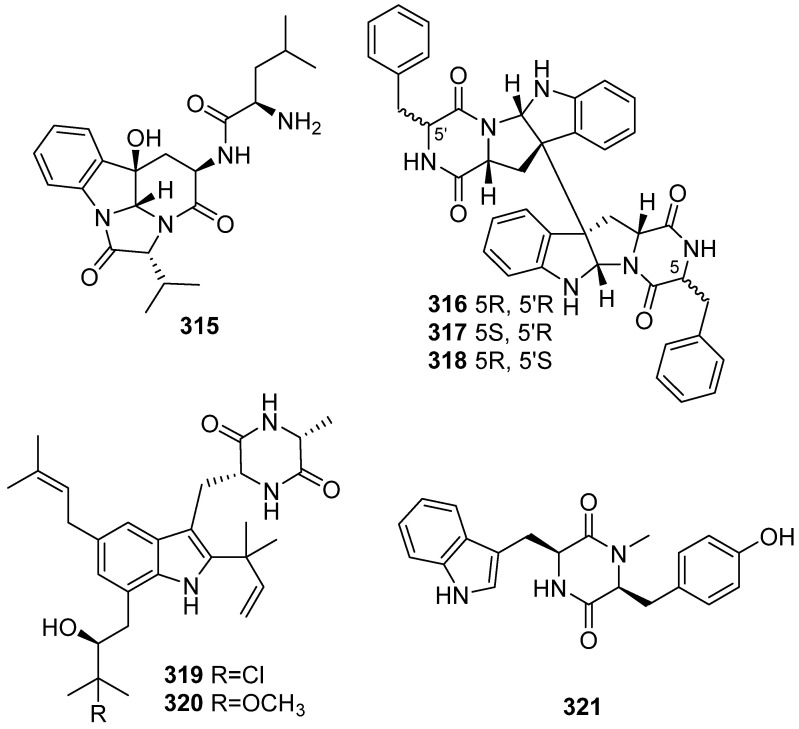
Chemical structures of **315**–**321**.

Asperginine (**315**), an alkaloid possessing a rare skeleton, was isolated from the cultural broth of the marine fungus *Aspergillus* sp., and it has no cytotoxicity against prostate cancer PC3 and human HCT-116 (Figure 33) [129]. Compounds **316**–**318** were isolated from the culture broth of a marine gut fungus *Aspergillus* sp. DX4H, and only showed weak inhibitory activity at 20 μg/mL against PC3 cell line [130]. Two new dioxopiperazine alkaloids (**319** and **320**) were isolated from Antarctic marine-derived *Aspergillus* sp. SF-5976. Compound **320** decreased PGE2 production in RAW 264.7 and BV2 cells, and **319** only showed inhibitory effects in BV2 cells and the same situation on LPS-stimulated NO production in RAW 264.7 and BV2 cells [131]. Quellenin (**321**) was isolated from deep-sea fungus *Aspergillus* sp. YK-76. It showed weak inhibition against the growth of *S. parasitica* with inhibition zones of 19.9 mm at the dosage of 200 μg/disc [132].

## 3. Marine Invertebrates

### 3.1. Sponges

Sponges are the simplest multicellular animals in the world, and they settle across the bottom of the sea with more than 10,000 species. There are 133 new indole related compounds isolated from invertebrates recently, and 109 are from 33 species of sponges. 

**Figure 34:**
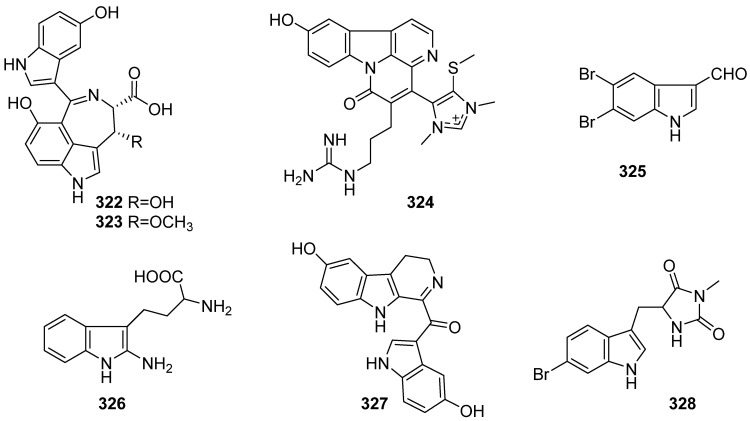
Chemical structures of **322**–**328**.

The marine sponge of the genus *Hyrtios* has been recognized as a rich source of unique bioactive products. In recent years, six references containing seven indole alkaloids have been reported, including hyrtinadines C (**322**) and D (**323**), ishigadine A (**324**), 5, 6-dibromoindole-3-carboxaldehyde (**325**), hyrtiodoline A (**326**), 3,4-dihydrohyrtiosulawesine (**327**) and bromoindole alkaloid (**328**) (Figure 34). Various activities were evaluated for these alkaloids. Compound **322** showed antifungal activity against *A. niger* (IC_50_ = 32 μg/mL), while **323** displayed antibacterial activity against *E. coli* (MIC = 16 μg/mL) and *B. subtilis* (MIC = 16 μg/mL). Compound **324** exhibited cytotoxicity against L1210 murine leukemia cells (IC_50_ = 3.3 μg/mL) in vitro. Compound **326** has the most potent antitrypanosomal activity, with an IC_50_ value of 7.48 μM after 72 h. Compound **327** displayed potent inhibitory activities against isocitrate lyase (IC_50_ = 92.9 µM) from *Candida albicans*. Compound **328** exhibited weak cytotoxicity against HCT-116, MCF-7 and HepG-2 (IC_50_ > 100 μM), and it also inhibits the growth of *S. aureus* and *E. coli* at the concentration of 3 mg/mL with the inhibition zones of 14 and 21 mm, respectively [133,134,135,136,137,138]. 

**Figure 35:**
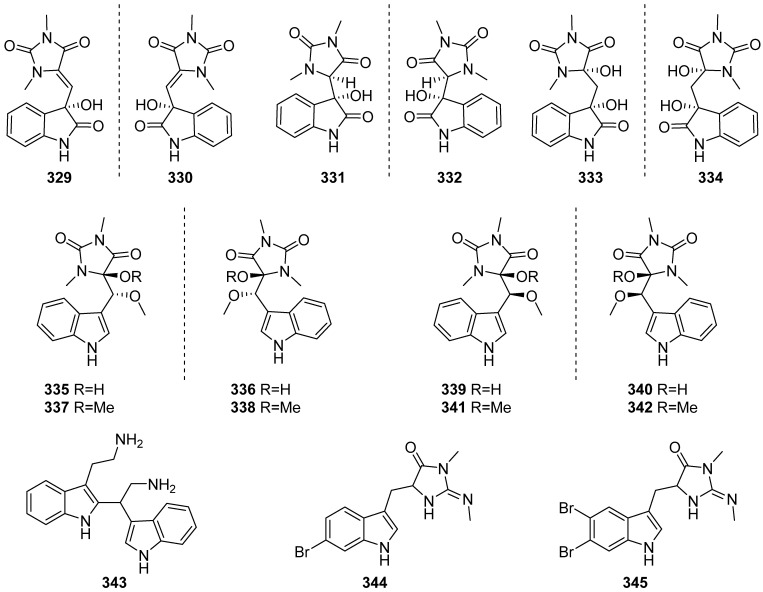
Chemical structures of **329**–**345**.

Seventeen indole alkaloids (**329**–**345**) were isolated from the sponge *Fascaplysinopsis reticulata* collected in Mayotte and Xisha Islands (Figure 35). Fourteen of them were new oxygenated aplysinopsin-type enantiomers, (+)- and (−)-oxoaplysinopsins A–G (**329**–**342**), and the others were 6,6′-bis-(debromo)-gelliusine F (**343**), 6-bromo-8,1′-dihydro-isoplysin A (**344**) and 5,6-dibromo-8,1′-dihydro-isoplysin A (**345**). Compounds **331** and **332** showed tyrosine phosphatase 1B inhibition activity stronger than the positive control of acarbose. Compounds **333** and **334** exhibited cytotoxicity against the Hela cell line. Compounds **344** and **345** displayed antimicrobial activities towards *Vibrio natrigens* with MIC values of 0.01 and 1 µg/mL, respectively [139,140]. 

**Figure 36:**
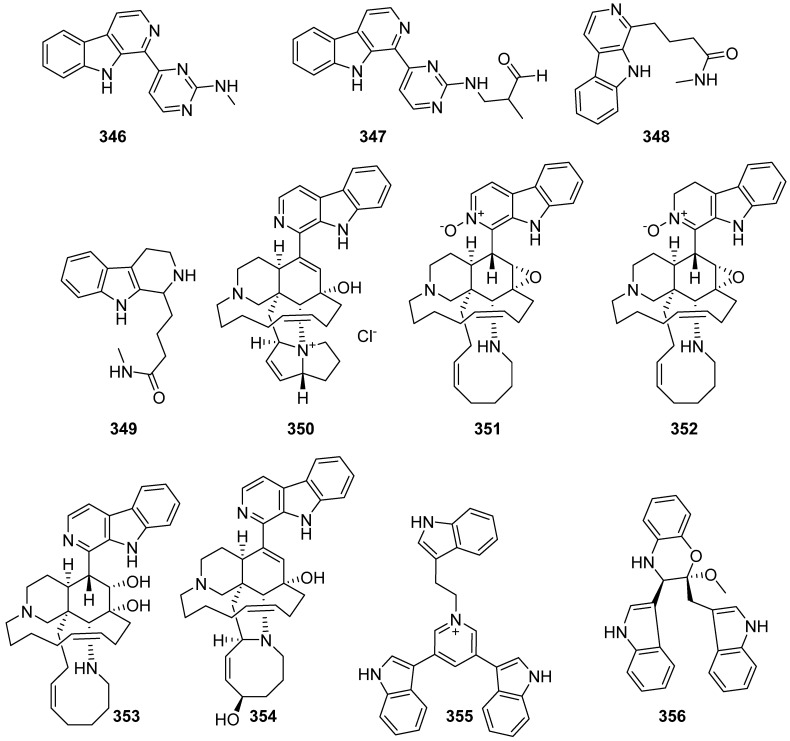
Chemical structures of **346**–**356**.

Sabrin R.M. Ibrahim group isolated ingenines C–F (**346**–**349**) from the Indonesian sponge *Acanthostrongylophora ingens* (Figure 36). Ingenine C (**346**) and D (**347**) were evaluated for their cytotoxic activity towards MCF-7, A549 and HCT-116 cell lines. It is noteworthy that compounds **346** and **347** exhibited cytotoxic activities against MCF-7 and HCT-116 with IC_50_ values of 4.33 and 6.05 and 2.90 and 3.35 μM, respectively. Ingenine E (**348**) exhibited cytotoxic activity against MCF-7, HCT-116 and A549 cell lines with IC_50_ values of 3.5, 0.67 and 2.15 μg/mL. Ingenine F (**349**) exhibited cytotoxic activity toward MCF-7, HCT-116 and A549 cell lines with IC_50_ values of 2.82, 1.00 and 2.37 μM, respectively [141,142,143]. Five new manzamine alkaloids (**350**–**354**) were isolated from an Indonesian *Acanthostrongylophora* sp. sponge. They exhibited weak cytotoxicity against A549 and K562 (LD_50_ = 4.6–12 μM), and moderate antibacterial activity to six bacteria (MIC > 1.6 ng/mL). Compounds **352**–**354** showed mild inhibition against the enzyme isocitrate lyase [144].

A novel pyridinium, tricepyridinium (**355**), and a novel benzoxazine–indole hybrid (**356**, racemic mixture) were obtained from the culture of an *E. coli* clone incorporating metagenomic libraries from the marine sponge *Discodermia calyx*. Compound **356** was speculated to be formed through a nonenzymatic process during the isolation procedure. The synthesized tricepyridinium bromide showed antimicrobial activity against *Bacillus cereus*, MSSA and *Candida albicans* with MIC values of 0.78, 1.56 and 12.5 μg/mL, but not against *E. coli*. In addition, tricepyridinium bromide had cytotoxicity to P388 cells with an IC_50_ value of 0.53 ± 0.07 μg/mL. Compound **356** exhibited no antibacterial activity against the tested *Bacillus cereus* [145,146]. 

**Figure 37:**
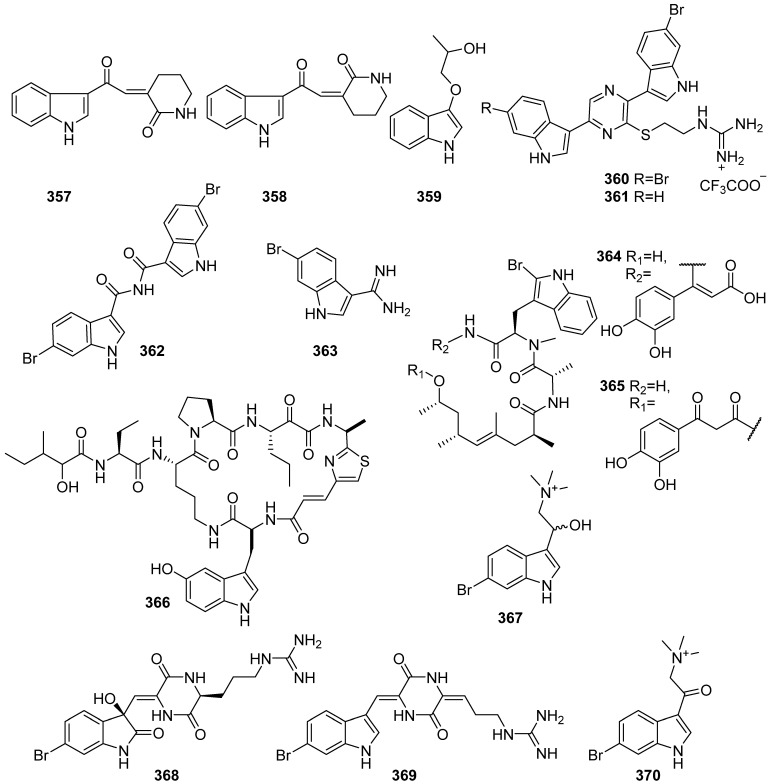
Chemical structures of **357**–**370**.

Two new indole alkaloids (**357** and **358**) were obtained from *Spongia* sp. collected by SCUBA in the South Sea of Korea (Figure 37). They did not display any significant inhibitory activity on farnesoid X-activated receptor (FXR) up to 100 μM, and they were not cytotoxic to CV-1 cells up to 200 μM on MTT assay, either [147]. 1-(1H-indol-3-yloxy) propan-2-ol (**359**) was isolated from the Red Sea sponges *Haliclona* sp. and showed weak cytotoxic activities against the tested HepG-2, Daoy and HeLa by MTT assay [148]. Two bisindole alkaloids tethered by a guanidino ethylthiopyrazine moiety, dragmacidins G (**360**) and H (**361**), were isolated from *Lipastrotethya* sp. marine sponge. Dragmacidin G (**360**), and dragmacidin H (**361**), showed cytotoxicity against HeLa cells with IC_50_ values of 4.2 and 4.6 μM, respectively [149]. Chemical investigation of a specimen of *Jaspis splendens* collected from the Great Barrier Reef resulted in the isolation of a new bisindole alkaloid, splendamide (**362**), and 6-bromo-1H-indole-3-carboximidamide (**363**) are reported for the first time as naturally occurring metabolites. They were subjected to an unbiased phenotypic assay on hONS cells as a model of Parkinson’s disease followed by cluster analysis of cytological effects and showed similar biological activity in cluster B. under a Pearson’s correlation of 0.91 [150]. A new acrylic jasplakinolide congener (**364**) and another structure-revised acyclic derivative (**365**) were isolated from the Indonesian marine sponge *Jaspis splendens*, and the jasplakinolides inhibited the growth of mouse lymphoma (L5178Y) cells in vitro with IC_50_ values in the low micromolar to the nanomolar range [151]. A new cyclic peptide, jamaicensamide A (**366**), composed of six amino acids, including thiazole-homologated amino acid, was isolated from the Bahamian sponge *Plakina jamaicensis* collected from Plana Cay, and no bioactivity have been evaluated due to the insufficient quantities [152]. A novel brominated marine indole (**367**) was isolated from the boreal sponge *Geodia barretti* collected off the Norwegian coast. Compound **367** was inactive (IC_50_ > 690 μM) on electric eel AChE even with a structural resemblance with other known natural AChE inhibitors and showed somewhat higher inhibitory potential towards BChE (IC_50_ = 222 μM) [153]. Geobarrettin A–C (**368**–**370**) were isolated from the sub-Arctic sponge *Geodia barrette* by UPLC-qTOF-MS-based dereplication study. Both **369** and **370** reduced DC secretion of IL-12p40, but **370** concomitantly increased IL-10 production. Maturing DCs treated with **369** or **370** before co-culturing with allogeneic CD4+ T cells decreased T cell secretion of IFN-γ, indicating a reduction in Th1 differentiation [154].

**Figure 38:**
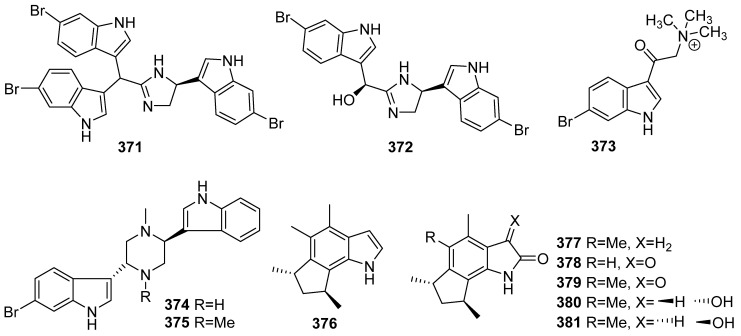
Chemical structures of **371**–**381**.

Antibacterial-guided fractionation of an extract from a deep-water *Topsentia* sp. marine sponge led to the isolation of two new indole alkaloids, tulongicin A (**371**) and dihydrospongotine C (**372**) (Figure 38). Antibacterial, anti-HIV activity and cytotoxicity were evaluated for compounds **371** and **372**. They showed strong antibacterial effects toward *S. aureus* with 1.2 and 3.7 μg/mL MICs. However, only weak to no inhibition toward *E. coli* at the maximum concentration tested (100 μg/mL) was reported. Both compounds inhibited HIV infection in HIV infectivity assays against the CCR5-tropic primary isolate YU2 and the CXCR4-tropic strain HxB2 with the IC_50_ values ranging from 2.7 to 4.5 μM. They were inactive (IC_50_ > 10 μM) in cytotoxicity assays against a monkey kidney cell line (BSC-1) and a human colorectal tumor cell line (HCT-116) [155]. A new brominated indole 6-Br-8-keto-conicamin A (**373**) was identified from *Haplosclerida* sponge, and it showed moderate cytotoxic activity against the PANC-1 tumor cell line with the IC_50_ value of 1.5 μM [156]. Two new brominated bisindole alkaloids, dragmacidins I (**374**) and J (**375**), were isolated from the Tanzanian sponge *Dragmacidon* sp. They showed low micromolar cytostatic activity against A549, HT-29 and MDA-MB-231. The mechanism of the action was investigated through different molecular biology experiments, which indicated that these two dragmacidins act via the inhibition of Ser-Thr PPs [157]. Six new cyclopenta[*g*]indole natural products, trans-herbindole A (**376**) and trikentramides E–I (**377**–**381**), were isolated from the sponge *Trikentrion flabelliforme*, and there is no bioactivity reported [158].

**Figure 39:**
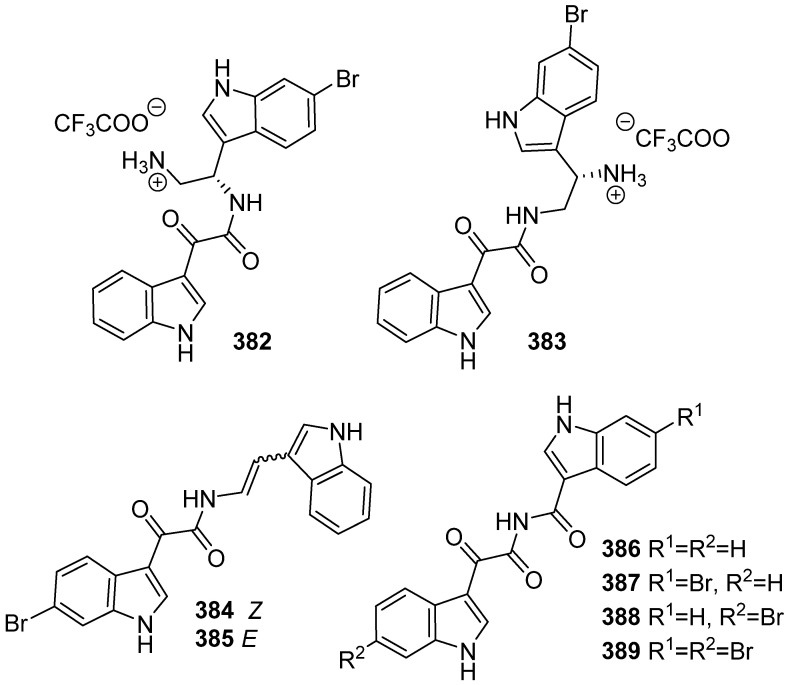
Chemical structures of **382**–**389**.

The first chemical investigation of the subtidal sponge *Spongosorites calcicola* led to the discovery of two new bisindole alkaloids of the topsentin family (**382** and **383**), and they showed very weak or no cytotoxic activity against the Hela cell line (Figure 39) [159]. Six new bisindoles, (Z)-coscinamide D (**384**), (E)-coscinamide D (**385**) and lamellomorphamides A–D (**386**–**389**) were isolated from a rare New Zealand deep-sea sponge, *Lamellomorpha strongylata*. Compounds **385**, **386**, **389** showed weak activity against MRSA at the concentration of 20 μM (14.9–18.2% inhibition) [160]. 

**Figure 40:**
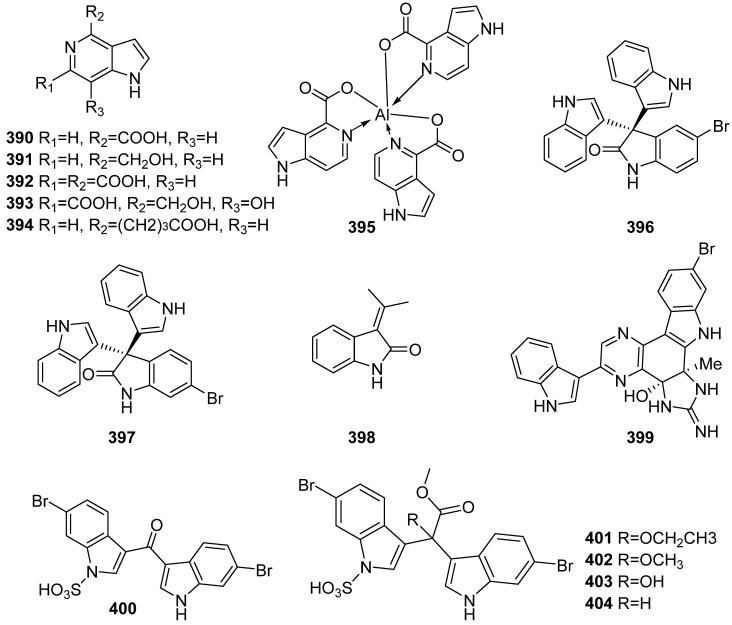
Chemical structures of **390**–**404**.

Guitarrins A–E (**390**–**394**), the first natural 5-azaindoles, and aluminumguitarrin A (**395**), the first aluminum-containing compound from marine invertebrates, were isolated from the sponge *Guitarra fimbriata* (Figure 40). Guitarrin C (**392**) inhibited alkaline phosphatase from the marine bacterium *Cobetia marina* with an IC_50_ value of 2.0 µM, being a natural inhibitor of alkaline phosphatase [161]. Two brominated oxindole alkaloids (**396** and **397**) were isolated from sponge *Callyspongia siphonella* with LC-HRESIMS-assisted dereplication and bioactivity-guided isolation. The sponge was collected from Hurghada along the Red Sea Coast. Oxindoles **396** and **397** exhibited diverse pharmacological activities, including antibacterial activity, biofilm inhibitory activity, antitrypanosomal activity and antitumor activity. They inhibited the growth of *Staphylococcus aureus* (MIC = 8 and 4 µg/mL), *Bacillus subtilis* (MIC = 16 and 4 µg/mL), *Pseudomonas aeruginosa* (49.32% and 41.76% inhibition at the concentration of 128 µg/mL), and *T. brucei* (13.47 and 10.27 µM for 72 h). In addition, they showed good cytotoxic effect toward HT-29, OVCAR-3 and MM.1S with IC_50_ values ranging from 7 to 12 µM through non-programmed cell necrosis [162]. A naturally new alkaloid (**398**) was isolated from *Gelliodes* sp. collected in Vietnam, and showed no cytotoxicity against Hela, MCF-7 and A549 cell lines [163]. Myrindole A (**399**), a bis-indole alkaloid, was isolated from the deep-sea sponge *Myrmekioderma* sp. Myrindole A inhibits the growth of *E. coli* and *Bacillus subtilis* with MIC values of 37.5 and 18.5 μM, respectively [164]. The structures of a series of incorrectly reported sponge-derived dibrominated indole alkaloids, echinosulfone A (**400**) and the echinosulfonic acids A–D (**401**–**404**) were corrected [165,166,167]. Another two papers have also disclosed identical structure revisions for these dibrominated indole alkaloids (**400**–**404**) [168,169].

**Figure 41:**
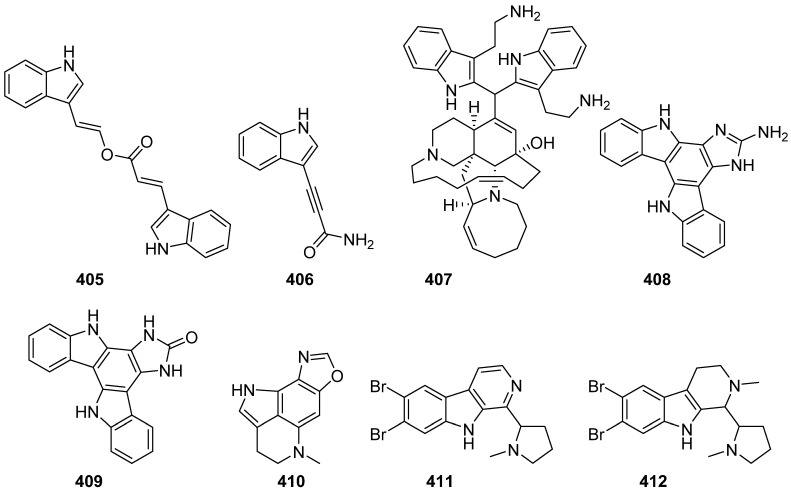
Chemical structures of **405**–**412**.

A bis-indole (**405**) and an alkynyl indole alkaloid (**406**) were isolated from the sponge *Plakortis* sp. collected from Zampa in Okinawa (Figure 41). The bis-indole was inactive against both P388 and B16 cells even at 100 µg/mL, while **406** showed cytotoxicity against P388 at 1 µg/mL (IC_50_ = 0.6 µg/mL) and B16 cells at 100 µg/mL [170]. Zamamidine D (**407**) was isolated from an Okinawan *Amphimedon* sp. marine sponge, and it exhibited obvious antibacterial activity against the eight tested strains (*Escherichia coli*, *Stapylococcus aureus*, *Micrococcus luteus*, *Aspergillus niger*, *Trichophyton mentagrophytes*, *Candida albicans* and *Cryptococcus neoformans*) with IC_50_ values ranging from 2 to 32 µg/mL [171]. An extract of the marine sponge *Damiria* sp., which represents an understudied genus, provided two novel alkaloids named damirines A (**408**) and B (**409**). Compound **408** showed selective cytotoxic properties toward six different cell lines in the NCI-60 cancer screen [172]. Makaluvamine W (**410**) was isolated from the Tongan sponge *Strongylodesma tongaensis*. Compound **410** was inactive to the tested HL-60 cell line and confirmed the requirement of an intact iminoquinone functionality required by these metabolites to be bioactive [173]. In the study of developing a metric-based prioritization approach by exact LC-HRMS, **411** and **412** were isolated in a case study from a sponge collected from a reef on the island of Tavarua, Fiji Islands. No activity was evaluated for **411** and **412** [174].

**Figure 42:**
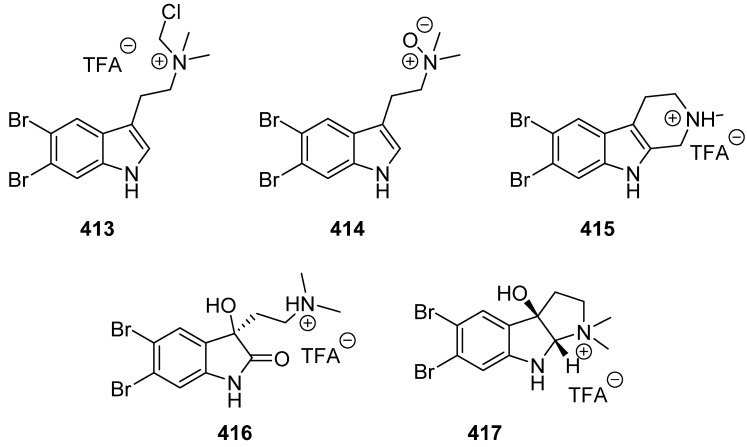
Chemical structures of **413**–**417**.

Five dibromoindole alkaloids (**413**–**417**) were isolated from sponge *Narrabeena nigra* collected around the Futuna Islands (Figure 42). They reduced the TBHP-induced cell death, which demonstrated their potential in neuroprotection, and showed almost no cytotoxic effect up to 10 µM on human neuroblastoma SH-SY5Y and microglia BV2 cells [175].

**Figure 43:**
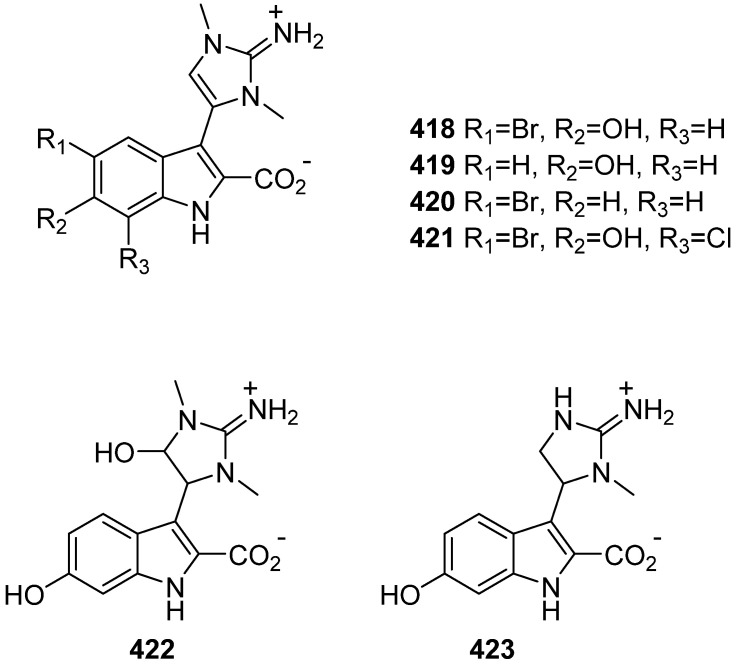
Chemical structures of **418**–**423**.

New indolo-imidazole alkaloids trachycladindoles H–M (**418**–**423**) were isolated from a deep-water Great Australian Bight sponge, *Geodia* sp. CMB-01063 (Figure 43). The trachycladindoles H–M did not exhibit growth inhibitory activity against the *E. coli*, *Bacillus subtilis*, *Candida albicans* human colorectal (SW620) or lung (NCI-H460) carcinoma cells [176].

**Figure 44:**
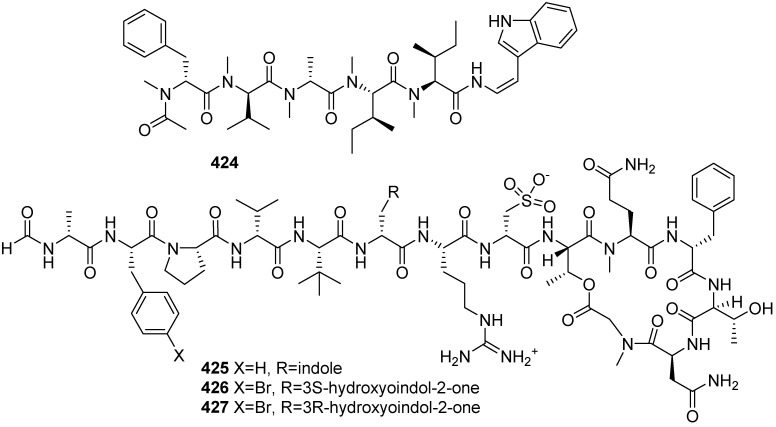
Chemical structures of **424**–**427**.

A highly modified hexapeptide friomaramide (**424**) was isolated from the Antarctic sponge *Inflatella coelosphaeroides*, and it blocks more than 90% of *Plasmodium falciparum* liver-stage parasite development at 6.1 μM (Figure 44) [177]. Halicylindramides F–H (**425**–**427**) were isolated from a *Petrosia* sp. marine sponge collected off the shore of Youngdeok-Gun, East Sea, Republic of Korea. Halicylindramides F (**425**) showed human farnesoid X receptor (hFXR) antagonistic activity, but it did not bind directly to hFXR [178].

**Figure 45:**
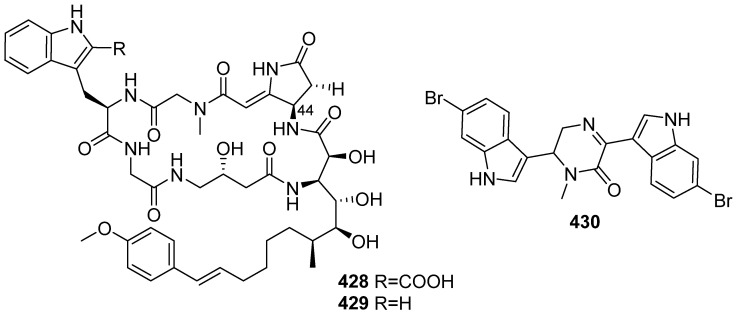
Chemical structures of **428**–**430**.

Microsclerodermins B and J (**428** and **429**) were reported by Faulkner and co-workers and Li and co-workers from marine sponge *Microscleroderma* (Figure 45) [179,180]. The configuration of the C44 stereocenter of microsclerodermin B was confirmed by total synthesis of dehydromicosclerodermin B, which was revised from *44S* to *44R.* The same method was applied with microsclerodermins J, and the configuration was revised to be *44R* [181]. The structure of topsentin C (**430**) was revised by an efficient total synthesis [182].

### 3.2. Cnidarians

**Figure 46:**
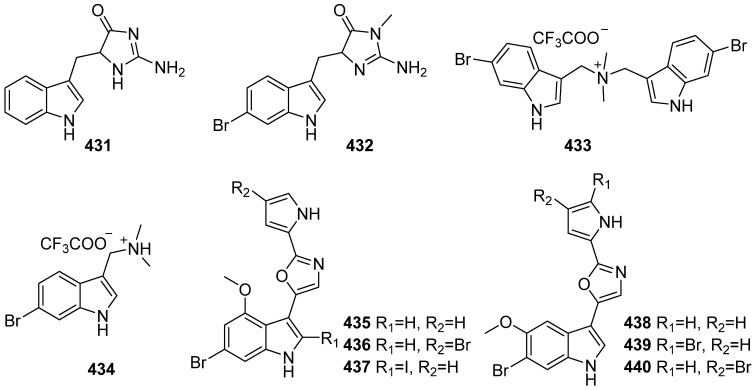
Chemical structures of **431**–**440**.

Two new indole alkaloids 2-amino-1,5-dihydro-5-(1H-indol-3-ylmethyl)-4H-imidazol-4-one (**431**) and 2-amino-5-[(6-bromo-1H-indol-3-yl)methyl]-3,5-dihydro-3-methyl-4H-imidazol-4-one (**432**) were isolated from the sea anemone *Heteractis aurora*, and no activity was reported (Figure 46) [183]. Two new natural products, bis-6-bromogramine (**433**) and 6-bromogramine (**434**), were isolated from the marine hydroid *Abietinaria abietina*. They activate NF-κB-dependent transcriptional activity in JB6 Cl 41 NF-κB cells at 1.6 µM [184]. A series of new indole-oxazole-pyrrole natural products breitfussins C–H (**438**, **435**–**437**, **439**, **440**), along with breitfussin A and B, were isolated from marine hydrozoan *Thuiaria breitfussi*. The hydrozoan was collected from Bjørnøya, Svalbard (79.0293 N, 20.8574 E, at 48 m depth). Cytotoxic activity and kinase inhibition profiles of breitfussins C–F were evaluated against seven malignant cell lines, one non-malignant cell line and 13 kinases. Then, the pharmacological potential of breitfussin C was extended to the activities evaluation of 88 cancer cell lines and 468 kinases. The results showed excellent inhibition and selectivity against MDA-MB-468 with the IC_50_ value of 340 nM and against the PIM1 and DRAK1 kinases with IC_50_ and Kd values of 200 and 390 nM, respectively. Further studies on potential off-target effects, toxicological effects, as well as relevant in vitro ADME, displayed the potential of breitfussins for selective kinase inhibitor development [185]. 

### 3.3. Bryozoans, Tunicates and Molluscs

**Figure 47:**
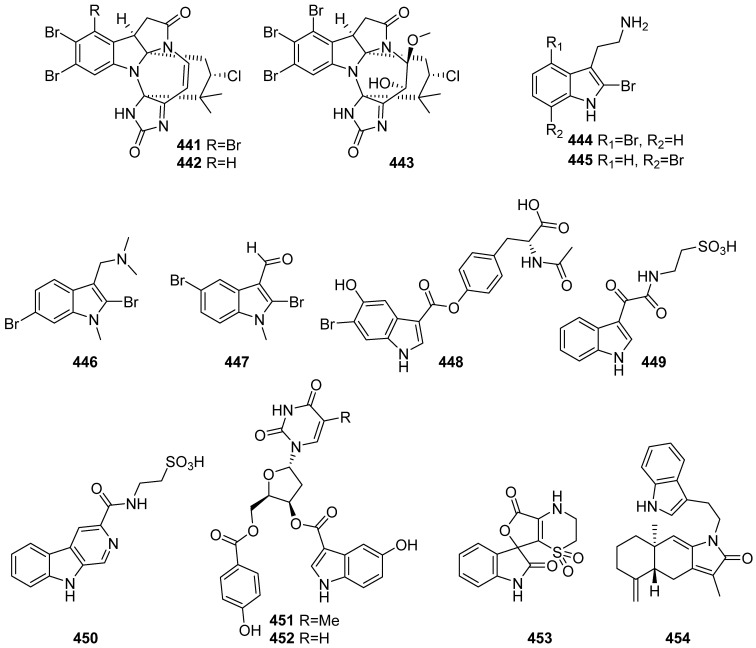
Chemical structures of **441**–**454**.

Three new halogenated, hexacyclic indoleline-imidazole alkaloids, securamines H–J (**441**–**443**), were isolated from Arctic bryozoan *Securiflustra securifrons* (Figure 47). Compounds **441** and **442** showed cytotoxicity against the human cancer cell lines A2058 (skin), HT-29 (colon), and MCF-7 (breast), as well as against nonmalignant human MRC-5 lung fibroblasts with IC_50_ values ranging from 1.4 ± 0.1 to 5.3 ± 1.1 μM. The cytotoxicity of **441** was further evaluated and found to be time-dependent [186]. A new compound, 2,6-dibromo-N-methylgramine (**446**), was discovered in bryozoan *Amathia verticillata*, and no activity was reported [187]. 2,5-dibromo-1-methyl-1H-indole-3-carbaldehyde (**447**) was isolated from the bryozoan *A. lamourouxi*, which was collected from rock pools of Woolgoolga, Australia. Compound **447** was inactive for the tested antiplasmodial activity and HEK293 cytotoxicity at 40 μM [188]. Terminoflustrindoles (TFIns) B and C (**444** and **445**) were isolated from the bryozoan *Terminoflustra membranaceatruncata*, and they exhibit no antimicrobial activity against various microorganisms tested [189]. A new indole alkaloid **448** was isolated from a colonial marine tunicate, *Didemnum* sp., collected by SCUBA near Haegeumgang, Korea. Compound **448** showed no pharmacological potential as an antibacterial agent and FXR antagonist [190]. Stolonines A and C (**449** and **450**) were isolated from a marine tunicate *Cnemidocarpa stolonifera*. An immunofluorescence assay on PC3 cells indicated that compounds **449** and **450** increased cell size, induced mitochondrial texture elongation, and caused apoptosis in PC3 cells [191]. Compounds **451** and **452** were reported in a case study of the metrics-based prioritization approach development. They were isolated from the pink mottled tunicate collected from Tavarua, Fiji Islands [174]. Orbicularisine (**453**), a novel spiro-indolofuranone fused to a thiazine skeleton, was isolated from gills of the mollusk *Codakia orbicularis*. Compound **453** was inactive against *Enterococcus faecalis*, *Streptococcus pneumonia*, *Klebsiella pneumonia*, *E. coli*, and *Pseudomonas aeruginosa*. Inhibition assays against a panel of kinases including Hs_CDK2/CyclinA, Hs_CDK5/p25, Hs_CDK9/CyclinT, Hs_RIPK3, Hs_Haspin, Hs_AuroraB, Ld_TLK, Hs-Pim1, Ssc_GSK3 a/b, Lm_CK1, and Rn_Dyrk1A showed residual activities of more than 60% for 16 μM/mL. Finally, the treatment of HCT-116 colon cancer cells and U87-MG glioblastoma cancer cells with concentrations up to 100 μM showed no activity [192]. Cespilamide E (**454**) was isolated from the Taiwanese soft coral *Cespitularia taeniata*, and it exhibited cytotoxicity against MCF-7, Daoy and Hela cancer cells with IC_50_ of 17.5, 22.3, and 24.7 μM, respectively [193].

## 4. Marine Plants

### 4.1. Cyanobacteria

**Figure 48:**
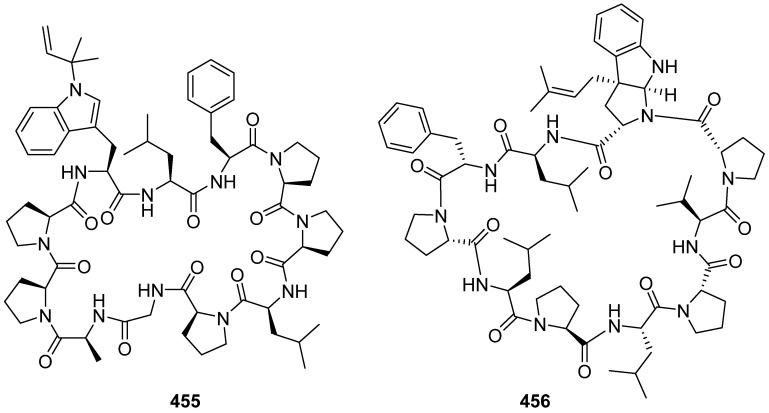
Chemical structures of **455**–**456**.

Two proline-rich cyclic peptides (**455** and **456**) were isolated from marine cyanobacterium *Symploca* sp., collected from Minna Island, Japan and Bintan Island, Indonesia (Figure 48). Compound **456** possessed cytotoxicity against the MOLT4 and AML2 cancer cell lines with IC_50_ values of 4.8 and 8.2 μM, respectively [194,195].

### 4.2. Red Algae

**Figure 49:**
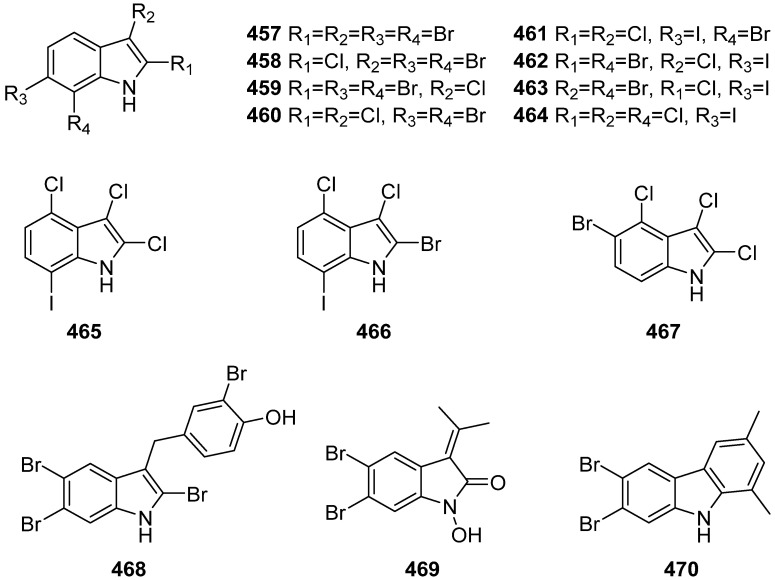
Chemical structures of **457**–**470**.

Eleven new tetrahalogenated indoles (**457**–**467**) were isolated from the red alga *Rhodophyllis membranacea*, collected from Moa Point, New Zealand (Figure 49). Compounds **457** and **459**–**461** showed no antifungal activity against wild-type *Saccharomyces cerevisiae* (baker’s yeast) and cytotoxicity against HL-60 promyelocytic leukemia cell line with IC_50_ values higher than 10 μM [196]. Compounds **468**–**470** were isolated and identified from the red algae *Laurencia similis*, and **468** showed potent antibacterial activity against seven bacterial strains with MIC values ranging from 2 to 8 μg/mL [197]. 

### 4.3. Mangrove

**Figure 50:**
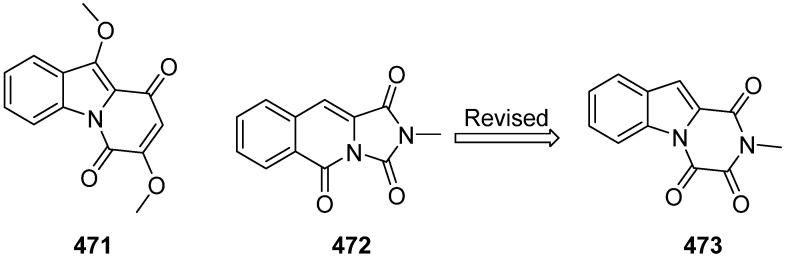
Chemical structure of **471**–**473**.

Chemical investigation of the leaves and stems of the Chinese mangrove *Acanthus ilicifolius* Linn. led to the isolation and structure elucidation of one new pyrido[1,2-a]indole alkaloid named acanthiline A (**471**), and no bioactivity was reported (Figure 50) [198]. Marine alkaloid **472** was reported as 2-methylimidazo [1,5-b]isoquinoline-1,3,5(2H)-trione, and it was revised to be **473** by a total synthesis [199,200].

## 5. Conclusions

In this article, we reviewed 472 indole alkaloids discovered from marine organisms with a vast of bioactivities during the year from 2015 to 2021. The alkaloids were grouped according to the sources, divided into marine microbes, invertebrates, and plants. Marine microbes are the main source of natural marine products, which is the case for indole alkaloids. A total of 321 new indole metabolites were isolated from marine microorganisms, including 64 from marine-sourced bacteria and 257 from marine-sourced fungi. Sponges have been abundant and stable sources of marine natural products over the years. Among the indole alkaloids discovered from marine invertebrates, sponge-derived make up the vast majority, with 109 out of 133 in total. Marine plants only contributed 18 indole compounds isolated from cyanobacteria, red algae and mangrove.

Due to the insufficient amount of compounds isolated, the bioactivity determination of natural products has always been a significant challenge. Although a number of top chemists are devoted to moving natural products synthesis from the laboratory to the factory, the total synthesis of some complex marine natural products remains a challenge, and not to mention industrialization. However, marine microbial fermentation has the characteristics of non-destruction of ecological resources, relatively low cost, and good reproducibility. Mass fermentation assisted by genetic engineering transformation can better realize industrial production. In addition, there are many kinds of marine microorganisms, which are inexhaustible sources for marine drugs development. At present, most marine indole alkaloids were evaluated for their antitumor and antimicrobial activities. This is not only because this class of compounds is more likely to exhibit such activity, but also because antitumor and antimicrobial activity measurements are generally easier to perform. It can be summarized from this review that marine indole alkaloids have rich skeleton structures and various pharmacological activities. How to transfer the chemical diversity to pharmacological activity diversity is another challenge. Therefore, it is expected that a more general and practical pharmacological activity assay should be developed and applied to the early drug development process.

Compounds with indole moiety typically have significant pharmacological activity. We hope that this review will promote the development of marine indole alkaloids in medicinal chemistry and pharmacology in terms of the extent of drug discovery.

## Data Availability

Not applicable.

## Supplementary Materials

The following are available online at https://www.mdpi.com/article/10.3390/md19120658/s1, Table S1: General information of the cell lines; Table S2: Summary of the marine indole alkaloids isolated from marine microorganisms; Table S3: Summary of the marine indole alkaloids isolated from marine invertebrates and plants.

## References

[1] Ame P., Clw S., Burke M.D. (2017). The natural productome. Proc. Natl. Acad. Sci. USA.

[2] Pye C.R., Bertin M.J., Lokey R.S., Gerwick W.H., Linington R.G. (2017). Retrospective analysis of natural products provides insights for future discovery trends. Proc. Natl. Acad. Sci. USA.

[3] Rodrigues T., Reker D., Schneider P., Schneider G. (2016). Counting on natural products for drug design. Nat. Chem..

[4] Newman D.J., Cragg G.M. (2016). Natural Products as Sources of New Drugs from 1981 to 2014. J. Nat. Prod..

[5] Smith T.E., Pond C.D., Pierce E., Harmer Z.P., Kwan J., Zachariah M.M., Harper M.K., Wyche T.P., Matainaho T.K., Bugni T.S. (2018). Accessing chemical diversity from the uncultivated symbionts of small marine animals. Nat. Chem. Biol..

[6] Abdelmohsen U.R., Balasubramanian S., Oelschlaeger T.A., Grkovic T., Pham N.B., Quinn R.J., Hentschel U. (2016). Potential of marine natural products against drug-resistant fungal, viral, and parasitic infections. Lancet Infect. Dis..

[7] Zhu Y., Zhao J., Luo L., Gao Y., Bao H., Li P., Zhang H. (2021). Research progress of indole compounds with potential antidiabetic activity. Eur. J. Med. Chem..

[8] Chauhan M., Saxena A., Saha B. (2021). An insight in anti-malarial potential of indole scaffold: A review. Eur. J. Med. Chem..

[9] Kochanowska-Karamyan A.J., Hamann M.T. (2010). Marine Indole Alkaloids: Potential New Drug Leads for the Control of Depression and Anxiety. Chem. Rev..

[10] Han Y., Dong W., Guo Q., Li X., Huang L. (2020). The importance of indole and azaindole scaffold in the development of antitumor agents. Eur. J. Med. Chem..

[11] Jia Y., Wen X., Gong Y., Wang X. (2020). Current scenario of indole derivatives with potential anti-drug-resistant cancer activity. Eur. J. Med. Chem..

[12] Fiore A., Murray P.J. (2021). Tryptophan and indole metabolism in immune regulation. Curr. Opin. Immunol..

[13] Netz N., Opatz T. (2015). Marine Indole Alkaloids. Mar. Drugs.

[14] Xiong Z.Q., Liu Q.X., Pan Z.L., Zhao N., Feng Z.X., Wang Y. (2015). Diversity and bioprospecting of culturable actinomycetes from marine sediment of the Yellow Sea, China. Arch. Microbiol..

[15] Anjum K., Kaleem S., Yi W., Zheng G., Lian X., Zhang Z. (2019). Novel Antimicrobial Indolepyrazines A and B from the Marine-Associated *Acinetobacter* sp. ZZ1275. Mar. Drugs.

[16] Yi W., Li Q., Song T., Chen L., Li X.-C., Zhang Z., Lian X.-Y. (2019). Isolation, structure elucidation, and antibacterial evaluation of the metabolites produced by the marine-sourced *Streptomyces* sp. ZZ820. Tetrahedron.

[17] da Silva A.B., Pinto F.C.L., Silveira E.R., Costa-Lotufo L.V., Costa W.S., Ayala A.P., Canuto K.M., Barros A.B., Araújo A.J., Marinho Filho J.D.B. (2019). 4-Hydroxy-pyran-2-one and 3-hydroxy-N-methyl-2-oxindole derivatives of *Salinispora arenicola* from Brazilian marine sediments. Fitoterapia.

[18] Song Y., Yang J., Yu J., Li J., Yuan J., Wong N.K., Ju J. (2020). Chlorinated bis-indole alkaloids from deep-sea derived *Streptomyces* sp. SCSIO 11791 with antibacterial and cytotoxic activities. J. Antibiot..

[19] Wang J.N., Zhang H.J., Li J.Q., Ding W.J., Ma Z.J. (2018). Bioactive Indolocarbazoles from the Marine-Derived *Streptomyces* sp. DT-A61. J. Nat. Prod..

[20] Zhou B., Qin L.-L., Ding W.-J., Ma Z.-J. (2018). Cytotoxic indolocarbazoles alkaloids from the *Streptomyces* sp. A65. Tetrahedron.

[21] Qin L.-L., Zhou B., Ding W., Ma Z. (2018). Bioactive metabolites from marine-derived *Streptomyces* sp. A68 and its Rifampicin resistant mutant strain R-M1. Phytochem. Lett..

[22] Cheng X., Zhou B., Liu H., Huo C., Ding W. (2018). One new indolocarbazole alkaloid from the *Streptomyces* sp. A22. Nat. Prod. Res..

[23] Davies-Bolorunduro O.F., Adeleye I.A., Akinleye M.O., Wang P.G. (2019). Anticancer potential of metabolic compounds from marine actinomycetes isolated from Lagos Lagoon sediment. J. Pharm. Anal..

[24] Wang C., Monger A., Wang L., Fu P., Piyachaturawat P., Chairoungdua A., Zhu W. (2018). Precursor-Directed Generation of Indolocarbazoles with Topoisomerase IIα Inhibitory Activity. Mar. Drugs.

[25] Yan X., Tang X.X., Qin D., Yi Z.W., Fang M.J., Wu Z., Qiu Y.K. (2016). Biosynthetic Functional Gene Analysis of Bis-Indole Metabolites from 25D7, a Clone Derived from a Deep-Sea Sediment Metagenomic Library. Mar. Drugs.

[26] Thi Q.V., Tran V.H., Mai H.D., Le C.V., Hong Mle T., Murphy B.T., Chau V.M., Pham V.C. (2016). Secondary Metabolites from an Actinomycete from Vietnam’s East Sea. Nat. Prod. Commun..

[27] Lorig-Roach N., Still P.C., Coppage D., Compton J.E., Crews M.S., Navarro G., Tenney K., Crews P. (2017). Evaluating Nitrogen-Containing Biosynthetic Products Produced by Saltwater Culturing of Several California Littoral Zone Gram-Negative Bacteria. J. Nat. Prod..

[28] Paulus C., Rebets Y., Tokovenko B., Nadmid S., Terekhova L.P., Myronovskyi M., Zotchev S.B., Rückert C., Braig S., Zahler S. (2017). New natural products identified by combined genomics-metabolomics profiling of marine *Streptomyces* sp. MP131-18. Sci. Rep..

[29] Reynolds K.A., Luhavaya H., Li J., Dahesh S., Nizet V., Yamanaka K., Moore B.S. (2018). Isolation and structure elucidation of lipopeptide antibiotic taromycin B from the activated taromycin biosynthetic gene cluster. J. Antibiot..

[30] Chen L.-Y., Wang X.-Q., Wang Y.-M., Geng X., Xu X.-N., Su C., Yang Y.-L., Tang Y.-J., Bai F.-W., Zhao X.-Q. (2018). Genome mining of Streptomyces xinghaiensis NRRL B-24674^T^ for the discovery of the gene cluster involved in anticomplement activities and detection of novel xiamycin analogs. Appl. Microbiol. Biotechnol..

[31] Zhang F., Braun D.R., Rajski S.R., DeMaria D., Bugni T.S. (2019). Enhypyrazinones A and B, Pyrazinone Natural Products from a Marine-Derived Myxobacterium *Enhygromyxa* sp.. Mar. Drugs.

[32] Li J.L., Chen D., Huang L., Ni M., Zhao Y., Fan H., Bao X. (2017). Antichlamydial Dimeric Indole Derivatives from Marine Actinomycete Rubrobacter radiotolerans. Planta Med..

[33] Elsayed Y., Refaat J., Abdelmohsen U.R., Ahmed S., Fouad M.A. (2019). Retraction Note to: Rhodozepinone, a new antitrypanosomal azepino-diindole alkaloid from the marine sponge-derived bacterium *Rhodococcus* sp. UA13. Med. Chem. Res..

[34] Che Q., Qiao L., Han X., Liu Y., Wang W., Gu Q., Zhu T., Li D. (2018). Anthranosides A-C, Anthranilate Derivatives from a Sponge-Derived *Streptomyces* sp. CMN-62. Org. Lett..

[35] Kikuchi S., Okada K., Cho Y., Yoshida S., Kwon E., Yotsu-Yamashita M., Konoki K. (2018). Isolation and structure determination of lysiformine from bacteria associated with marine sponge *Halichondria okadai*. Tetrahedron.

[36] El-Hawary S.S., Sayed A.M., Mohammed R., Khanfar M.A., Rateb M.E., Mohammed T.A., Hajjar D., Hassan H.M., Gulder T.A.M., Abdelmohsen U.R. (2018). New Pim-1 Kinase Inhibitor from the Co-culture of Two Sponge-Associated Actinomycetes. Front. Chem..

[37] Kim M.C., Cullum R., Machado H., Smith A.J., Yang I., Rodvold J.J., Fenical W. (2019). Photopiperazines A–D, Photosensitive Interconverting Diketopiperazines with Significant and Selective Activity against U87 Glioblastoma Cells, from a Rare, Marine-Derived Actinomycete of the Family Streptomycetaceae. J. Nat. Prod..

[38] Liu T., Jin J., Yang X., Song J., Yu J., Geng T., Zhang Z., Ma X., Wang G., Xiao H. (2019). Discovery of a Phenylamine-Incorporated Angucyclinone from Marine *Streptomyces* sp. PKU-MA00218 and Generation of Derivatives with Phenylamine Analogues. Org. Lett..

[39] Baunach M., Ding L., Willing K., Hertweck C. (2015). Bacterial Synthesis of Unusual Sulfonamide and Sulfone Antibiotics by Flavoenzyme-Mediated Sulfur Dioxide Capture. Angew. Chem..

[40] Nair V., Schuhmann I., Anke H., Kelter G., Fiebig H.H., Helmke E., Laatsch H. (2016). Marine Bacteria, XLVII—Psychrotolerant Bacteria from Extreme Antarctic Habitats as Producers of Rare Bis- and Trisindole Alkaloids. Planta Med..

[41] Ding L., He S., Wu W., Jin H., Zhu P., Zhang J., Wang T., Yuan Y., Yan X. (2015). Discovery and Structure-Based Optimization of 6-Bromotryptamine Derivatives as Potential 5-HT2A Receptor Antagonists. Molecules.

[42] Xie C.L., Xia J.M., Su R.Q., Li J., Liu Y., Yang X.W., Yang Q. (2018). Bacilsubteramide A, a new indole alkaloid, from the deep-sea-derived *Bacillus subterraneus* 11593. Nat. Prod. Res..

[43] Rodrigues A.M.S., Rohée C., Fabre T., Batailler N., Sautel F., Carletti I., Nogues S., Suzuki M.T., Stien D. (2017). Cytotoxic indole alkaloids from *Pseudovibrio denitrificans* BBCC725. Tetrahedron Lett..

[44] Hodgkin D., Maslen E.N.J.B.J. (1961). The X-ray analysis of the structure of cephalosporin C. Biochem. J..

[45] Abraham E., Newton G., Hale C.J.B.j. (1954). Purification and some properties of cephalosporin N, a new penicillin. Biochem. J..

[46] Xu X., Zhang X., Nong X., Wei X., Qi S. (2015). Oxindole alkaloids from the fungus Penicillium commune DFFSCS026 isolated from deep-sea-derived sediments. Tetrahedron.

[47] Song F., He H., Ma R., Xiao X., Wei Q., Wang Q., Ji Z., Dai H., Zhang L., Capon R.J. (2016). Structure revision of the Penicillium alkaloids haenamindole and citreoindole. Tetrahedron Lett..

[48] Kim J.W., Ko S.-K., Son S., Shin K.-S., Ryoo I.-J., Hong Y.-S., Oh H., Hwang B.Y., Hirota H., Takahashi S. (2015). Haenamindole, an unusual diketopiperazine derivative from a marine-derived *Penicillium* sp. KCB12F005. Bioorg. Med. Chem. Lett..

[49] Matsunaga K., Shizuri Y., Yamamura S., Kawai K., Furukawa H. (1991). Isolation and structure of citreoindole, a new metabolite of Hybrid strain KO 0052 derived from *Penicillium citreo-viride* B. IFO 6200 and 4692. Tetrahedron Lett..

[50] Wu C.-J., Li C.-W., Gao H., Huang X.-J., Cui C.-B. (2017). Penicimutamides D–E: Two new prenylated indole alkaloids from a mutant of the marine-derived *Penicillium purpurogenum* G59. RSC Adv..

[51] Li C.-W., Wu C.-J., Cui C.-B., Xu L.-L., Cao F., Zhu H.-J. (2016). Penicimutamides A–C: Rare carbamate-containing alkaloids from a mutant of the marine-derived *Penicillium purpurogenum* G59. RSC Adv..

[52] Li H., Sun W., Deng M., Zhou Q., Wang J., Liu J., Chen C., Qi C., Luo Z., Xue Y. (2018). Asperversiamides, Linearly Fused Prenylated Indole Alkaloids from the Marine-Derived Fungus *Aspergillus versicolor*. J. Org. Chem..

[53] Hu J., Li Z., Gao J., He H., Dai H., Xia X., Liu C., Zhang L., Song F. (2019). New Diketopiperazines from a Marine-Derived Fungus Strain *Aspergillus versicolor* MF180151. Mar. Drugs.

[54] Niu S., Wang N., Xie C.-L., Fan Z., Luo Z., Chen H.-F., Yang X.-W. (2018). Roquefortine J, a novel roquefortine alkaloid, from the deep-sea-derived fungus *Penicillium granulatum* MCCC 3A00475. J. Antibiot..

[55] Luo M., Zang R., Wang X., Chen Z., Song X., Ju J., Huang H. (2019). Natural Hydroxamate-Containing Siderophore Acremonpeptides A–D and an Aluminum Complex of Acremonpeptide D from the Marine-Derived *Acremonium persicinum* SCSIO 115. J. Nat. Prod..

[56] Zhang Z., Min X., Huang J., Zhong Y., Wu Y., Li X., Deng Y., Jiang Z., Shao Z., Zhang L. (2016). Cytoglobosins H and I, New Antiproliferative Cytochalasans from Deep-Sea-Derived Fungus *Chaetomium globosum*. Mar. Drugs.

[57] Limbadri S., Luo X., Lin X., Liao S., Wang J., Zhou X., Yang B., Liu Y. (2018). Bioactive Novel Indole Alkaloids and Steroids from Deep Sea-Derived Fungus *Aspergillus fumigatus* SCSIO 41012. Molecules.

[58] Guo X.C., Xu L.L., Yang R.Y., Yang M.Y., Hu L.D., Zhu H.J., Cao F. (2019). Anti-Vibrio Indole-Diterpenoids and C-25 Epimeric Steroids From the Marine-Derived Fungus *Penicillium janthinellum*. Front. Chem..

[59] Han J., Liu M., Jenkins I.D., Liu X., Zhang L., Quinn R.J., Feng Y. (2020). Genome-Inspired Chemical Exploration of Marine Fungus *Aspergillus fumigatus* MF071. Mar. Drugs.

[60] Liu Z., Chen Y., Li S., Hu C., Liu H., Zhang W. (2021). Indole diketopiperazine alkaloids from the deep-sea-derived fungus *Aspergillus* sp. FS445. Nat. Prod. Res..

[61] Zhong W.-M., Wang J.-F., Shi X.-F., Wei X.-Y., Chen Y.-C., Zeng Q., Xiang Y., Chen X.-Y., Tian X.-P., Xiao Z.-H. (2018). Eurotiumins A–E, Five New Alkaloids from the Marine-Derived Fungus *Eurotium* sp. SCSIO F452. Mar. Drugs.

[62] Zhong W., Wang J., Wei X., Fu T., Chen Y., Zeng Q., Huang Z., Huang X., Zhang W., Zhang S. (2019). Three Pairs of New Spirocyclic Alkaloid Enantiomers From the Marine-Derived Fungus *Eurotium* sp. SCSIO F452. Front. Chem..

[63] Zhong W., Wang J., Wei X., Chen Y., Fu T., Xiang Y., Huang X., Tian X., Xiao Z., Zhang W. (2018). Variecolortins A–C, Three Pairs of Spirocyclic Diketopiperazine Enantiomers from the Marine-Derived Fungus *Eurotium* sp. SCSIO F452. Org. Lett..

[64] Fukuda T., Nagai K., Kurihara Y., Kanamoto A., Tomoda H. (2015). Graphiumins I and J, New Thiodiketopiperazines from the Marine-derived Fungus *Graphium* sp. OPMF00224. Nat. Prod. Sci..

[65] Fan Z., Sun Z.-H., Liu Z., Chen Y.-C., Liu H.-X., Li H.-H., Zhang W.-M. (2016). Dichotocejpins A–C: New Diketopiperazines from a Deep-Sea-Derived Fungus *Dichotomomyces cejpii* FS110. Mar. Drugs.

[66] Yun K., Khong T.T., Leutou A.S., Kim G.D., Hong J., Lee C.H., Son B.W. (2016). Cristazine, a New Cytotoxic Dioxopiperazine Alkaloid from the Mudflat-Sediment-Derived Fungus *Chaetomium cristatum*. Chem. Pharm. Bull..

[67] Yu G., Wang Y., Yu R., Feng Y., Wang L., Che Q., Gu Q., Li D., Li J., Zhu T. (2018). Chetracins E and F, cytotoxic epipolythiodioxopiperazines from the marine-derived fungus *Acrostalagmus luteoalbus* HDN13-530. RSC Adv..

[68] Wakefield J., Hassan H.M., Jaspars M., Ebel R., Rateb M.E. (2017). Dual Induction of New Microbial Secondary Metabolites by Fungal Bacterial Co-cultivation. Front. Microbiol..

[69] Afiyatullov S.S., Zhuravleva O.I., Antonov A.S., Berdyshev D.V., Pivkin M.V., Denisenko V.A., Popov R.S., Gerasimenko A.V., von Amsberg G., Dyshlovoy S.A. (2018). Prenylated indole alkaloids from co-culture of marine-derived fungi *Aspergillus sulphureus* and *Isaria felina*. J. Antibiot..

[70] Xu X., Zhang X., Nong X., Wang J., Qi S. (2017). Brevianamides and Mycophenolic Acid Derivatives from the Deep-Sea-Derived Fungus *Penicillium brevicompactum* DFFSCS025. Mar. Drugs.

[71] Yang J., Gong L., Guo M., Jiang Y., Ding Y., Wang Z., Xin X., An F. (2021). Bioactive Indole Diketopiperazine Alkaloids from the Marine Endophytic Fungus *Aspergillus* sp. YJ191021. Mar. Drugs.

[72] Li J., Wang Y., Hao X., Li S., Jia J., Guan Y., Peng Z., Bi H., Xiao C., Cen S. (2019). Broad-Spectrum Antiviral Natural Products from the Marine-Derived *Penicillium* sp. IMB17-046. Molecules.

[73] Yang B., Tao H., Lin X., Wang J., Liao S., Dong J., Zhou X., Liu Y. (2018). Prenylated indole alkaloids and chromone derivatives from the fungus *Penicillium* sp. SCSIO041218. Tetrahedron.

[74] Zheng Y.-Y., Shen N.-X., Liang Z.-Y., Shen L., Chen M., Wang C.-Y. (2020). Paraherquamide J, a new prenylated indole alkaloid from the marine-derived fungus *Penicillium janthinellum* HK1-6. Nat. Prod. Res..

[75] Li J., Hu Y., Hao X., Tan J., Li F., Qiao X., Chen S., Xiao C., Chen M., Peng Z. (2019). Raistrickindole A, an Anti-HCV Oxazinoindole Alkaloid from *Penicillium raistrickii* IMB17-034. J. Nat. Prod..

[76] Chen Y.X., Xu M.Y., Li H.J., Zeng K.J., Ma W.Z., Tian G.B., Xu J., Yang D.P., Lan W.J. (2017). Diverse Secondary Metabolites from the Marine-Derived Fungus *Dichotomomyces cejpii* F31-1. Mar. Drugs.

[77] Huang L.H., Xu M.Y., Li H.J., Li J.Q., Chen Y.X., Ma W.Z., Li Y.P., Xu J., Yang D.P., Lan W.J. (2017). Amino Acid-Directed Strategy for Inducing the Marine-Derived Fungus *Scedosporium apiospermum* F41-1 to Maximize Alkaloid Diversity. Org. Lett..

[78] Yuan M.X., Qiu Y., Ran Y.Q., Feng G.K., Deng R., Zhu X.F., Lan W.J., Li H.J. (2019). Exploration of Indole Alkaloids from Marine Fungus *Pseudallescheria boydii* F44-1 Using an Amino Acid-Directed Strategy. Mar. Drugs.

[79] Li C.-J., Chen P.-N., Li H.-J., Mahmud T., Wu D.-L., Xu J., Lan W.-J. (2020). Potential Antidiabetic Fumiquinazoline Alkaloids from the Marine-Derived Fungus *Scedosporium apiospermum* F41-1. J. Nat. Prod..

[80] Liu W., Li H.-J., Xu M.-Y., Ju Y.-C., Wang L.-Y., Xu J., Yang D.-P., Lan W.-J. (2015). Pseudellones A–C, Three Alkaloids from the Marine-Derived Fungus *Pseudallescheria ellipsoidea* F42-3. Org. Lett..

[81] Lan W.-J., Wang K.-T., Xu M.-Y., Zhang J.-J., Lam C.-K., Zhong G.-H., Xu J., Yang D.-P., Li H.-J., Wang L.-Y. (2016). Secondary metabolites with chemical diversity from the marine-derived fungus *Pseudallescheria boydii* F19-1 and their cytotoxic activity. RSC Adv..

[82] Wang K.T., Xu M.Y., Liu W., Li H.J., Xu J., Yang D.P., Lan W.J., Wang L.Y. (2016). Two Additional New Compounds from the Marine-Derived Fungus *Pseudallescheria ellipsoidea* F42-3. Molecules.

[83] Luo X.W., Gao C.H., Lu H.M., Wang J.M., Su Z.Q., Tao H.M., Zhou X.F., Yang B., Liu Y.H. (2020). HPLC-DAD-Guided Isolation of Diversified Chaetoglobosins from the Coral-Associated Fungus *Chaetomium globosum* C2F17. Molecules.

[84] Wei X., Feng C., Wang S.Y., Zhang D.M., Li X.H., Zhang C.X. (2020). New Indole Diketopiperazine Alkaloids from Soft Coral-Associated Epiphytic Fungus *Aspergillus* sp. EGF 15-0-3. Chem. Biodivers..

[85] Zhuravleva O.I., Antonov A.S., Trang V.T.D., Pivkin M.V., Khudyakova Y.V., Denisenko V.A., Popov R.S., Kim N.Y., Yurchenko E.A., Gerasimenko A.V. (2021). New Deoxyisoaustamide Derivatives from the Coral-Derived Fungus *Penicillium dimorphosporum* KMM 4689. Mar. Drugs.

[86] Cheng Z., Lou L., Liu D., Li X., Proksch P., Yin S., Lin W. (2016). Versiquinazolines A–K, Fumiquinazoline-Type Alkaloids from the Gorgonian-Derived Fungus *Aspergillus versicolor* LZD-14-1. J. Nat. Prod..

[87] Cheng Z., Liu D., Cheng W., Proksch P., Lin W. (2018). Versiquinazolines L–Q, new polycyclic alkaloids from the marine-derived fungus *Aspergillus versicolor*. RSC Adv..

[88] Ma X., Nong X.-H., Ren Z., Wang J., Liang X., Wang L., Qi S.-H. (2017). Antiviral peptides from marine gorgonian-derived fungus *Aspergillus* sp. SCSIO 41501. Tetrahedron Lett..

[89] Liu M., Sun W., Wang J., He Y., Zhang J., Li F., Qi C., Zhu H., Xue Y., Hu Z. (2018). Bioactive secondary metabolites from the marine-associated fungus *Aspergillus terreus*. Bioorg. Chem..

[90] Ma X., Liang X., Huang Z.-H., Qi S.-H. (2020). New alkaloids and isocoumarins from the marine gorgonian-derived fungus *Aspergillus* sp. SCSIO 41501. Nat. Prod. Res..

[91] Hou X.M., Liang T.M., Guo Z.Y., Wang C.Y., Shao C.L. (2019). Discovery, absolute assignments, and total synthesis of asperversiamides A-C and their potent activity against *Mycobacterium marinum*. Chem. Commun..

[92] Kong F.D., Fan P., Zhou L.M., Ma Q.Y., Xie Q.Y., Zheng H.Z., Zheng Z.H., Zhang R.S., Yuan J.Z., Dai H.F. (2019). Penerpenes A-D, Four Indole Terpenoids with Potent Protein Tyrosine Phosphatase Inhibitory Activity from the Marine-Derived Fungus *Penicillium* sp. KFD28. Org. Lett..

[93] Zhou L.M., Kong F.D., Fan P., Ma Q.Y., Xie Q.Y., Li J.H., Zheng H.Z., Zheng Z.H., Yuan J.Z., Dai H.F. (2019). Indole-Diterpenoids with Protein Tyrosine Phosphatase Inhibitory Activities from the Marine-Derived Fungus *Penicillium* sp. KFD28. J. Nat. Prod..

[94] Chen M.Y., Xie Q.Y., Kong F.D., Ma Q.Y., Zhou L.M., Yuan J.Z., Dai H.F., Wu Y.G., Zhao Y.X. (2020). Two new indole-diterpenoids from the marine-derived fungus *Penicillium* sp. KFD28. J. Asian Nat. Prod. Res..

[95] Kong F.D., Zhang S.L., Zhou S.Q., Ma Q.Y., Xie Q.Y., Chen J.P., Li J.H., Zhou L.M., Yuan J.Z., Hu Z. (2019). Quinazoline-Containing Indole Alkaloids from the Marine-Derived Fungus *Aspergillus* sp. HNMF114. J. Nat. Prod..

[96] Liu S.S., Yang L., Kong F.D., Zhao J.H., Yao L., Yuchi Z.G., Ma Q.Y., Xie Q.Y., Zhou L.M., Guo M.F. (2021). Three New Quinazoline-Containing Indole Alkaloids From the Marine-Derived Fungus *Aspergillus* sp. HNMF114. Front. Microbiol..

[97] Zhang P., Li X.-M., Liu H., Li X., Wang B.-G. (2015). Two new alkaloids from *Penicillium oxalicum* EN-201, an endophytic fungus derived from the marine mangrove plant *Rhizophora stylosa*. Phytochem. Lett..

[98] Meng L.H., Wang C.Y., Mándi A., Li X.M., Hu X.Y., Kassack M.U., Kurtán T., Wang B.G. (2016). Three Diketopiperazine Alkaloids with Spirocyclic Skeletons and One Bisthiodiketopiperazine Derivative from the Mangrove-Derived Endophytic Fungus *Penicillium brocae* MA-231. Org. Lett..

[99] Gao S.S., Li X.M., Williams K., Proksch P., Ji N.Y., Wang B.G. (2016). Rhizovarins A-F, Indole-Diterpenes from the Mangrove-Derived Endophytic Fungus *Mucor irregularis* QEN-189. J. Nat. Prod..

[100] Huang S., Chen H., Li W., Zhu X., Ding W., Li C. (2016). Bioactive Chaetoglobosins from the Mangrove Endophytic Fungus *Penicillium chrysogenum*. Mar. Drugs.

[101] Zhu X., Zhou D., Liang F., Wu Z., She Z., Li C. (2017). Penochalasin K, a new unusual chaetoglobosin from the mangrove endophytic fungus *Penicillium chrysogenum* V11 and its effective semi-synthesis. Fitoterapia.

[102] Yu G., Zhou G., Zhu M., Wang W., Zhu T., Gu Q., Li D. (2016). Neosartoryadins A and B, Fumiquinazoline Alkaloids from a Mangrove-Derived Fungus *Neosartorya udagawae* HDN13-313. Org. Lett..

[103] Zheng C.J., Bai M., Zhou X.M., Huang G.L., Shao T.M., Luo Y.P., Niu Z.G., Niu Y.Y., Chen G.Y., Han C.R. (2018). Penicilindoles A-C, Cytotoxic Indole Diterpenes from the Mangrove-Derived Fungus *Eupenicillium* sp. HJ002. J. Nat. Prod..

[104] May Zin W.W., Buttachon S., Dethoup T., Pereira J.A., Gales L., Inácio Â., Costa P.M., Lee M., Sekeroglu N., Silva A.M.S. (2017). Antibacterial and antibiofilm activities of the metabolites isolated from the culture of the mangrove-derived endophytic fungus *Eurotium chevalieri* KUFA 0006. Phytochemistry.

[105] Du F.-Y., Li X., Li X.-M., Zhu L.-W., Wang B.-G. (2017). Indolediketopiperazine Alkaloids from Eurotium cristatum EN-220, an Endophytic Fungus Isolated from the Marine Alga *Sargassum thunbergii*. Mar. Drugs.

[106] Zhang P., Li X.M., Mao X.X., Mándi A., Kurtán T., Wang B.G. (2016). Varioloid A, a new indolyl-6,10b-dihydro-5aH-[1]benzofuro[2,3-b]indole derivative from the marine alga-derived endophytic fungus *Paecilomyces variotii* EN-291. Beilstein J. Org. Chem..

[107] Cao J., Li X.M., Meng L.H., Konuklugil B., Li X., Li H.L., Wang B.G. (2019). Isolation and characterization of three pairs of indolediketopiperazine enantiomers containing infrequent N-methoxy substitution from the marine algal-derived endophytic fungus *Acrostalagmus luteoalbus* TK-43. Bioorg. Chem..

[108] Liu W., Wang L., Wang B., Xu Y., Zhu G., Lan M., Zhu W., Sun K. (2019). Diketopiperazine and Diphenylether Derivatives from Marine Algae-Derived *Aspergillus versicolor* OUCMDZ-2738 by Epigenetic Activation. Mar. Drugs.

[109] Yang S.-Q., Li X.-M., Li X., Chi L.-P., Wang B.-G. (2018). Two New Diketomorpholine Derivatives and a New Highly Conjugated Ergostane-Type Steroid from the Marine Algal-Derived Endophytic Fungus *Aspergillus alabamensis* EN-547. Mar. Drugs.

[110] Yurchenko E.A., Menchinskaya E.S., Pislyagin E.A., Trinh P.T., Ivanets E.V., Smetanina O.F., Yurchenko A.N. (2018). Neuroprotective Activity of Some Marine Fungal Metabolites in the 6-Hydroxydopamin- and Paraquat-Induced Parkinson’s Disease Models. Mar. Drugs.

[111] Ma X., Peng J., Wu G., Zhu T., Li G., Gu Q., Li D. (2015). Speradines B-D, oxygenated cyclopiazonic acid alkaloids from the sponge-derived fungus *Aspergillus flavus* MXH-X104. Tetrahedron.

[112] Harms H., Orlikova B., Ji S., Nesaei-Mosaferan D., König G.M., Diederich M. (2015). Epipolythiodiketopiperazines from the Marine Derived Fungus *Dichotomomyces cejpii* with NF-κB Inhibitory Potential. Mar. Drugs.

[113] May Zin W.W., Buttachon S., Dethoup T., Fernandes C., Cravo S., Pinto M.M., Gales L., Pereira J.A., Silva A.M., Sekeroglu N. (2016). New Cyclotetrapeptides and a New Diketopiperzine Derivative from the Marine Sponge-Associated Fungus *Neosartorya glabra* KUFA 0702. Mar. Drugs.

[114] Cho K.H., Sohn J.H., Oh H. (2018). Isolation and structure determination of a new diketopiperazine dimer from marine-derived fungus *Aspergillus* sp. SF-5280. Nat. Prod. Res..

[115] Özkaya F.C., Ebrahim W., El-Neketi M., Tansel Tanrıkul T., Kalscheuer R., Müller W.E.G., Guo Z., Zou K., Liu Z., Proksch P. (2018). Induction of new metabolites from sponge-associated fungus *Aspergillus carneus* by OSMAC approach. Fitoterapia.

[116] Buttachon S., Ramos A.A., Inácio Â., Dethoup T., Gales L., Lee M., Costa P.M., Silva A.M.S., Sekeroglu N., Rocha E. (2018). Bis-Indolyl Benzenoids, Hydroxypyrrolidine Derivatives and Other Constituents from Cultures of the Marine Sponge-Associated Fungus *Aspergillus candidus* KUFA0062. Mar. Drugs.

[117] Liu J., Gu B., Yang L., Yang F., Lin H. (2018). New Anti-inflammatory Cyclopeptides From a Sponge-Derived Fungus *Aspergillus violaceofuscus*. Front. Chem..

[118] Cao T., Ling J., Liu Y., Chen X., Tian X., Meng D., Pan H., Hu J., Wang N. (2018). Characterization and abolishment of the cyclopiazonic acids produced by *Aspergillus oryzae* HMP-F28. Biosci. Biotechnol. Biochem..

[119] Luo X.-W., Lin Y., Lu Y.-J., Zhou X.-F., Liu Y.-H. (2019). Peptides and polyketides isolated from the marine sponge-derived fungus *Aspergillus terreus* SCSIO 41008. Chin. J. Nat. Med..

[120] Guo C., Wang P., Lin X., Salendra L., Kong F., Liao S., Yang B., Zhou X., Wang J., Liu Y. (2019). Phloroglucinol heterodimers and bis-indolyl alkaloids from the sponge-derived fungus *Aspergillus* sp. SCSIO 41018. Org. Chem. Front..

[121] Elsebai M.F., Schoeder C.T., Muller C.E. (2021). Fintiamin: A diketopiperazine from the marine sponge-derived fungus *Eurotium* sp.. Arch. Der Pharm..

[122] Hu X.Y., Meng L.H., Li X., Yang S.Q., Li X.M., Wang B.G. (2017). Three New Indole Diterpenoids from the Sea-Anemone-Derived Fungus *Penicillium* sp. AS-79. Mar. Drugs.

[123] Ivanets E.V., Yurchenko A.N., Smetanina O.F., Rasin A.B., Zhuravleva O.I., Pivkin M.V., Popov R.S., von Amsberg G., Afiyatullov S.S., Dyshlovoy S.A. (2018). Asperindoles A–D and a p-Terphenyl Derivative from the Ascidian-Derived Fungus *Aspergillus* sp. KMM 4676. Mar. Drugs.

[124] Liu L., Xu W., Li S., Chen M., Cheng Y., Yuan W., Cheng Z., Li Q. (2019). Penicindopene A, a new indole diterpene from the deep-sea fungus *Penicillium* sp. YPCMAC1. Nat. Prod. Res..

[125] Zhou R., Liao X., Li H., Li J., Feng P., Zhao B., Xu S. (2018). Isolation and Synthesis of Misszrtine A: A Novel Indole Alkaloid From Marine Sponge-Associated *Aspergillus* sp. SCSIO XWS03F03. Front. Chem..

[126] Yan W., Zhao S.S., Ye Y.H., Zhang Y.Y., Zhang Y., Xu J.Y., Yin S.M., Tan R.X. (2019). Generation of Indoles with Agrochemical Significance through Biotransformation by *Chaetomium globosum*. J. Nat. Prod..

[127] Zhang Y.H., Geng C., Zhang X.W., Zhu H.J., Shao C.L., Cao F., Wang C.Y. (2019). Discovery of Bioactive Indole-Diketopiperazines from the Marine-Derived Fungus *Penicillium brasilianum* Aided by Genomic Information. Mar. Drugs.

[128] Li P., Zhang M., Li H., Wang R., Hou H., Li X., Liu K., Chen H. (2021). New Prenylated Indole Homodimeric and Pteridine Alkaloids from the Marine-Derived Fungus *Aspergillus austroafricanus* Y32-2. Mar. Drugs.

[129] Wang P., Zhao S., Ding W., Qiu F., Xu J. (2015). Asperginine, an Unprecedented Alkaloid from the Marine-derived Fungus *Aspergillus* sp.. Nat. Prod. Commun..

[130] Xu J., Hu Q., Ding W., Wang P., Di Y. (2018). New asymmetrical bispyrrolidinoindoline diketopiperazines from the marine fungus *Aspergillus* sp. DX4H. Nat. Prod. Res..

[131] Kwon J., Lee H., Ko W., Kim D.-C., Kim K.-W., Kwon H.C., Guo Y., Sohn J.H., Yim J.H., Kim Y.-C. (2017). Chemical constituents isolated from Antarctic marine-derived *Aspergillus* sp. SF-5976 and their anti-inflammatory effects in LPS-stimulated RAW 264.7 and BV2 cells. Tetrahedron.

[132] Takahashi K., Sakai K., Fukasawa W., Nagano Y., Sakaguchi S.O., Lima A.O., Pellizari V.H., Iwatsuki M., Takishita K., Yoshida T. (2018). Quellenin, a new anti-Saprolegnia compound isolated from the deep-sea fungus, *Aspergillus* sp. YK-76. J. Antibiot..

[133] Kubota T., Nakamura K., Sakai K., Fromont J., Gonoi T., Kobayashi J.i. (2016). Hyrtinadines C and D, New Azepinoindole-Type Alkaloids from a Marine Sponge *Hyrtios* sp.. Chem. Pharm. Bull..

[134] Takahashi H., Kurimoto S.-i., Kobayashi J.i., Kubota T. (2018). Ishigadine A, a new canthin-6-one alkaloid from an Okinawan marine sponge *Hyrtios* sp.. Tetrahedron Lett..

[135] Shady N.H., Abdelmohsen U.R., Ahmed S., Fouad M., Kamel M.S.J.J.P.P. (2017). Phytochemical and biological investigation of the red sea marine sponge *Hyrtios* sp.. J. Pharmacogn. Phytochem..

[136] Shady N.H., Fouad M.A., Ahmed S., Pimentel-Elardo S.M., Nodwell J.R., Kamel M.S., Abdelmohsen U.R. (2018). A new antitrypanosomal alkaloid from the Red Sea marine sponge *Hyrtios* sp.. J. Antibiot..

[137] Park S.-I., Lee Y.-J., Won H., Oh K.-B., Lee H.-S. (2018). Indole Alkaloids from Tropical Sponge *Hyrtios* sp. as Isocitrate Lyase Inhibitors. Nat. Prod. Commun..

[138] Bagalagel A.A., Bogari H.A., Ahmed S.A., Diri R.M., Elhady S.S.J.H. (2018). New Bromoindole Alkaloid Isolated from the Marine Sponge *Hyrtios erectus*. Heterocycles.

[139] Wang Q., Tang X.L., Luo X.C., de Voog N.J., Li P.L., Li G.Q. (2019). Aplysinopsin-type and Bromotyrosine-derived Alkaloids from the South China Sea Sponge *Fascaplysinopsis reticulata*. Sci. Rep..

[140] Campos P.E., Pichon E., Moriou C., Clerc P., Trépos R., Frederich M., De Voogd N., Hellio C., Gauvin-Bialecki A., Al-Mourabit A. (2019). New Antimalarial and Antimicrobial Tryptamine Derivatives from the Marine Sponge *Fascaplysinopsis reticulata*. Mar. Drugs.

[141] Ibrahim S.R.M., Mohamed G.A. (2016). Ingenines C and D, new cytotoxic pyrimidine-β-carboline alkaloids from the Indonesian sponge *Acanthostrongylophora ingens*. Phytochem. Lett..

[142] Ibrahim S.R.M., Mohamed G.A. (2017). Ingenine E, a new cytotoxic β-carboline alkaloid from the Indonesian sponge *Acanthostrongylophora ingens*. J. Asian Nat. Prod. Res..

[143] Ibrahim S., Mohamed G., Al Haidari R., El-Kholy A., Zayed M. (2018). Ingenine F: A New Cytotoxic Tetrahydro Carboline Alkaloid from the Indonesian Marine Sponge *Acanthostrongylophora ingens*. Pharmacogn. Mag..

[144] Kim C.K., Riswanto R., Won T.H., Kim H., Elya B., Sim C.J., Oh D.C., Oh K.B., Shin J. (2017). Manzamine Alkaloids from an *Acanthostrongylophora* sp. Sponge. J. Nat. Prod..

[145] Okada M., Sugita T., Wong C.P., Wakimoto T., Abe I. (2017). Identification of Pyridinium with Three Indole Moieties as an Antimicrobial Agent. J. Nat. Prod..

[146] Liu F.-L., Yang X.-L. (2017). Indole Derivatives Produced by the Metagenome Genes of the *Escherichia coli*-Harboring Marine Sponge *Discodermia calyx*. Molecules.

[147] Yang I., Choi H., Nam S.-J., Kang H. (2015). Two Indole-Alkaloids from a Korean Marine Sponge *Spongia* sp.. Bull. Korean Chem. Soc..

[148] Al-Massarani S.M., El-Gamal A.A., Al-Said M.S., Abdel-Kader M.S., Ashour A.E., Kumar A., Abdel-Mageed W.M., Al-Rehaily A.J., Ghabbour H.A., Fun H.K. (2016). Studies on the Red Sea Sponge *Haliclona* sp. for its Chemical and Cytotoxic Properties. Pharmacogn. Mag..

[149] Hitora Y., Takada K., Ise Y., Okada S., Matsunaga S. (2016). Dragmacidins G and H, Bisindole Alkaloids Tethered by a Guanidino Ethylthiopyrazine Moiety, from a *Lipastrotethya* sp. Marine Sponge. J. Nat. Prod..

[150] Wang D., Feng Y., Murtaza M., Wood S., Mellick G., Hooper J.N., Quinn R.J. (2016). A Grand Challenge: Unbiased Phenotypic Function of Metabolites from *Jaspis splendens* against Parkinson’s Disease. J. Nat. Prod..

[151] Ebada S.S., Müller W.E.G., Lin W., Proksch P. (2019). New Acyclic Cytotoxic Jasplakinolide Derivative from the Marine Sponge *Jaspis splendens*. Mar. Drugs.

[152] Jamison M.T., Molinski T.F. (2016). Jamaicensamide A, a Peptide Containing β-Amino-α-keto and Thiazole-Homologated η-Amino Acid Residues from the Sponge *Plakina jamaicensis*. J. Nat. Prod..

[153] Olsen E.K., Hansen E., Moodie L.M.K., Isaksson J., Sepčić K., Cergolj M., Svenson J., Andersen J.H. (2016). Marine AchE inhibitors isolated from *Geodia barretti*: Natural compounds and their synthetic analogs. Org. Biomol. Chem..

[154] Di X., Rouger C., Hardardottir I., Freysdottir J., Molinski T.F., Tasdemir D., Omarsdottir S. (2018). 6-Bromoindole Derivatives from the Icelandic Marine Sponge *Geodia barretti*: Isolation and Anti-Inflammatory Activity. Mar. Drugs.

[155] Liu H.B., Lauro G., O‘Connor R.D., Lohith K., Kelly M., Colin P., Bifulco G., Bewley C.A. (2017). Tulongicin, an Antibacterial Tri-Indole Alkaloid from a Deep-Water *Topsentia* sp. Sponge. J. Nat. Prod..

[156] Lorig-Roach N., Hamkins-Indik F., Johnson T.A., Tenney K., Valeriote F.A., Crews P. (2018). The potential of achiral sponge-derived and synthetic bromoindoles as selective cytotoxins against PANC-1 tumor cells. Tetrahedron.

[157] Cruz P.G., Martínez Leal J.F., Daranas A.H., Pérez M., Cuevas C. (2018). On the Mechanism of Action of Dragmacidins I and J, Two New Representatives of a New Class of Protein Phosphatase 1 and 2A Inhibitors. ACS Omega.

[158] Salib M.N., Molinski T.F. (2018). Six Trikentrin-like Cyclopentanoindoles from *Trikentrion flabelliforme*. Absolute Structural Assignment by NMR and ECD. J. Org. Chem..

[159] Jennings L.K., Khan N.M.D., Kaur N., Rodrigues D., Morrow C., Boyd A., Thomas O.P. (2019). Brominated Bisindole Alkaloids from the Celtic Sea Sponge *Spongosorites calcicola*. Molecules.

[160] Ragini K., Piggott A.M., Karuso P. (2019). Bisindole Alkaloids from a New Zealand Deep-Sea Marine Sponge *Lamellomorpha strongylata*. Mar. Drugs.

[161] Guzii A.G., Makarieva T.N., Denisenko V.A., Gerasimenko A.V., Udovenko A.A., Popov R.S., Dmitrenok P.S., Golotin V.A., Fedorov S.N., Grebnev B.B. (2019). Guitarrins A-E and Aluminumguitarrin A: 5-Azaindoles from the Northwestern Pacific Marine Sponge *Guitarra fimbriata*. J. Nat. Prod..

[162] El-Hawary S.S., Sayed A.M., Mohammed R., Hassan H.M., Rateb M.E., Amin E., Mohammed T.A., El-Mesery M., Bin Muhsinah A., Alsayari A. (2019). Bioactive Brominated Oxindole Alkaloids from the Red Sea Sponge *Callyspongia siphonella*. Mar. Drugs.

[163] Ki D.W., Kodama T., El-Desoky A.H., Wong C.P., Nguyen H.M., Do K.M., Thai Q.M., Ton Nu L.H., Morita H. (2020). Chemical Constituents of the Vietnamese Marine Sponge *Gelliodes* sp. and Their Cytotoxic Activities. Chem. Biodivers..

[164] Moosmann P., Taniguchi T., Furihata K., Utsumi H., Ise Y., Morii Y., Yamawaki N., Takatani T., Arakawa O., Okada S. (2021). Myrindole A, an Antimicrobial Bis-indole from a Marine Sponge *Myrmekioderma* sp.. Org. Lett..

[165] Ovenden S.P., Capon R.J. (1999). Echinosulfonic acids A-C and echinosulfone A: Novel bromoindole sulfonic acids and a sulfone from a southern australian marine sponge, *Echinodictyum*. J. Nat. Prod..

[166] Rubnov S., Chevallier C., Thoison O., Debitus C., Laprevote O., Guénard D., Sévenet T. (2005). Echinosulfonic acid D: An ESI MSn evaluation of a new cytotoxic alkaloid from the New-Caledonian sponge *Psammoclemma* sp.. Nat. Prod. Res..

[167] Holland D.C., Kiefel M.J., Carroll A.R. (2020). Structure Revisions of the Sponge-Derived Dibrominated Bis-indole Alkaloids, Echinosulfone A and the Echinosulfonic Acids A to D. J. Org. Chem..

[168] Neupane P., Salim A.A., Capon R.J. (2020). Structure revision of the rare sponge metabolite echinosulfone A, and biosynthetically related echinosulfonic acids A–D. Tetrahedron Lett..

[169] Sala S., Nealon G.L., Sobolev A.N., Fromont J., Gomez O., Flematti G.R. (2020). Structure Reassignment of Echinosulfone A and the Echinosulfonic Acids A–D Supported by Single-Crystal X-ray Diffraction and Density Functional Theory Analysis. J. Nat. Prod..

[170] Hanif N., Yamada K., Kitamura M., Kawazoe Y., de Voogd N.J., Uemura D. (2015). New Indole Alkaloids from the Sponge *Plakortis* sp.. Chem. Nat. Compd..

[171] Kubota T., Nakamura K., Kurimoto S.I., Sakai K., Fromont J., Gonoi T., Kobayashi J. (2017). Zamamidine D, a Manzamine Alkaloid from an Okinawan *Amphimedon* sp. Marine Sponge. J. Nat. Prod..

[172] Tran T.D., Cartner L.K., Bokesch H.R., Henrich C.J., Wang X.W., Mahidol C., Ruchirawat S., Kittakoop P., O’Keefe B.R., Gustafson K.R. (2021). NMR characterization of rearranged staurosporine aglycone analogues from the marine sponge *Damiria* sp.. Magn. Reson. Chem..

[173] Taufa T., Gordon R.M.A., Hashmi M.A., Hira K., Miller J.H., Lein M., Fromont J., Northcote P.T., Keyzers R.A. (2019). Pyrroloquinoline derivatives from a Tongan specimen of the marine sponge *Strongylodesma tongaensis*. Tetrahedron Lett..

[174] Tabudravu J.N., Pellissier L., Smith A.J., Subko K., Autréau C., Feussner K., Hardy D., Butler D., Kidd R., Milton E.J. (2019). LC-HRMS-Database Screening Metrics for Rapid Prioritization of Samples to Accelerate the Discovery of Structurally New Natural Products. J. Nat. Prod..

[175] Miguel-Gordo M., Gegunde S., Calabro K., Jennings L.K., Alfonso A., Genta-Jouve G., Vacelet J., Botana L.M., Thomas O.P. (2019). Bromotryptamine and Bromotyramine Derivatives from the Tropical Southwestern Pacific Sponge *Narrabeena nigra*. Mar. Drugs.

[176] Khushi S., Nahar L., Salim A.A., Capon R.J. (2020). Trachycladindoles H-M: Molecular Networking Guided Exploration of a Library of Southern Australian Marine Sponges. Aust. J. Chem..

[177] Knestrick M.A., Wilson N.G., Roth A., Adams J.H., Baker B.J. (2019). Friomaramide, a Highly Modified Linear Hexapeptide from an Antarctic Sponge, Inhibits *Plasmodium falciparum* Liver-Stage Development. J. Nat. Prod..

[178] Hahn D., Kim H., Yang I., Chin J., Hwang H., Won D.H., Lee B., Nam S.J., Ekins M., Choi H. (2016). The Halicylindramides, Farnesoid X Receptor Antagonizing Depsipeptides from a *Petrosia* sp. Marine Sponge Collected in Korea. J. Nat. Prod..

[179] Bewley C.A., Debitus C., Faulkner D.J. (1994). Microsclerodermins A and B. Antifungal Cyclic Peptides from the Lithistid Sponge *Microscleroderma* sp.. J. Am. Chem. Soc..

[180] Zhang X., Jacob M.R., Rao R.R., Wang Y.-H., Agarwal A.K., Newman D.J., Khan I.A., Clark A.M., Li X.-C. (2012). Antifungal cyclic peptides from the marine sponge *Microscleroderma herdmani*. Res. Rep. Med. Chem..

[181] Melikhova E.Y., Pullin R.D., Winter C., Donohoe T.J. (2016). Dehydromicrosclerodermin B and Microsclerodermin J: Total Synthesis and Structural Revision. Angew. Chem..

[182] Golantsov N.E., Festa A.A., Varlamov A.V., Voskressensky L.G. (2017). Revision of the Structure and Total Synthesis of Topsentin C. Synthesis.

[183] Shaker K.H., Göhl M., Müller T., Seifert K. (2015). Indole Alkaloids from the Sea Anemone Heteractis aurora and Homarine from *Octopus cyanea*. Chem. Biodivers..

[184] Guzii A.G., Makarieva T.N., Fedorov S.N., Denisenko V.A., Dmitrenok P.S., Kuzmich A.S., Krasokhin V.B., Lee H.S., Lee Y.J., Stonik V.A. (2016). Gramine-derived Bromo-alkaloids Activating NF—kB-dependent Transcription from the Marine Hydroid *Abietinaria abietina*. Nat. Prod. Commun..

[185] Hansen K., Andersen J.H., Bayer A., Pandey S.K., Lorentzen M., Jørgensen K.B., Sydnes M.O., Guttormsen Y., Baumann M., Koch U. (2019). Kinase Chemodiversity from the Arctic: The Breitfussins. J. Med. Chem..

[186] Hansen K., Isaksson J., Bayer A., Johansen J.A., Andersen J.H., Hansen E. (2017). Securamine Derivatives from the Arctic Bryozoan *Securiflustra securifrons*. J. Nat. Prod..

[187] Dos Santos L.A., Clavico E.E., Parra L.L., Berlinck R.G., Ferreira A.G., Paul V.J., Pereira R.C. (2017). Evaluation of chemical defense and chemical diversity in the exotic bryozoan *Amathia verticillata*. J. Braz. Chem. Soc..

[188] Kleks G., Holland D.C., Kennedy E.K., Avery V.M., Carroll A.R. (2020). Antiplasmodial Alkaloids from the Australian Bryozoan *Amathia lamourouxi*. J. Nat. Prod..

[189] Maltseva A.L., Kotenko O.N., Kutyumov V.A., Matvienko D.A., Shavarda A.L., Winson M.K., Ostrovsky A.N. (2017). Novel brominated metabolites from Bryozoa: A functional analysis. Nat. Prod. Res..

[190] Hahn D., Kim G.J., Choi H., Kang H. (2015). A Novel Bromoindole Alkaloid from a Korean Colonial Tunicate *Didemnum* sp.. Nat. Prod. Sci..

[191] Tran T.D., Pham N.B., Ekins M., Hooper J.N.A., Quinn R.J. (2015). Isolation and Total Synthesis of Stolonines A–C, Unique Taurine Amides from the Australian Marine Tunicate *Cnemidocarpa stolonifera*. Mar. Drugs.

[192] Goudou F., Petit P., Moriou C., Gros O., Al-Mourabit A. (2017). Orbicularisine: A Spiro-Indolothiazine Isolated from Gills of the Tropical Bivalve *Codakia orbicularis*. J. Nat. Prod..

[193] Wang S.-S., Cheng Y.-B., Lin Y.-C., Liaw C.-C., Chang J.-Y., Kuo Y.-H., Shen Y.-C. (2015). Nitrogen-Containing Diterpenoids, Sesquiterpenoids, and Nor-Diterpenoids from *Cespitularia taeniata*. Mar. Drugs.

[194] Iwasaki K., Iwasaki A., Sumimoto S., Sano T., Hitomi Y., Ohno O., Suenaga K. (2018). Croissamide, a proline-rich cyclic peptide with an N-prenylated tryptophan from a marine cyanobacterium *Symploca* sp.. Tetrahedron Lett..

[195] Phyo M.Y., Ding C.Y.G., Goh H.C., Goh J.X., Ong J.F.M., Chan S.H., Yung P.Y.M., Candra H., Tan L.T. (2019). Trikoramide A, a Prenylated Cyanobactin from the Marine Cyanobacterium *Symploca hydnoides*. J. Nat. Prod..

[196] Woolner V.H., Jones C.M., Field J.J., Fadzilah N.H., Munkacsi A.B., Miller J.H., Keyzers R.A., Northcote P.T. (2016). Polyhalogenated Indoles from the Red Alga *Rhodophyllis membranacea*: The First Isolation of Bromo-Chloro-Iodo Secondary Metabolites. J. Nat. Prod..

[197] Li M.C., Sun W.S., Cheng W., Liu D., Liang H., Zhang Q.Y., Lin W.H. (2016). Four new minor brominated indole related alkaloids with antibacterial activities from *Laurencia similis*. Bioorg. Med. Chem. Lett..

[198] Cai Y.S., Sun J.Z., Tang Q.Q., Fan F., Guo Y.W. (2018). Acanthiline A, a pyrido[1,2-a]indole alkaloid from Chinese mangrove *Acanthus ilicifolius*. J. Asian Nat. Prod. Res..

[199] Liu T., Li Z.L., Wang Y., Tian L., Pei Y.H., Hua H.M. (2011). A new alkaloid from the marine-derived fungus *Hypocrea virens*. Nat. Prod. Res..

[200] Green M.T., Peczkowski G.R., Al-Ani A.J., Benjamin S.L., Simpkins N.S., Jones A.M. (2017). Total synthesis and structural revision of a mangrove alkaloid. RSC Adv..

