# Supplementary material for: Marine Indole Alkaloids—Isolation, Structure and Bioactivities
Source: Mar Drugs. 2021 Nov 24;19(12):658. doi: 10.3390/md19120658 (PMC8708922; doi:10.3390/md19120658)
Supplement: Supplementary file 1 [file marinedrugs-19-00658-s001.zip › marinedrugs-1464094-supplementary.pdf]

Supporting Information

Marine Indole Alkaloids - Isolation, Structure and Bioactivities

Yong Hu†, Siling Chen†, Fang Yang and Shuai Dong *

[1] Key Laboratory of Tropical Biological Resources of Ministry of Education, School of Pharmaceutical Sciences, Hainan University, Haikou 570228, China; hy15260001800@163.com (Y.H.); siling0420@163.com (S.C.); yf200110237@163.com (F.Y.)
* Correspondence: dongshuai_1024@163.com (S.D.)
† Both contributed equally to this work.

Table S1. General information of the cell lines

Abbreviation	Organism	Tissue	Disease
3T3-L1	Mouse	Embryo	Fibroblast normal cell line
5637	Human	Bladder	Grade II Carcinoma
22RV1	Human	Prostate	Carcinoma
786O	Human	Kidney	Adenocarcinoma; Renal Cell
A204	Human	Muscle	Rhabdomyosarcoma
A2058	Human	Skin	Melanoma
A2780	Human	Ovary	Ovarian endometrioid adenocarcinoma
A2780-CisR	Human	Ovary	Ovarian endometrioid adenocarcinoma
A2780T	Human	Ovary	Ovarian endometrioid adenocarcinoma
A375	Human	Skin	Malignant Melanoma
A431	Human	Skin	Epidermoid Carcinoma
A498	Human	Kidney	Carcinoma
A549	Human	Lung	Carcinoma
A673	Human	Muscle	Ewings Sarcoma
ACHN	Human	Kidney	Adenocarcinoma; Renal Cell
AML-2	Human	Hematological	Acute myeloid leukemia
ASPC1	Human	Pancreas	Adenocarcinoma
B16	Mouse	Skin	Melanoma
B16-F10	Mouse	Skin	Melanoma
BEL-7042			Inexistence
BEL-7402	Human	Liver	Hepatocellular
BGC-823	Human	Stomach	Gastric adenocarcinoma
BSC-1	Cercopithecus aethiops	Kidney	Normal cell line
BT20	Human	Breast	Carcinoma
BT-549	Human	Breast; Mammary gland	Carcinoma; Ductal
BV-2	Mouse	Brain; microglia	Normal cell line
BXPC3	Human	Pancreas	Adenocarcinoma
C33A	Human	Cervix	Carcinoma
CACO2	Human	Colon	Colorectal Adenocarcinoma

CAKI-1	Human	Kidney	Clear cell Carcinoma
Calu3	Human	Lung	Lung adenocarcinoma
CALU6	Human	Lung	Carcinoma; Anaplastic
CASKI	Human	Cervix	Epidermoid Carcinoma
CCRF-CEM	Human	Peripheral blood	Acute lymphoblastic leukemia ALL
CLS-439	Human	Bladder	Bladder carcinoma
CNE-1	Human	Nasopharynx	Nasopharyngeal carcinoma
CNE-2	Human	Nasopharynx	Nasopharyngeal carcinoma
COLO 205	Human	Large intestine; Colon	Adenocarcinoma; Colorectal; Dukes' type D
COLO 678	Human	Colon	Colon carcinoma;
CV-1	Cercopithecus aethiops	Kidney	Normal cell line
Daoy	Human	Cerebellum	Medulloblastoma
DLD1	Human	Colon	Adenocarcinoma; Colorectal; Dukes' type C
DU-145	Human	Prostate	Carcinoma
EFO-21	Human	Ovary	Ovarian serous cystadenocarcinoma
EJ-28	Human	Bladder	Bladder carcinoma
EKVX	Human	Lung	Lung adenocarcinoma
GLC82	Human	Uterus; Cervix	HPV-related endocervical adenocarcinoma
GRANTA-519	Human	Hematological	Mantle cell lymphoma
GXF 251	Human	Stomach	Gastric carcinoma
H1650	Human	Lung	Minimally invasive lung adenocarcinoma
HCC-2998	Human	Colon	Colon adenocarcinoma
HCT116	Human	Colon	Carcinoma; Colorectal
HCT15	Human	Colon	Adenocarcinoma; Colorectal; Dukes' type C
HEK293	Human	Kidney	Normal cell line
HELA	Human	Uterus; Cervix	Adenocarcinoma
HEPG2	Human	Liver	Carcinoma; Hepatocellular
HL-60	Human	Peripheral blood	Acute promyelocytic leukemia
HONE1	Human	Nasopharynx	Nasopharyngeal carcinoma
HOP-62	Human	Lung	Lung adenocarcinoma
HOP-92	Human	Lung	Lung non-small cell carcinoma
HS-578T	Human	Breast; Mammary gland	Invasive breast carcinoma
HS-729	Human	Muscle	Rhabdomyosarcoma
HT-1080	Human	Connective tissue	Fibrosarcoma
HT-29	Human	Colon	Adenocarcinoma; Colorectal
IGROV-1	Human	Ovary	Ovarian endometrioid adenocarcinoma
IMR90	Human	Lung	Normal cell line
J82	Human	Bladder	Transitional Cell Carcinoma
JAR	Human	Placenta	Choriocarcinoma
JEG3	Human	Placenta	Choriocarcinoma
JIMT-1	Human	Breast	Breast ductal carcinoma
Jurkat	Human	Hematological	Childhood T acute lymphoblastic leukemia
K562	Human	Bone; marrow	Chronic myeloid leukemia at blast crisis
KASUMI-1	Human	Hematological	Acute myeloblastic leukemia

KB	Human	Mouth	Carcinoma
KM12	Human	Colon	Colon carcinoma
L1210	Mouse	Skin	Lymphocytic leukemia
L-363	Human	Hematological	Plasma cell myeloma; Multiple myeloma
L-5178-Y	Mouse	Hematological	Mouse leukemia
L929	Mouse	Lung	Normal cell line
LNCaP	Human	Prostate	Carcinoma
LOVO	Human	Colon	Adenocarcinoma; Dukes' type C, grade IV
LOX-IMVI	Human	Axillary lymph node	Amelanotic melanoma
LU-1	Human	Lung	Adenocarcinoma
LXFA-629	Human	Lung	Lung adenocarcinoma
LXFL-529	Human	Lung	Lung non-small cell carcinoma
M14	Human	Subcutaneous; right buttock	Amelanotic melanoma
MALME-3M	Human	Skin	Malignant Melanoma
MAXF-401	Human	Breast	Breast carcinoma
MCF10A	Human	Breast	Normal cell line
MCF7	Human	Breast	Adenocarcinoma
MDA-MB-231	Human	Breast	Adenocarcinoma
MDA-MB-435	Human	Breast	Melanoma
MDA-MB-436	Human	Breast	Adenocarcinoma
MDA-MB-468	Human	Breast	Adenocarcinoma
MDA-N	Human	Subcutaneous; right buttock	Amelanotic melanoma
MEXF-462	Human		Melanoma
MG63	Human	Bone	Osteosarcoma
MHH-ES-1	Human	Bone	Ewing sarcoma
MIAPACA2	Human	Pancreas	Carcinoma
MM.1S	Human	Peripheral blood	Immunoglobulin A Lambda Myeloma
MOLT-4	Human	Hematological	Acute lymphoblastic leukemia ALL
MRC-5	Human	Lung	Normal cell line
MT3	Human	Breast	Adenocarcinoma
MV4-11	Human	Peripheral blood	Biphenotypic B myelomonocytic leukemia
NCI-ADR-RES	Human	Ovary	High grade ovarian serous adenocarcinoma
NCI-H1975	Human	Lung	Non-small cell lung cancer
NCI-H226	Human	Lung	Squamous cell carcinoma; mesothelioma
NCI-H23	Human	Lung	Non-small cell lung cancer
NCI-H292	Human	Lung	Carcinoma; mucoepidermoid pulmonary
NCI-H322M	Human	Lung	Minimally invasive lung adenocarcinoma
NCI-H358M	Human	Lung	Minimally invasive lung adenocarcinoma
NCI-H460	Human	Lung	Lung large cell carcinoma
NCI-H522	Human	Lung	Non-small cell lung cancer; Stage 2
Neuro-2a	Mouse	Brain	Neuroblastoma
OVCAR-3	Human	Ovary	High grade ovarian serous adenocarcinoma
OVCAR-4	Human	Ovary	High grade ovarian serous adenocarcinoma
OVCAR-5	Human	Ovary	High grade ovarian serous adenocarcinoma

OVCAR-8	Human	Ovary	High grade ovarian serous adenocarcinoma
OVXF-899	Human	Ovary	Ovarian adenocarcinoma
P388	Mouse		Lymphoma
PANC-1	Human	Pancreas; duct	Carcinoma
PANC-1005	Human	Pancreas	Adenocarcinoma
PAXF-1657	Human	Pancreas	Pancreas tumor cells
PBMC	Human	Hematological	Normal cell line
PC-3	Human	Prostate	Adenocarcinoma; Grade IV
PLCPRF5	Human	Liver	Hepatoma
PQ	Mouse		Normal cell line
RAW 264.7	Mouse	Hematological	Mouse leukemia
RBL-2H3	Rat	Peripheral blood	Basophilic leukemia
RD	Human	Muscle	Rhabdomyosarcoma
RDES	Human	Bone	Ewings Sarcoma
RPMI-8226	Human	Peripheral blood	Plasmacytoma
RXF 393	Human	Kidney	Renal cell carcinoma
RXF 486	Human	Kidney	Renal cell carcinoma
SAOS2	Human	Bone	Osteosarcoma
SF-268	Human	Brain	Neurocancer
SF-295	Human	Brain	Neurocancer
SF-539	Human	Brain	Neurocancer
SGC-7901	Human	Stomach	Gastric carcinoma
SH-SY5Y	Human	Bone marrow	Neuroblastoma
SKBR3	Human	Breast	Adenocarcinoma
SKLMS1	Human	Vulva	Leiomyosarcoma
SK-MEL-2	Human	Skin	Malignant Melanoma
SK-MEL-28	Human	Skin	Malignant Melanoma
SK-MEL-5	Human	Skin	Malignant Melanoma
SKNAS	Human	Brain	Neuroblastoma
SKNSH	Human	Brain	Neuroblastoma
SK-OV-3	Human	Ovary; Ascites	Adenocarcinoma
SMMC-7721	Human	Uterus; Cervix	HPV-related endocervical adenocarcinoma
SN12C	Human	Kidney	Renal cell carcinoma
SNB-19	Human	Brain; parietal lobe	Astrocytoma
SNB-75	Human	Brain	Glioma
SR	Human	Pleural effusion	Large Cell Immunoblastic Lymphoma
SU-DHL-6	Human	Hematological	Large Cell Lymphoma
SU-DHL-10	Human	Hematological	Large Cell Lymphoma
SUNE1	Human	Nasopharynx	Nasopharyngeal carcinoma
SW-620	Human	Large intestine; Colon	Adenocarcinoma; Colorectal; Dukes' type C
T24	Human	Bladder	Bladder carcinoma
T-47D	Human	Breast; Mammary gland	Carcinoma; Ductal
T98G	Human	Brian	Glioblastoma multiforme
TE671	Human	Muscle	Embryonal rhabdomyosarcoma

THP-1	Human	Hematological	Acute monocytic leukemia
TK-10	Human	Kidney	Clear cell renal cell carcinoma
U251	Human	Brain	Glioblastoma
U2OS	Human	Bone	Osteosarcoma
U87-MG	Human	Brain	Glioblastoma
UACC-257	Human		Melanoma
UACC-62	Human		Melanoma
UMUC-3	Human	Bladder	Bladder carcinoma
UO-31	Human	Kidney	Renal cell carcinoma
UXF-1138	Human	Uterus	Uterus cancer
XF498	Human		Glioblastoma

Table S2. Summary of the marine indole alkaloids isolated from marine microorganisms.

NO.	Strain	Source	Bioactivities	Country
**1**	*Streptomyces* sp.	Sediment, Yellow Sea, China, Rizhao,Shandong Province, China, summer of 2010, -50 to -100m.	Active: antimicrobial (fluconazole-resistant *C. albicans*)	China
**2**	*Acinetobacter* sp.	Mud, coastal area of Karachi, Sindh, Pakistan during September 2017.	Active: antimicrobial (methicillin-resistant *Staphylococcus aureus*, *Escherichia coli*, and *Candida albicans).*	China
**3**	*Acinetobacter* sp.	Mud, coastal area of Karachi, Sindh, Pakistan during September 2017.	Active: antimicrobial (methicillin-resistant *Staphylococcus aureus*, *Escherichia coli*, and *Candida albicans).*	China
**4**	*Streptomyces* sp.	Coastal soil.	Inactive: antimicrobial (MRSA and *E. coli).*	China
**5**	*Streptomyces* sp.	Coastal soil.	Inactive: antimicrobial (MRSA and *E. coli).*	China
**6**	*Streptomyces* sp.	Coastal soil.	Inactive: antimicrobial (MRSA and *E. coli).*	China
**7**	*Salinispora arenicola*	Sediment, Pernambuco state-Brazil, in November 2011, -16m (N 0°55′, W 29°38′).	Inactive: antimicrobial (*Enterococcus faecalis*, *Staphylococcus aureus* and *Escherichia coli*).	Brazil
**8**	*Salinispora arenicola*	Sediment, Pernambuco state-Brazil, in November 2011, -16m (N 0°55′, W 29°38′).	Inactive: antimicrobial (*Enterococcus faecalis*, *Staphylococcus aureus* and *Escherichia coli*).	Brazil
**9**	*Streptomyces* sp.	Sediment, South China Sea, -1,765 m.	Active: antimicrobial (*Micrococcus luteus* ML01, *Staphyloccocus aureus* and MRSA); cytotoxicity (MDA-MB-435, NCI-H460, MDA-MB-231, HCT-116, HepG2, and noncancerous MCF10A).	China
**10**	*Streptomyces* sp.	Sediment, South China Sea, -1,765 m.	Active: cytotoxicity (MDA-MB-435, NCI-H460, MDA-MB-231, HCT-116, HepG2, and noncancerous MCF10A).	China
**11**	*Streptomyces* sp.	Sediment, Dongtou, Zhejiang Province, China.	Active: inhibitory effect (Protein kinase C alpha, Rho-associated protein kinase and Apoptosis signal-regulating kinase); cytotoxicity (PC-3).	China
**12**	*Streptomyces* sp.	Sediment, Dongtou, Zhejiang Province, China.	Active: inhibitory effect (Protein kinase C alpha, Rho-associated protein kinase and Apoptosis signal-regulating	China

			kinase).<br>Inactive: cytotoxicity (PC-3).	
**13**	*Streptomyces* sp.	Sediment, Dongtou, Zhejiang Province, China.	Active: inhibitory effect (Protein kinase C alpha, Rho-associated protein kinase and Apoptosis signal-regulating kinase).<br>Inactive: cytotoxicity (PC-3).	China
**14**	*Streptomyces* sp.	Sediment, Dongtou, Zhejiang Province, China.	Active: inhibitory effect (Protein kinase C alpha, Rho-associated protein kinase and Apoptosis signal-regulating kinase).<br>Inactive: cytotoxicity (PC-3).	China
**15**	*Streptomyces* sp.	Sediment, Dongtou, Zhejiang Province, China.	Active: inhibitory effect (Protein kinase C alpha, Rho-associated protein kinase and Apoptosis signal-regulating kinase); cytotoxicity (PC-3).	China
**16**	*Streptomyces* sp.	Sediment, Dongtou, Zhejiang Province, China.	Active: inhibitory effect (Protein kinase C alpha, Rho-associated protein kinase and Apoptosis signal-regulating kinase); cytotoxicity (PC-3).	China
**17**	*Streptomyces* sp.	Sediment, Dongtou County, Zhejiang province, China, N27°49′04.44″ E121°07′55.33″.	Active: cytotoxicity (PC-3); inhibitory effect (PKCα, ROCK II and BTK).	China
**18**	*Streptomyces* sp.	Sediment, Dongtou County, Zhejiang province, China, N27°49′04.44″ E121°07′55.33″.	Active: cytotoxicity (PC-3); inhibitory effect (PKCα, ROCK II and BTK).	China
**19**	*Streptomyces* sp.	Sediment, Dongtou County, Zhejiang province, China, N27°49′04.44″ E121°07′55.33″.	Active: cytotoxicity (PC-3); inhibitory effect (PKCα, ROCK II and BTK).	China
**20**	*Streptomyces* sp.	Sediment, Dongtou, Zhejiang Province,	Active: cytotoxicity (PC-3); inhibitory effect (Protein kinase C	China

		China.	alpha, Rho-associated protein kinase and Apoptosis signal-regulating kinase).	
**21**	*Streptomyces* sp.	Sediment, Dongtou, Zhejiang Province, China.	Active: inhibitory effect (Protein kinase C alpha, Rho-associated protein kinase and Apoptosis signal-regulating kinase).<br>Inactive: cytotoxicity (PC-3)	China
**22**	*Streptomyces* sp.	Sediment, Dongtou, Zhejiang Province, China.	Active: inhibitory effect (Protein kinase C alpha, Rho-associated protein kinase and Apoptosis signal-regulating kinase); cytotoxicity (PC-3).	China
**23**	*Streptomyces* sp.	Sediment, Dongtou Island, Zhejiang province, China.	Inactive: Cytotoxicity (PC-3); inhibitory effect (Protein kinase C and Brution tyrosine kinase);.	China
**24**	*Streptomyces* sp.	Sediment, Dongtou Island, Zhejiang province, China.	Active: cytotoxicity (PC-3); inhibitory effect (Protein kinase C and Brution tyrosine kinase).	China
**25**	*Streptomyces* sp.	Sediment, Dongtou Island, Zhejiang province, China.	Active: cytotoxicity (PC-3); inhibitory effect (Protein kinase C and Brution tyrosine kinase).	China
**26**	*Streptomyces* sp.	Sediment, Ningbo County, Zhejiang province, China, N 29°28′ E 121°57′.	Active: inhibitory effect (Protein kinase C, Brution tyrosine kinase, Rho-associated protein kinase 2).	China
**27**	*Streptomyces* sp.	Sediment, the South China Sea, -2061 m.	Active: cytotoxicity (A549 and MCF-7).	China
**28**	*Streptomyces* sp.	Sediment, Lagos Lagoon, Okobaba, Offin, Folawiyo, Iddo, Ejirin, Imoru, Imope, Ikosi, Egbin, Ijede, Palaver Island, and Bayeku.	Active: Anticancer; cytotoxicity (HeLa).	Nigeria
**29**	*Escherichia coli*	Sediment, Southwestern Indian Ocean, -3006 m (102.612575°E, 2.022449°N).	Active: cytotoxicity (MCF7, B16, CNE2, Bel7402 and HT1080).	China
**30**	*Escherichia coli*	Sediment, Southwestern Indian Ocean, -3006 m (102.612575°E, 2.022449°N).	Inactive: cytotoxicity (MCF7, B16, CNE2, Bel7402 and HT1080).	China

**31**	*Nocardiopsis* sp.	Sediment, coast of Cô Tô – Quảng Ninh in Vietnam in June of 2014.	Active: cytotoxicity (KB and LU-1); antimicrobial (*E. faecalis, S. aureus, B. cereus, S. enterica, C. albicans*)	Vietnam
**32**	*Achromobacter spanius*	Sediment, California state beaches during 2012–2013.	Not detected.	USA
**33**	*Streptomyces* sp.	Sediment, Tautra ridge in the Trondheim fjord, Norway, -450m.	Not detected.	Germany
**34**	*Streptomyces* sp.	Sediment, Tautra ridge in the Trondheim fjord, Norway, -450m.	Not detected.	Germany
**35**	Saccharomonospora sp.	Sediment, 2km west of the Scripps pier, in La Jolla, CA, USA.	Active: antimicrobial (methicillin-resistant Staphylococcus aureus and vancomycin-resistant Enterococcus faecium).	USA
**36**	Streptomyces xinghaiensis	Sediment, Xinghai Bay in Dalian, China.	Not detected.	China
**37**	Streptomyces xinghaiensis	Sediment, Xinghai Bay in Dalian, China.	Not detected.	China
**38**	Streptomyces xinghaiensis	Sediment, Xinghai Bay in Dalian, China.	Not detected.	China
**39**	*Rubrobacter radiotolerans*	Sponge *Petrosia* sp.	Active: activity on acetylcholinesterase (AchE); antichlamydial; Inactive: cytoxicity (HCT115, DU-145, PC-3, XF498, and HT29 human solid tumor cell lines).	China
**40**	*Rubrobacter radiotolerans*	Sponge *Petrosia* sp.	Active: activity on acetylcholinesterase (AchE); antichlamydial; Inactive: cytoxicity (HCT115, DU-145, PC-3, XF498, and HT29 human solid tumor cell lines).	China
**41**	*Rubrobacter radiotolerans*	Sponge Petrosia sp., off the coast of Xisha Islands, China,-15m to -25m, in 2013.	Active: antichlamydial.	China
**42**	*Rhodococcus* sp.	Sponge *Callyspongia* aff. *Implexa* (family Callyspongiidae), Red Sea, Ras Mohamed,	Active: antibacterial (*Staphylococcus aureus* NCTC 8325); antitrypanosomal(*Trypanosoma brucei brucei* TC221).	Egypt

		Sinai, Egyp, -10 m.		
**43**	*Streptomyces* sp.	Sponge, NaoZhou Island of the Guangdong Province, China.	Active: anti-influenza (influenza A H1N1 virus).	China
**44**	*Lysinibacillus fusiformis*	Sponge, Kanagawa prefecture, Japan.	Inactive:cytotoxicity (P388), hemolytic and brine shrimp lethal activity.	Japan
**45**	*Saccharomonospora* sp.<br>*Dietzia* sp.	Sponge, Red Sea, Hurghada, Egypt, in November 2015, -10 m.	Active: inhibitory effect (Pim-1 kinase); antiproliferative(HT-29 and HL-60).	Egypt
**46**	*Saccharomonospora* sp.<br>*Dietzia* sp.	Sponge, Red Sea, Hurghada, Egypt, in November 2015, -10 m.	Inactive: inhibitory effect (Pim-1 kinase); antiproliferative (H1650, HL-60 and HT-29).	Egypt
**47**	*Streptomyces* sp.	Sponge, on the beach 200 m south of Scripps Institution of Oceanography Pier in La Jolla, CA, December 2016.	Active: cytotoxicity (U87 and SKOV3).	USA
**48**	*Streptomyces* sp.	Sponge, on the beach 200 m south of Scripps Institution of Oceanography Pier in La Jolla, CA, December 2016.	Active: cytotoxicity (U87 and SKOV3).	USA
**49**	*Streptomyces* sp.	Sponge, on the beach 200 m south of Scripps Institution of Oceanography Pier in La Jolla, CA, December 2016.	Active: cytotoxicity (U87 and SKOV3).	USA
**50**	*Streptomyces* sp.	Sponge, on the beach 200 m south of Scripps Institution of Oceanography Pier in La Jolla, CA, December 2016.	Active: cytotoxicity (U87 and SKOV3).	USA
**51**	*Streptomyces* sp.	South China Sea	Active: activities on nuclear factor erythroid 2-related factor 2 (Nrf2) transcription in HepG2 cells.	China
**52**	Recombinant	No source information reported.	Active: antimicrobial (*Bacillus subtilis, Staphylococcus aureus,*	Germany

	Streptomyces sp.		*Mycobacterium vaccae, Staphylococus aureus,* and *Enterococcus faecalis*).<br>Inactive: cytotoxicity (L-929, K562, HeLa).	
**53**	Recombinant *Streptomyces* sp.	No source information reported.	Active: antimicrobial (*Staphylococcus aureus, Mycobacterium vaccae,* and *Enterococcus faecalis*).<br>Inactive: antimicrobial (*Bacillus subtilis, Staphylococus aureus* >100µg mL^{-1}); cytotoxicity (L-929, K562, HeLa).	Germany
**54**	Recombinant *Streptomyces* sp.	No source information reported.	Active: antimicrobial (*Bacillus subtilis, Staphylococcus aureus, Mycobacterium vaccae, Staphylococus aureus,* and *Enterococcus faecalis*).<br>Inactive: cytotoxicity (L-929, K562, HeLa).	Germany
**55**	*Vibrio splendidus*	Gastrointestinal tract of a small fish dredged ,South Orkney Islands near Antarctica.	Active: against the peronosporomycetes *Botrytis cinerea* and *Phytophthora infestans*; cytotoxicity (HT-29, GXF 251, LXFL 529, LXFA 629, MAXF 401, MEXF 462, OVXF 899, PAXF 1657, 22Rv1, RXF 486, UXF 1138).	Germany
**56**	*Vibrio splendidus*	Gastrointestinal tract of a small fish dredged ,South Orkney Islands near Antarctica.	Active: antimicrobial (*B. subtilis*, *S. viridochromogenes*, and *E. coli*).	Germany
**57**	*Vibrio splendidus*	Gastrointestinal tract of a small fish dredged ,South Orkney Islands near Antarctica.	Active: antimicrobial (*B. subtilis*, *S. viridochromogenes*, and *E. coli*).	Germany
**58**	*Vibrio splendidus*	Gastrointestinal tract of a small fish dredged ,South Orkney Islands near Antarctica.	Active: antimicrobial (*B. subtilis*, *S. viridochromogenes*, and *E. coli*).	Germany
**59**	*Vibrio splendidus*	Gastrointestinal tract of a small fish dredged ,South Orkney Islands near	Active: antimicrobial (*Bacillus subtilis, Staphylococcus aureus, Escherichia coli, Candida albicans, Mucor miehei, Pythium*	Germany

		Antarctica.	*ultimum*).	
**60**	*Vibrio splendidus*	Gastrointestinal tract of a small fish dredged ,South Orkney Islands near Antarctica.	Active: antimicrobial (*B. subtilis*, *S. aureus*, *S. viridochromogenes*, *E. coli*, *C. albicans*, and *M. miehei*).	Germany
**61**	*Arthrobacter psychrochitiniphilus*	Excrement of penguins, Polish base Arctowski on King George Island.	Not detected.	Germany
**62**	*Pseudoalteromonas rubra*	Seawater, Qingdao Sea area in China.	Active: showed a weak 5-HT2A receptor antagonist.	China
**63**	*Bacillus subterraneus*	The South China Sea (−2918m, 119°19.896′, 19°41.569′).	Inactive: anti-allergic.	China
**64**	*Pseudovibrio denitrificans*	Sea water.	Active: cytotoxicity (L929 and A549).	France
**65**	*Penicllium commune*	Sediment, South China Sea, Sansha City, Hainan Province, China, -3563m.	Active: displayed lethal activity on brine shrimp.<br>Inactive: cytotoxicity(HepG-2 and HeLa), anti-influenza (H1N1).	China
**66**	*Penicllium commune*	Sediment, South China Sea, Sansha City, Hainan Province, China, -3563m.	Active: displayed lethal activity on brine shrimp.<br>Inactive: cytotoxicity(HepG-2 and HeLa), anti-influenza (H1N1).	China
**67**	*Penicllium commune*	Sediment, South China Sea, Sansha City, Hainan Province, China, -3563m.	Active: displayed lethal activity on brine shrimp.<br>Inactive: cytotoxicity(HepG-2 and HeLa), anti-influenza (H1N1).	China
**68**	*Penicllium commune*	Sediment, South China Sea, Sansha City, Hainan Province, China, -3563m.	Active: displayed lethal activity on brine shrimp.<br>Inactive: cytotoxicity(HepG-2 and HeLa), anti-influenza (H1N1).	China
**69**	*Penicllium commune*	Sediment, South China Sea, Sansha City, Hainan Province, China, -3563m.	Active: displayed lethal activity on brine shrimp.<br>Inactive: cytotoxicity(HepG-2 and HeLa), anti-influenza	China

			(H1N1).	
**70**	*Penicllium commune*	Sediment, South China Sea, Sansha City, Hainan Province, China, -3563m.	Active: displayed lethal activity on brine shrimp.<br>Inactive: cytotoxicity(HepG-2 and HeLa), anti-influenza (H1N1).	China
**71**	*Penicllium commune*	Sediment, South China Sea, Sansha City, Hainan Province, China, -3563m.	Active: displayed lethal activity on brine shrimp.<br>Inactive: cytotoxicity(HepG-2 and HeLa), anti-influenza (H1N1).	China
**72**	*Penicllium commune*	Sediment, South China Sea, Sansha City, Hainan Province, China, -3563m.	Active: displayed lethal activity on brine shrimp.<br>Inactive: cytotoxicity(HepG-2 and HeLa), anti-influenza (H1N1).	China
**73**	*Penicllium commune*	Sediment, South China Sea, Sansha City, Hainan Province, China, -3563m.	Active: displayed lethal activity on brine shrimp.<br>Inactive: cytotoxicity(HepG-2 and HeLa), anti-influenza (H1N1).	China
**74**	*Penicillium citrinum*	Sediment, South China Sea, -1500 m.	Not detected.	China
**75**	*Penicillium citrinum*	Sediment, South China Sea, -1500 m.	Not detected.	China
**76**	*Penicillium purpurogenum*	Mud, luju River intertidal zone, Bohai Bay, Tanggu, Tianjin	Active: cytotoxicity (K562, HL-60, HeLa and BGC-823).	China
**77**	*Penicillium purpurogenum*	Mud, luju River intertidal zone, Bohai Bay, Tanggu, Tianjin	Active: cytotoxicity (K562, HL-60, HeLa and BGC-823).	China
**78**	*Penicillium purpurogenum*	Mud, luju River intertidal zone, Bohai Bay, Tanggu, Tianjin	Active: cytotoxicity (K562, HL-60, HeLa and BGC-823).	China
**79**	*Penicillium purpurogenum*	Mud, luju River intertidal zone, Bohai Bay, Tanggu, Tianjin	Active: cytotoxicity (K562, HL-60, HeLa and BGC-823).	China
**80**	*Penicillium*	Mud, luju River intertidal zone, Bohai Bay,	Active: cytotoxicity (K562, HL-60, HeLa and BGC-823).	China

	purpurogenum	Tanggu, Tianjin		
**81**	*Penicillium purpurogenum*	Mud, luju River intertidal zone, Bohai Bay, Tanggu, Tianjin	Active: cytotoxicity (K562, HL-60, HeLa and BGC-823).	China
**82**	*Aspergillus versicolor.*	Mud, South China Sea.	Inactive: anti-inflammatory.	China
**83**	*Aspergillus versicolor.*	Mud, South China Sea.	Active: iNOS inhibitory activities; inhibited the release of NO in LPS-induced Raw264.7 cells; anti-inflammatory.	China
**84**	*Aspergillus versicolor.*	Mud, South China Sea.	Active: iNOS inhibitory activities; inhibited the release of NO in LPS-induced Raw264.7 cells; anti-inflammatory.	China
**85**	*Aspergillus versicolor.*	Mud, South China Sea.	Inactive: anti-inflammatory.	China
**86**	*Aspergillus versicolor.*	Mud, South China Sea.	Active: iNOS inhibitory activities; inhibited the release of NO in LPS-induced Raw264.7 cells; anti-inflammatory.	China
**87**	*Aspergillus versicolor.*	Mud, South China Sea.	Inactive: anti-inflammatory activities.	China
**88**	*Aspergillus versicolor.*	Mud, South China Sea.	Active: anti-inflammatory; inhibitory effect（iNOS）.	China
**89**	*Aspergillus versicolor.*	Mud, South China Sea.	Inactive: anti-inflammatory.	China
**90**	*Aspergillus versicolor.*	Mud, South China Sea.	Not detected.	China
**91**	*Aspergillus versicolor*	Sediment, Bohai Sea, China.	Inactive: antimicrobial (Bacillus Calmette–Guérin, *Staphylococcus aureus*, methicillin-resistant *S. aureus*, *Bacillus subtilis*, *Pseudomonas aeruginosa* and *Candida albicans*).	China
**92**	*Aspergillus versicolor*	Sediment, Bohai Sea, China.	Inactive: antimicrobial (Bacillus Calmette–Guérin, *Staphylococcus aureus*, methicillin-resistant *S. aureus*, *Bacillus*	China

			subtilis, Pseudomonas aeruginosa and *Candida albicans*).	
**93**	*Aspergillus versicolor*	Sediment, Bohai Sea, China.	Inactive: antimicrobial (Bacillus Calmette–Guérin, *Staphylococcus aureus*, methicillin-resistant *S. aureus*, *Bacillus subtilis, Pseudomonas aeruginosa* and *Candida albicans*).	China
**94**	*Aspergillus versicolor*	Sediment, Bohai Sea, China.	Inactive: antimicrobial (Bacillus Calmette–Guérin, *Staphylococcus aureus*, methicillin-resistant *S. aureus*, *Bacillus subtilis, Pseudomonas aeruginosa* and *Candida albicans*).	China
**95**	*Aspergillus versicolor*	Sediment, Bohai Sea, China.	Active: antimicrobial (*S. aureus* and methicillin-resistant *S. aureus*).	China
**96**	*Aspergillus versicolor*	Sediment, Bohai Sea, China.	Active: antimicrobial (*S. aureus* and methicillin-resistant *S. aureus*).	China
**97**	*Aspergillus versicolor*	Sediment, Bohai Sea, China.	Not detected.	China
**98**	*Aspergillus versicolor*	Sediment, Bohai Sea, China.	Not detected.	China
**99**	*Penicillium granulatum*	Sediment, -2284 m.	Active: cytotoxicity (HepG2).	China
**100**	*Acremonium persicinum*	Sediment, South China Sea.	Not detected.	Chian
**101**	*Acremonium persicinum*	Sediment, South China Sea.	Active: antiviral (herpes simplex virus 1).	China
**102**	*Chaetomium globosum.*	Sediment, Indian Ocean.	Active: antiproliferative (LNCaP and B16F10).	China
**103**	*Chaetomium globosum.*	Sediment, Indian Ocean.	Inactive: cytotoxicity (MDA-MB-231, LNCaP and B16F10).	China
**104**	*Aspergillus*	Sediment, the Indian Ocean, in 2013, -3614	Active: antifungal (*Fusarium oxysporum* f. sp. *momordicae).*	China

	fumigatus	m.	Inactive: antibacterial (*S. aureus*, *A. baumanii* and *K. pneumonia*).	
**105**	*Aspergillus fumigatus*	Sediment, the Indian Ocean, in 2013, -3614 m.	Active: antibacterial (*A. baumanii*).<br>Inactive: antifungal (*Fusarium oxysporum* f.sp. *cucumerinu* and *Fusarium oxysporum* f. sp. *momordicae*).	China
**106**	*Penicillium janthinellum*	Sediment, Bohai Sea, in June 2016.	Active: anti-Vibrio (*Vibrio anguillarum*, *Vibrio parahemolyticus* and *Vibrio alginolyticus*).	China
**107**	*Penicillium janthinellum*	Sediment, Bohai Sea, in June 2016.	Active: anti-Vibrio (*Vibrio anguillarum*, *Vibrio parahemolyticus* and *Vibrio alginolyticus*).	China
**108**	*Aspergillus fumigatus*	Sediment, Bohai Sea, China, -60 m.	Not detected.	Australia
**109**	*Aspergillus fumigatus*	Sediment, Bohai Sea, China, -60 m.	Not detected.	Australia
**110**	*Aspergillus* sp.	Sediment, Indian Ocean, in April 2016 (0° 0.379′S, 87° 10.467′E; -4507 m).	Inactive: NO production inhibitory; anti-inflammatory.	China
**111**	*Aspergillus* sp.	Sediment, Indian Ocean, in April 2016 (0° 0.379′S, 87° 10.467′E; -4507 m).	Inactive: NO production inhibitory; anti-inflammatory.	China
**112**	*Aspergillus* sp.	Sediment, Indian Ocean, in April 2016 (0° 0.379′S, 87° 10.467′E; -4507 m).	Inactive: NO production inhibitory; anti-inflammatory.	China
**113**	*Aspergillus* sp.	Sediment, Indian Ocean, in April 2016 (0° 0.379′S, 87° 10.467′E; -4507 m).	Active: inhibitory activities against NO production.	China
**114**	*Eurotium* sp.	Sediment, South China Sea, in May 2010, -158 m.	Inactive: cytotoxicity (SF-268 and HepG2); antioxidative.	China
**115**	*Eurotium* sp.	Sediment, South China Sea, in May 2010, -158 m.	Inactive: cytotoxicity (SF-268 and HepG2); antioxidative.	China
**116**	*Eurotium* sp.	Sediment, South China Sea, in May 2010,-158 m.	Active: showed significant radical scavenging activities against DPPH	China

**117**	*Eurotium* sp.	Sediment, South China Sea, in May 2010,-158 m.	Inactive: cytotoxicity (SF-268 and HepG2); antioxidative.	China
**118**	*Eurotium* sp.	Sediment, South China Sea, in May 2010,-158 m.	Active: showed significant radical scavenging activities against DPPH.<br>Inactive: cytotoxicity (SF-268 and HepG2).	China
**119**	*Eurotium* sp.	Sediment, South China Sea, in May 2010,-158 m.	Active: showed significant radical scavenging activities against DPPH.<br>Inactive: cytotoxicity (SF-268 and HepG2).	China
**120**	*Eurotium* sp.	Sediment, South China Sea, in May 2010,-158 m.	Active: showed significant radical scavenging activities against DPPH.<br>Inactive: cytotoxicity (SF-268 and HepG2).	China
**121**	*Eurotium* sp.	Sediment, South China Sea, in May 2010,-158 m.	Active: showed significant radical scavenging activities against DPPH.<br>Inactive: cytotoxicity (SF-268 and HepG2).	China
**122**	*Eurotium* sp.	Sediment, South China Sea, in May 2010,-158 m.	Active: showed significant radical scavenging activities against DPPH.<br>Inactive: cytotoxicity (SF-268 and HepG2).	China
**123**	*Eurotium* sp.	Sediment, South China Sea, in May 2010,-158 m.	Active: showed significant radical scavenging activities against DPPH.<br>Inactive: cytotoxicity (SF-268 and HepG2 cell lines).	China
**124**	*Eurotium* sp.	Sediment, South China Sea.	Active: antioxidative (DPPH).<br>Inactive: cytotoxicity (SF-268 and HepG2).	China
**125**	*Eurotium* sp.	Sediment, South China Sea.	Inactive: antioxidative (DPPH); cytotoxicity (SF-268 and HepG2).	China
**126**	*Eurotium* sp.	Sediment, South China Sea.	Inactive: antioxidative (DPPH).<br>Active: cytotoxicity (SF-268 and HepG2).	China

**127**	*Eurotium* sp.	Sediment, South China Sea.	Inactive: antioxidative (DPPH); cytotoxicity (SF-268 and HepG2).	China
**128**	*Eurotium* sp.	Sediment, South China Sea.	Inactive: antioxidative (DPPH).<br>Active: cytotoxicity (SF-268 and HepG2).	China
**129**	*Eurotium* sp.	Sediment, South China Sea.	Inactive: antioxidative (DPPH); cytotoxicity (SF-268 and HepG2).	China
**130**	*Graphium* sp.	Sediment, Ishigaki Island, Okinawa, Japan, -17m.	Active: inhibited yellow pigment production by methicillin-resistant *Staphylococcus aureus* (MRSA).	Japan
**131**	*Graphium* sp.	Sediment, Ishigaki Island, Okinawa, Japan, -17m.	Active: inhibited yellow pigment production by methicillin-resistant *Staphylococcus aureus* (MRSA).	Japan
**132**	*Dichotomomyces cejpii*	Sediment, South China Sea, -3941 m.	Active: inhibitory effect (α-glucosidase).<br>Inactive: cytotoxicity (SF-268, MCF-7, NCI-H460, and HepG-2).	China
**133**	*Dichotomomyces cejpii*	Sediment, South China Sea, -3941 m.	Inactive: cytotoxicity (SF-268, MCF-7, NCI-H460, and HepG-2); inhibitory effect (α-glucosidase).	China
**134**	*Chaetomium cristatum*	Sediment, Suncheon Bay, Korea.	Active: displayed potent radical-scavenging activity against DPPH; cytotoxicity (HeLa).	South Korea
**135**	*Acrostalagmus luteoalbus*	Soil, Liaodong Bay, China.	Active: cytotoxicity (A549, HCT-116, K562, H1975 and HL-60).	China
**136**	*Acrostalagmus luteoalbus*	Soil, Liaodong Bay, China.	Active: cytotoxicity (A549, HCT-116, K562, H1975 and HL-60).	Chian
**137**	*Aspergillus fumigatus*	Sediment, Red Sea, Hurghada, Egypt in September 2011	Not detected.	UK
**138**	*1.Aspergillus sulphureus*<br>*2.Isaria felina*	1.（KMM 4640）sediment.<br>2.（KMM 4639） sediment, South China Sea, coast of Vietnam, -10 m.	Not detected.	Russia
**139**	*1.Aspergillus*	1.（KMM 4640）sediment.	Active: cytotoxicity (22Rv1).	Russia

	sulphureus<br>2.*Isaria felina*	2.（KMM 4639） sediment, South China Sea, coast of Vietnam, -10 m.		
**140**	1.*Aspergillus sulphureus*<br>2.*Isaria felina*	1.（KMM 4640）sediment.<br>2.（KMM 4639） sediment, South China Sea, coast of Vietnam, -10 m.	Not detected.	Russia
**141**	1.*Aspergillus sulphureus*<br>2.*Isaria felina*	1.（KMM 4640）sediment.<br>2.（KMM 4639） sediment, South China Sea, coast of Vietnam, -10 m.	Not detected.	Russia
**142**	1.*Aspergillus sulphureus*<br>2.*Isaria felina*	1.（KMM 4640）sediment.<br>2.（KMM 4639） sediment, South China Sea, coast of Vietnam, -10 m.	Not detected.	Russia
**143**	*Penicillium brevicompactum*	Sediment, South China Sea, Sansha City, Hainan Province(18°5′N, 118°31′E; -3928 m).	Inactive: cytotoxicity (HCT116); antimicrobial (*Streptococcus mutans*, *S. sobrinus* and *Fusarium oxysporum* f. sp. *cubense* Race 1 and Race 4); antilarval.	China
**144**	*Penicillium brevicompactum*	Sediment, South China Sea, Sansha City, Hainan Province(18°5′N, 118°31′E; -3928 m).	Inactive: cytotoxicity (HCT116); antimicrobial (*Streptococcus mutans*, *S. sobrinus* and *Fusarium oxysporum* f. sp. *cubense* Race 1 and Race 4).	China
**145**	*Aspergillus* sp.	Soil, the intertidal zone of Zhoushan, Zhejiang, China, in June 2018.	Active: antimicrobial (*Vibrio anguillarum*, *Xanthomonas oryzae pv. Oryzicola*, and *Rhizoctonia solan*).<br>Inactive: anti-inflammatorym (*Propionibacterium acnes*).	China
**146**	*Aspergillus* sp.	Soil, the intertidal zone of Zhoushan, Zhejiang, China, in June 2018.	Not detected.	China
**147**	*Aspergillus* sp.	Soil, the intertidal zone of Zhoushan, Zhejiang, China, in June 2018.	Not detected.	China
**148**	*Aspergillus* sp.	Soil, the intertidal zone of Zhoushan, Zhejiang, China, in June 2018.	Not detected.	China

**149**	*Aspergillus* sp.	Soil, the intertidal zone of Zhoushan, Zhejiang, China, in June 2018.	Active: antifungal (*R. solani.*); anti-inflammatory (*P.acnes*-induced THP-1 cells).	China
**150**	*Aspergillus* sp.	Soil, the intertidal zone of Zhoushan, Zhejiang, China, in June 2018.	Active: anti-inflammatory (*P.acnes*-induced THP-1 cells).	China
**151**	*Penicillium* sp.	Sediment, mangrove swamp in Sanya, Hainan province, China.	Active: antiviral (HIV-1 and HCV); antibacterial (*Helicobacter pylori*).<br>Inactive: antibacterial (*Staphylococcus aureus*, *Bacillus subtilis*, *Pseudomonas aeruginosa* and *Klebsiella pneumonia*).	China
**152**	*Penicillium* sp.	Mangrove sediment, Sanya, in August 2010(18°13′50.2″N, 109°37′ 15.8″E).	Inactive: anti-allergic (RBL-2H3).	China
**153**	*Penicillium* sp.	Mangrove sediment, Sanya, in August 2010(18°13′50.2″N, 109°37′ 15.8″E).	Inactive: anti-allergic (RBL-2H3).	China
**154**	*Penicillium* sp.	Mangrove sediment, Sanya, in August 2010(18°13′50.2″N, 109°37′ 15.8″E).	Inactive: anti-allergic (RBL-2H3).	China
**155**	*Penicillium* sp.	Mangrove sediment, Sanya, in August 2010(18°13′50.2″N, 109°37′ 15.8″E).	Inactive: anti-allergic (RBL-2H3).	China
**156**	*Penicillium janthi-nellum*	Mangrove rhizosphere soil, Dongzhaigang mangrove natural reserve, Hainan Island, in September 2015.	Inactive: antibacterial (*Staphylococcus aureus*, *Enterococcus faecalis* and *Escherichia coli*); inhibitory effect (topoisomerase I); lethality towards brine shrimp *Artemia salina.*	China
**157**	*Penicillium raistrickii*	Sediment, mangrove swamp in Sanya, Hainan Province, China.	Active: anti-HCV (hepatitis C virus).	China
**158**	*Dichotomomyces cejpii*	Soft coral *Lobophytum crassum*, Hainan Sanya National Coral Reef Reserve, China.	Active: antimicrobial (*Staphylococcus aureus*, *Escherichia coli*, *Pseudomonas aeruginosa*, and *Bauman′s acinetobacter*).	China
**159**	*Dichotomomyces cejpii*	Soft coral *Lobophytum crassum*, Hainan Sanya National Coral Reef Reserve, China.	Inactive: cytotoxicity (HCT116, RD, ACHN, and A2780T).	China
**160**	*Scedosporium*	Soft coral *Lobophytum crassum*, Hainan Sanya	Inactive: antihepatitis C virus (HCV).	China

	apiospermum	National Coral Reef Reserve, People's Republic of China.		
**161**	*Scedosporium apiospermum*	Soft coral *Lobophytum crassum*, Hainan Sanya National Coral Reef Reserve, People's Republic of China.	Inactive: cytotoxicity (A2780T, Calu3, HepG2, MDA-MB-435, ACHN and RD); antihepatitis C virus (HCV).	China
**162**	*Scedosporium apiospermum*	Soft coral *Lobophytum crassum*, Hainan Sanya National Coral Reef Reserve, People's Republic of China.	Not detected.	China
**163**	*Scedosporium apiospermum*	Soft coral *Lobophytum crassum*, Hainan Sanya National Coral Reef Reserve, People's Republic of China.	Inactive: cytotoxicity (A2780T, Calu3, HepG2, MDA-MB-435, ACHN and RD); antihepatitis C virus (HCV).	China
**164**	*Scedosporium apiospermum*	Soft coral *Lobophytum crassum*, Hainan Sanya National Coral Reef Reserve, People's Republic of China.	Not detected.	China
**165**	*Scedosporium apiospermum*	Soft coral *Lobophytum crassum*, Hainan Sanya National Coral Reef Reserve, People's Republic of China.	Active: anti-HCV against the J8CC recombinant.<br>Inactive: cytotoxicity.	China
**166**	*Scedosporium apiospermum*	Soft coral *Lobophytum crassum*, Hainan Sanya National Coral Reef Reserve, People's Republic of China.	Inactive: cytotoxicity.	China
**167**	*Scedosporium apiospermum*	Soft coral *Lobophytum crassum*, Hainan Sanya National Coral Reef Reserve, People's Republic of China.	Not detected.	China
**168**	*Scedosporium apiospermum*	Soft coral *Lobophytum crassum*, Hainan Sanya National Coral Reef Reserve, People's Republic of China.	Active: anti-HCV against the J8CC recombinant.<br>Inactive: cytotoxicity (A2780T, Calu3, HepG2, MDA-MB-435, ACHN and RD).	China

**169**	*Scedosporium apiospermum*	Soft coral *Lobophytum crassum*, Hainan Sanya National Coral Reef Reserve, People's Republic of China.	Inactive: antihepatitis C virus (HCV) activity.	China
**170**	*Scedosporium apiospermum*	Soft coral *Lobophytum crassum*, Hainan Sanya National Coral Reef Reserve, People's Republic of China.	Not detected.	China
**171**	*Scedosporium apiospermum*	Soft coral *Lobophytum crassum*, Hainan Sanya National Coral Reef Reserve, People's Republic of China.	Inactive: antihepatitis C virus (HCV) activity.	China
**172**	*Scedosporium apiospermum*	Soft coral *Lobophytum crassum*, Hainan Sanya National Coral Reef Reserve, People's Republic of China.	Not detected.	China
**173**	*Scedosporium apiospermum*	Soft coral *Lobophytum crassum*, Hainan Sanya National Coral Reef Reserve, People's Republic of China.	Not detected.	China
**174**	*Scedosporium apiospermum*	Soft coral *Lobophytum crassum*, Hainan Sanya National Coral Reef Reserve, People's Republic of China.	Active: promote triglyceride accumulation in 3T3-L1 cells.	China
**175**	*Pseudallescheria boydii*	Soft coral *Sarcophyton* sp., Hainan Sanya National Coral Reef Reserve, China.	Inactive: cytotoxicity (A549, GLC82, CNE1, CNE2, HONE1 SUNE1, BEL7402 and SMMC7721).	China
**176**	*Pseudallescheria boydii*	Soft coral *Sarcophyton* sp., Hainan Sanya National Coral Reef Reserve, China.	Inactive: cytotoxicity (A549, GLC82, CNE1, CNE2, HONE1 SUNE1, BEL7402 and SMMC7721).	China
**177**	*Pseudallescheria ellipsoidea*	Soft coral *Lobophytum crassum* Hainan Sanya National Coral Reef Reserve, China.	Not detected.	China
**178**	*Pseudallescheria ellipsoidea*	Soft coral *Lobophytum crassum* Hainan Sanya National Coral Reef Reserve, China.	Not detected.	China

**179**	*Pseudallescheria ellipsoidea*	Soft coral *Lobophytum crassum* Hainan Sanya National Coral Reef Reserve, China.	Not detected.	China
**180**	*Pseudallescheria ellipsoidea*	Soft coral *Lobophytum crassum* Hainan Sanya National Coral Reef Reserve, China.	Not detected.	China
**181**	*Pseudallescheria boydii*	Soft coral *Lobophytum crassum* Hainan Sanya National Coral Reef Reserve, China.	Not detected.	China
**182**	*Chaetomium globosum*	Coral *Pocillopora damicornis*, seashore near Sanya Bay, Hainan Province, China, in March 2018.	Not detected.	China
**183**	*Aspergillus* sp.	Soft coral, South China Sea.	Inactive: cytotoxicity (NCI-H1975/GR, 50 μM).	China
**184**	*Aspergillus* sp.	Soft coral, South China Sea.	Inactive: cytotoxicity (NCI-H1975/GR, 50 μM).	China
**185**	*Aspergillus* sp.	Soft coral, South China Sea.	Inactive: cytotoxicity (NCI-H1975/GR, 50 μM).	China
**186**	*Penicillium dimorphosporum*	Soft coral, South China Sea.	Inactive: Neuroprotective activity (Paraquat-Induced Neurotoxicity on Neuro-2a).	Russia
**187**	*Penicillium dimorphosporum*	Soft coral, South China Sea.	Inactive: Neuroprotective activity (Paraquat-Induced Neurotoxicity on Neuro-2a).	Russia
**188**	*Penicillium dimorphosporum*	Soft coral, South China Sea.	Inactive: Neuroprotective activity (Paraquat-Induced Neurotoxicity on Neuro-2a).	Russia
**189**	*Penicillium dimorphosporum*	Soft coral, South China Sea.	Active: Neuroprotective activity (Paraquat-Induced Neurotoxicity on Neuro-2a).	Russia
**190**	*Penicillium dimorphosporum*	Soft coral, South China Sea.	Active: Neuroprotective activity (Paraquat-Induced Neurotoxicity on Neuro-2a).	Russia
**191**	*Penicillium dimorphosporum*	Soft coral, South China Sea.	Active: Neuroprotective activity (Paraquat-Induced Neurotoxicity on Neuro-2a).	Russia
**192**	*Penicillium dimorphosporum*	Soft coral, South China Sea.	Inactive: Neuroprotective activity (Paraquat-Induced Neurotoxicity on Neuro-2a).	Russia

**193**	*Aspergillus versicolor*	Gorgonian *Pseudopterogorgia* sp., South China Sea, in May 2015.	Active: exhibited inhibitory activities against thioredoxin reductase.<br>Inactive: cytotoxicity (A549 and A2780).	China
**194**	*Aspergillus versicolor*	Gorgonian *Pseudopterogorgia* sp. South China Sea, in May 2015.	Active: exhibited inhibitory activities against thioredoxin reductase.<br>Inactive: cytotoxicity (A549 and A2780).	China
**195**	*Aspergillus versicolor*	Gorgonian *Pseudopterogorgia* sp. South China Sea, in May 2015.	Inactive: cytotoxicity (A549 and A2780).	China
**196**	*Aspergillus versicolor*	Gorgonian *Pseudopterogorgia* sp. South China Sea, in May 2015.	Inactive: cytotoxicity (A549 and A2780).	China
**197**	*Aspergillus versicolor*	Gorgonian *Pseudopterogorgia* sp. South China Sea, in May 2015.	Inactive: cytotoxicity (A549 and A2780).	China
**198**	*Aspergillus versicolor*	Gorgonian *Pseudopterogorgia* sp. South China Sea, in May 2015.	Inactive: cytotoxicity (A549 and A2780).	China
**199**	*Aspergillus versicolor*	Gorgonian *Pseudopterogorgia* sp. South China Sea, in May 2015.	Active: inhibitory effect (thioredoxin reductase).<br>Inactive: cytotoxicity (A549 and A2780).	China
**200**	*Aspergillus versicolor*	Gorgonian *Pseudopterogorgia* sp. South China Sea, in May 2015.	Inactive: cytotoxicity (A549 and A2780).	China
**201**	*Aspergillus versicolor*	Gorgonian *Pseudopterogorgia* sp. South China Sea, in May 2015.	Inactive: cytotoxicity (A549 and A2780).	China
**202**	*Aspergillus versicolor*	Gorgonian *Pseudopterogorgia* sp. South China Sea, in May 2015.	Inactive: cytotoxicity (A549 and A2780).	China
**203**	*Aspergillus versicolor*	Gorgonian *Pseudopterogorgia* sp. South China Sea, in May 2015.	Active: inhibitory effect (thioredoxin reductase).<br>Inactive: cytotoxicity (A549 and A2780).	China
**204**	*Aspergillus versicolor*	Gorgonian *Pseudopterogorgia* sp. South China Sea, in May 2015.	Active: cytotoxicity (A549).	China

**205**	*Aspergillus versicolor*	Gorgonian *Pseudopterogorgia* sp. South China Sea, in May 2015.	Active: cytotoxicity (A549).	China
**206**	*Aspergillus versicolor*	Gorgonian *Pseudopterogorgia* sp. South China Sea, in May 2015.	Active: cytotoxicity (A549).	China
**207**	*Aspergillus versicolor*	Gorgonian *Pseudopterogorgia* sp. South China Sea, in May 2015.	Active: cytotoxicity (A549).	China
**208**	*Aspergillus versicolor*	Gorgonian *Pseudopterogorgia* sp. South China Sea, in May 2015.	Active: cytotoxicity (A549); inhibitory effect (thioredoxin reductase).	China
**209**	*Aspergillus versicolor*	Gorgonian *Pseudopterogorgia* sp. South China Sea, in May 2015.	Active: cytotoxicity (A549); inhibitory effect (thioredoxin reductase).	China
**210**	*Aspergillus versicolor*	Gorgonian *Pseudopterogorgia* sp. South China Sea, in May 2015.	Inactive: cytotoxicity (A549 and A2780).	China
**211**	*Aspergillus versicolor*	Gorgonian *Pseudopterogorgia* sp. South China Sea, in May 2015.	Inactive: cytotoxicity (A549 and A2780).	China
**212**	*Aspergillus versicolor*	Gorgonian *Pseudopterogorgia* sp. South China Sea, in May 2015.	Inactive: cytotoxicity (A549 and A2780).	China
**213**	*Aspergillus* sp.	Gorgonian *Melitodes squamata*, South China Sea, Sanya, Hainan Province, China (18°11′N, 109°25′E)	Active: antiviral (HSV-1).	China
**214**	*Aspergillus terreus*	Coral *Sarcophyton subviride*, coast of Xisha Island, South China Sea, in October 2016.	Active: moderate inhibitory activity against LPS-induced NO production.<br>Inactive: inhibitory effect (α-glucosidase).	China
**215**	*Aspergillus* sp.	Gorgonian *Melitodes squamata*, South China Sea, Sanya, Hainan Province, China (18°11′N, 109°25′E).	Inactive: cytotoxicity (HL60, HepG2 and MCF-7); antibacterial (*Bacillus subtilis* and *E. coli*).	China
**216**	*Aspergillus* sp.	Gorgonian *Melitodes squamata*, South China	Inactive: cytotoxicity (HL60, HepG2 and MCF-7); antibacterial	China

		Sea, Sanya, Hainan Province, China (18°11′N, 109°25′E).	(*Bacillus subtilis* and *E. coli*).	
**217**	*Aspergillus versicolor*	Coral, South China Sea.	Active: antibacterial (*Mycobacterium marinum*).	China
**218**	*Aspergillus versicolor*	Coral, South China Sea.	Active: antibacterial (*Mycobacterium marinum*).	China
**219**	*Aspergillus versicolor*	Coral, South China Sea.	Active: antibacterial (*Mycobacterium marinum*).	China
**220**	*Penicillium* sp.	Bivalve mollusk, *Meretrix lusoria*, Haikou Bay.	Active: inhibitory effect (protein tyrosine phosphatase 1B and TCPTP).	China
**221**	*Penicillium* sp.	Bivalve mollusk, *Meretrix lusoria*, Haikou Bay.	Active: inhibitory effect (protein tyrosine phosphatase 1B).	China
**222**	*Penicillium* sp.	Bivalve mollusk, *Meretrix lusoria*, Haikou Bay.	Active: inhibitory effect (protein tyrosine phosphatase 1B).	China
**223**	*Penicillium* sp.	Bivalve mollusk, *Meretrix lusoria*, Haikou Bay.	Active: inhibitory effect (protein tyrosine phosphatase 1B and PTPsigma).	China
**224**	*Penicillium* sp.	Bivalve mollusk, *Meretrix lusoria*, Haikou Bay.	Active: inhibitory effect (protein tyrosine phosphatase 1B).	China
**225**	*Penicillium* sp.	Bivalve mollusk, *Meretrix lusoria*, Haikou Bay.	Active: inhibitory effect (protein tyrosine phosphatase 1B).	China
**226**	*Penicillium* sp.	Bivalve mollusk, *Meretrix lusoria*, Haikou Bay.	Active: inhibitory effect (protein tyrosine phosphatase 1B and TCPTP).	China
**227**	*Penicillium* sp.	Bivalve mollusk, *Meretrix lusoria*, Haikou Bay.	Active: inhibitory effect (protein tyrosine phosphatase 1B and TCPTP).	China
**228**	*Penicillium* sp.	Bivalve mollusk, *Meretrix lusoria*, Haikou Bay.	Active: inhibitory effect (protein tyrosine phosphatase 1B).	China

**229**	*Aspergillus* sp.	Bivalve mollusk, *Sanguinolaria chinensis*, Haikou Bay.	Inactive: cytotoxicity (SGC-7901, K562, HeLa, and A549); antibacterial (*Staphylococcus aureus*, *Escherichia coli*, *Bacillus subtilis*, and *Streptococcus agalactiae*); quorum sensing (QS) inhibitory activity against Chromobacterium violaceum CV026.	China
**230**	*Aspergillus* sp.	Bivalve mollusk, *Sanguinolaria chinensis*, Haikou Bay.	Inactive: cytotoxicity (SGC-7901, K562, HeLa, and A549); antibacterial (*Staphylococcus aureus*, *Escherichia coli*, *Bacillus subtilis*, and *Streptococcus agalactiae*); quorum sensing (QS) inhibitory activity against Chromobacterium violaceum CV026.	China
**231**	*Aspergillus* sp.	Bivalve mollusk, *Sanguinolaria chinensis*, Haikou Bay.	Inactive: cytotoxicity (SGC-7901, K562, HeLa, and A549); antibacterial (*Staphylococcus aureus*, *Escherichia coli*, *Bacillus subtilis*, and *Streptococcus agalactiae*); quorum sensing (QS) inhibitory activity against Chromobacterium violaceum CV026.	China
**232**	*Aspergillus* sp.	Bivalve mollusk, *Sanguinolaria chinensis*, Haikou Bay.	Inactive: cytotoxicity (SGC-7901, K562, HeLa, and A549); antibacterial (*Staphylococcus aureus*, *Escherichia coli*, *Bacillus subtilis*, and *Streptococcus agalactiae*); quorum sensing (QS) inhibitory activity against Chromobacterium violaceum CV026.	China
**233**	*Aspergillus* sp.	Bivalve mollusk, *Sanguinolaria chinensis*, Haikou Bay.	Inactive: cytotoxicity (SGC-7901, K562, HeLa, and A549); antibacterial (*Staphylococcus aureus*, *Escherichia coli*, *Bacillus subtilis*, and *Streptococcus agalactiae*); quorum sensing (QS) inhibitory activity against Chromobacterium violaceum CV026.	China
**234**	*Aspergillus* sp.	Bivalve mollusk, *Sanguinolaria chinensis*,	Active: quorum sensing inhibitory activity against	China

		Haikou Bay.	*Chromobacterium violaceum* CV026.	
**235**	*Aspergillus* sp.	Bivalve mollusk, *Sanguinolaria chinensis*, Haikou Bay.	Active: quorum sensing inhibitory activity against *Chromobacterium violaceum* CV026.	China
**236**	*Aspergillus* sp.	Bivalve mollusk, *Sanguinolaria chinensis*, Haikou Bay.	Active: showed some weak activation effects on sfRyR. Inactive: inhibitory effect (α-glucosidase).	China
**237**	*Aspergillus* sp.	Bivalve mollusk, *Sanguinolaria chinensis*, Haikou Bay.	Active: showed some weak activation effects on sfRyR. Inactive: inhibitory effect (α-glucosidase).	China
**238**	*Aspergillus* sp.	Bivalve mollusk, *Sanguinolaria chinensis*, Haikou Bay.	Active: showed some weak activation effects on sfRyR. Inactive: inhibitory effect (α-glucosidase).	China
**239**	*Penicillium oxalicum*	Leaves of marine mangrove plant *Rhizophora stylosa*, Hainan Island, China.	Active: potent brine shrimp lethality.	China
**240**	*Penicillium brocae*	Marine mangrove plant *Avicennia marina*, Hainan Island, P. R. China, in August 2012.	Active: antibacterial (*Escherichia coli*, *S. aureus*, and *Vibrio harveyi*).	China
**241**	*Penicillium brocae*	Marine mangrove plant *Avicennia marina*, Hainan Island, P. R. China, in August 2012.	Inactive: cytotoxicity (A2780 and A2780 CisR); Antimicrobial (*E. coli*, *S. aureus*, *A. hydrophilia*, *V. parahemolyticus*, *V. harveyi*, *E. tarda*, *V. alginolyticus*, and *V. anguillarum*).	China
**242**	*M. irregularis*	Stems of the mangrove plant *Rhizophora stylosa* , Hainan Island, China.	Active: cytotoxicity (A-549 and HL-60).	China
**243**	*M. irregularis*	Stems of the mangrove plant *Rhizophora stylosa* , Hainan Island, China.	Active: cytotoxicity (A-549 and HL-60).	China
**244**	*M. irregularis*	Stems of the mangrove plant *Rhizophora stylosa* , Hainan Island, China.	Inactive: antitumor (A-549 and HL-60).	China
**245**	*M. irregularis*	Stems of the mangrove plant *Rhizophora stylosa* , Hainan Island, China.	Active: cytotoxicity (A-549 and HL-60).	China
**246**	*M. irregularis*	Stems of the mangrove plant *Rhizophora stylosa* , Hainan Island, China.	Inactive: antitumor (A-549 and HL-60).	China

**247**	*Penicillium chrysogenum*	mangrove , vein of *Myoporum bontioides* A. Gray , Leizhou Peninsula, Guangdong Province, China.	Active: cytotoxicity (MDA-MB-435 and SGC-7901).	China
**248**	*Penicillium chrysogenum*	Mangrove , vein of *Myoporum bontioides* A. Gray , Leizhou Peninsula, Guangdong Province, China.	Active: antifungal (*C. gloeosporioides*).	China
**249**	*Penicillium chrysogenum*	Mangrove, vein of *Myoporum bontioides* A. Gray, Leizhou Peninsula, China, in May 2014.	Active: antimicrobial (*Colletotrichum gloeosporioides* and *Rhizoctonia solani*); cytotoxicity (MDA-MB-435, SGC-7901 and A549).	China
**250**	*Neosartorya udagawae*	Root of the mangrove plant *Aricennia marina.*	Active: anti-influenza virus A (H1N1).	China
**251**	*Neosartorya udagawae*	Root of the mangrove plant *Aricennia marina.*	Active: anti-influenza virus A (H1N1).	China
**252**	*Neosartorya udagawae*	Root of the mangrove plant *Aricennia marina.*	Inactive: cytotoxicity (HL-60); anti-influenza virus A (H1N1).	China
**253**	*Neosartorya udagawae*	Root of the mangrove plant *Aricennia marina.*	Inactive: cytotoxicity (HL-60); anti-influenza virus A (H1N1).	China
**254**	*Eupenicillium* sp.	Mangrove *Xylocarpus granatum Koenig*, South China Sea in August 2015.	Active: cytotoxicity (A549 and HepG2).	China
**255**	*Eupenicillium* sp.	Mangrove *Xylocarpus granatum Koenig*, South China Sea in August 2015.	Active: cytotoxicity (A549, HeLa, and HepG2).	China
**256**	*Eupenicillium* sp.	Mangrove *Xylocarpus granatum Koenig*, South China Sea in August 2015.	Inactive: cytotoxicity (A549, HeLa, and HepG2); antibacterial (*Escherichia coli*, *Staphylococcus aureus*, methicillin-resistant *Staphylococcus aureus* MRSA, *Bacillus cereus*, *Vibrio parahemolyticus* and *V. alginolyticus*).	China
**257**	*Eurotium chevalieri*	Mangrove, *Rhizophora mucronata* Poir.,	Active: antimicrobial (inhibit the production of biofilm in *S.*	Portugal

		Chanthaburi Province, Eastern Thailand, in July 2010 (altitude 12°311′7.92″N, 101°54′ 01.06″E).	*aureus*).	
**258**	*Eurotium chevalieri*	Mangrove, *Rhizophora mucronata* Poir., Chanthaburi Province, Eastern Thailand, in July 2010 (altitude 12°311′7.92″N, 101°54′ 01.06″E).	Active: antimicrobial (inhibit the production of biofilm in *S. aureus*).	Portugal
**259**	*Eurotium cristatum*	Alga *Sargassum thunbergii*, Qingdao, China, in November 2009.	Inactive: antimicrobial (*Escherichia coli, Staphyloccocus aureus, Bacillus subtilis, Micrococcus luteus, Salmonella enteric, Bacillus pumilus, Alternaria brassicae, Valsa mali, Physalospora obtuse, Alternaria solania, Sclerotinia miyabeana, Magnaporthe grisea, Fusarium oxysporium, Botryosphaeria dothidea,* and *Colletotrichum gloeosporioides*).	China
**260**	*Eurotium cristatum*	Alga *Sargassum thunbergii*, Qingdao, China, in November 2009.	Active: exhibited potent lethal activity against brine shrimp and weak nematicidal effect against *Panagrellus redivivus.*<br>Inactive: antioxidative activities against DPPH and superoxide anion radicals.	China
**261**	*Eurotium cristatum*	Alga *Sargassum thunbergii*, Qingdao, China, in November 2009.	Active: exhibited weaklethal activities in the brine shrimp assay.<br>Inactive: antimicrobial (*Escherichia coli, Staphyloccocus aureus, Bacillus subtilis, Micrococcus luteus, Salmonella enteric, Bacillus pumilus, Alternaria brassicae, Valsa mali, Physalospora obtuse, Alternaria solania, Sclerotinia miyabeana, Magnaporthe grisea, Fusarium oxysporium, Botryosphaeria dothidea,* and *Colletotrichum gloeosporioides*).	China
**262**	*Eurotium cristatum*	Alga *Sargassum thunbergii*, Qingdao, China,	Active: exhibited weaklethal activities in the brine shrimp	China

		in November 2009.	assay.<br>Inactive: antimicrobial (*Escherichia coli, Staphyloccocus aureus, Bacillus subtilis, Micrococcus luteus, Salmonella enteric, Bacillus pumilus, Alternaria brassicae, Valsa mali, Physalospora obtuse, Alternaria solania, Sclerotinia miyabeana, Magnaporthe grisea, Fusarium oxysporium, Botryosphaeria dothidea,* and *Colletotrichum gloeosporioides*).	
**263**	*Paecilomyces variotii*	Red alga *Grateloupia turuturu*, red alga coast of Qingdao, China.	Active: cytotoxicity (A549, HCT116, and HepG2).	China
**264**	*Aspergillus alabamensis*	Red alga *Ceramium japonicum*, Qingdao, China.	Active: antimicrobial (*E. coli*, *M. luteus*, *Ed. ictaluri* and *V. alginolyticus*).	China
**265**	*Aspergillus alabamensis*	Red alga *Ceramium japonicum*, Qingdao, China.	Active: antimicrobial (*E. coli*, *M. luteus*, *Ed. ictaluri* and *V. alginolyticus*).	China
**266**	*Acrostalagmus luteoalbus*	Green alga *Codium fragile*, Sinop, Turkey.	Active: anti-acetylcholinesterase (AChE).<br>Inactive: antimicrobial (*Escherichia coli, Aeromonas hydrophila, Micrococcus luteus, Pseudomonas aeruginosa, Edwardsiella tarda, E. ictaluri, Vibrio alginolyticus, V. harveyi, V. parahemolyticus, V. anguillarum, V. vulnificus,Alternaria solani, Bipolaris sorokiniana, Ceratobasidium cornigerum, Colletotrichum gloeosporioides, C. glecosporioides Penz, Coniothyrium diplodiella, Fusarium graminerum, F. oxysporum, F. oxysporum.sp.cucumebrium Owen, F. oxysporum f. sp. momordicae nov. f., F. solani, Helminthosporium maydis, Penicillium, digitatum, Physalospora piricola, and Valsa mali*).	China
**267**	*Acrostalagmus luteoalbus*	Green alga *Codium fragile*, Sinop, Turkey.	Active: anti-acetylcholinesterase (AChE).<br>Inactive: antimicrobial (*Escherichia coli, Aeromonas hydrophila,*	China

			Micrococcus luteus, Pseudomonas aeruginosa, Edwardsiella tarda, E. ictaluri, Vibrio alginolyticus, V. harveyi, V. parahemolyticus, V. anguillarum, V. vulnificus,Alternaria solani, Bipolaris sorokiniana, Ceratobasidium cornigerum, Colletotrichum gloeosporioides, C. glecosporioides Penz, Coniothyrium diplodiella, Fusarium graminerum, F. oxysporum, F. oxysporum.sp.cucumebrium Owen, F. oxysporum f. sp. momordicae nov. f., F. solani, Helminthosporium maydis, Penicillium, digitatum, Physalospora piricola, and Valsa mali).	
**268**	*Acrostalagmus luteoalbus*	Green alga *Codium fragile*, Sinop, Turkey.	Inactive: antimicrobial (*Escherichia coli, Aeromonas hydrophila, Micrococcus luteus, Pseudomonas aeruginosa, Edwardsiella tarda, E. ictaluri, Vibrio alginolyticus, V. harveyi, V. parahemolyticus, V. anguillarum, V. vulnificus,Alternaria solani, Bipolaris sorokiniana, Ceratobasidium cornigerum, Colletotrichum gloeosporioides, C. glecosporioides Penz, Coniothyrium diplodiella, Fusarium graminerum, F. oxysporum, F. oxysporum.sp.cucumebrium Owen, F. oxysporum f. sp. momordicae nov. f., F. solani, Helminthosporium maydis, Penicillium, digitatum, Physalospora piricola, and Valsa mali*).	China
**269**	*Acrostalagmus luteoalbus*	Green alga *Codium fragile*, Sinop, Turkey.	Active: anti-microbial (*Fusarium solani*).	China
**270**	*Acrostalagmus luteoalbus*	Green alga *Codium fragile*, Sinop, Turkey.	Inactive: antimicrobial (*Escherichia coli, Aeromonas hydrophila, Micrococcus luteus, Pseudomonas aeruginosa, Edwardsiella tarda, E. ictaluri, Vibrio alginolyticus, V. harveyi, V. parahemolyticus, V. anguillarum, V. vulnificus,Alternaria solani, Bipolaris sorokiniana, Ceratobasidium cornigerum, Colletotrichum gloeosporioides, C.*	China

			glecosporioides Penz, Coniothyrium diplodiella, Fusarium graminerum, F. oxysporum, F. oxysporum.sp.cucumebrium Owen, F. oxysporum f. sp. momordicae nov. f., F. solani, Helminthosporium maydis, Penicillium, digitatum, Physalospora piricola, and Valsa mali).	
**271**	*Acrostalagmus luteoalbus*	Green alga *Codium fragile*, Sinop, Turkey.	Inactive: antimicrobial (*Escherichia coli, Aeromonas hydrophila, Micrococcus luteus, Pseudomonas aeruginosa, Edwardsiella tarda, E. ictaluri, Vibrio alginolyticus, V. harveyi, V. parahemolyticus, V. anguillarum, V. vulnificus,Alternaria solani, Bipolaris sorokiniana, Ceratobasidium cornigerum, Colletotrichum gloeosporioides, C. glecosporioides Penz, Coniothyrium diplodiella, Fusarium graminerum, F. oxysporum, F. oxysporum.sp.cucumebrium Owen, F. oxysporum f. sp. momordicae nov. f., F. solani, Helminthosporium maydis, Penicillium, digitatum, Physalospora piricola, and Valsa mali*).	China
**272**	*Aspergillus versicolor*	Alga *Enteromorpha prolifera*, Shilaoren beach, Qingdao, China, in July 2012	Inactive: antibacterial (*B. subtilis, P. aeruginosa, C. perfringens, S. aureus*[a], *E. coli, S. aureus*[b], *C. albicans, C. glabrata*).	China
**273**	*Aspergillus versicolor*	Alga *Enteromorpha prolifera*, Shilaoren beach, Qingdao, China, in July 2012	Inactive: antibacterial (*B. subtilis, P. aeruginosa, C. perfringens, S. aureus*[a], *E. coli, S. aureus*[b], *C. albicans, C. glabrata*).	China
**274**	*Penicillium* sp.	Brown alga *Padina* sp. (Van Phong Bay, South China Sea, Vietnam).	Active: demonstrated an effect in increasing cell viability in both 6-OHDA and PQ induced neuronal cell damage models.	Russia
**275**	*Aspergillus flavus*	Sponge *Agelas aff. nemo echinata*, Xisha Islands, China.	Inactive: cytotoxicity (P388, BEL-7402, A-549, Hela and HL-60); inhibitory effect (H1N1 and HIV); antimicrobial (*Mycobacterium phlei*, *Staphylococcus aureus*, *Colibacillus sp.* and *Blas-tomyces albicans*).	China
**276**	*Aspergillus flavus*	Sponge *Agelas aff. nemo echinata*, Xisha	Inactive: cytotoxicity (P388, BEL-7402, A-549, Hela and HL-60);	China

		Islands, China.	inhibitory effect (H1N1 and HIV); antimicrobial (*Mycobacterium phlei*, *Staphylococcus aureus*, *Colibacillus sp.* and *Blas-tomyces albicans*).	
**277**	*Aspergillus flavus*	Sponge *Agelas aff. nemo echinata*, Xisha Islands, China.	Inactive: cytotoxicity (P388, BEL-7402, A-549, Hela and HL-60); inhibitory effect (H1N1 and HIV); antimicrobial (*Mycobacterium phlei*, *Staphylococcus aureus*, *Colibacillus sp.* and *Blas-tomyces albicans*).	China
**278**	*Dichotomomyces cejpeii*	Sponge *Callyspongia sp. cf. C. flammea*, Bare Island, Sydney, Australia.	Not detected.	Germany
**279**	*Neosartorya glabra*	Sponge *Mycale* sp., coral reef at Samaesarn Island Gulf of Thailand, Chonburi Province, in February 2015, -15 to -20 m(12°34′36.64″N 100°56′59.69″E).	Inactive: antibacteria (*Escherichia coli* and *Staphyllococus aureus*); antifungal (*Aspergillus fumigatus*, *Trichophyton rubrum* and *Candida albicans*).	Portugal
**280**	*Neosartorya glabra*	Sponge *Mycale* sp., coral reef at Samaesarn Island Gulf of Thailand, Chonburi Province, in February 2015, -15 to -20 m(12°34′36.64″N 100°56′59.69″E).	Inactive: antibacteria (*Escherichia coli* and *Staphyllococus aureus*); antifungal (*Aspergillus fumigatus*, *Trichophyton rubrum* and *Candida albicans*).	Portugal
**281**	*Aspergillus* sp.	Sponge, Cheju Island, Korea in February 2009.	Active: inhibitory effect (PTP1B).	Republic of Korea
**282**	*Aspergillus carneus*	Sponge *Agelas oroides*, Aliağa-İzmir coast of the Aegean Sea, Turkey in June 2015.	Acctive: cytotoxicity (L5178Y).	Germany
**283**	*Aspergillus candidus*	Sponge *Epipolasis* sp., coral reef at Similan Island National Park, Phang-Nga province, Southern Thailand, in April 2010,-15 to -20 m(8°39′09″N, 97°38′27″ E).	Inactive: antibacterial (ESBL *E. coli*, VRE *E. faecalis*, MRSA S. aureus), biofilm formation inhibition activity; cytotoxicity (T98G and HepG2)<br>Active: cytotoxicity (Hep G2, HT29, HCT116, A549, A375, MCF7, U251 and T98G).	Portugal

**284**	*Aspergillus violaceofuscus*	Sponge *Reniochalina* sp., Xisha Islands, South China Sea.	Active: anti-inflammatory activity against IL-10 expression of the LPS-induced THP-1 cells.	China
**285**	*Hymeniacidon perleve*	Sponge *H. perleve*, Bohai Sea, Lingshuiqiao in Dalian, China.	Not detected.	China
**286**	*Aspergillus terreus*	Sponge *Callyspongia* sp., Xuwen County, Guangdong Province, China, in August 2013.	Inactive: inhibitory effect (MptpB); cytotoxicity (U87); against glutamate-induced toxicity HT22 .	China
**287**	*Aspergillus terreus*	Sponge *Callyspongia* sp., Xuwen County, Guangdong Province, China, in August 2013.	Inactive: inhibitory effect (MptpB); cytotoxicity (U87); against glutamate-induced toxicity HT22 .	China
**288**	*Aspergillus* sp.	Sponge.	Active: cytotoxicity (K562, BEL-7042, SGC-7901, A549, and Hela).	China
**289**	*Aspergillus* sp.	Sponge.	Active: cytotoxicity (K562, BEL-7042, SGC-7901, A549, and Hela).	China
**290**	*Aspergillus* sp.	Sponge.	Active: cytotoxicity (K562, BEL-7042, SGC-7901, A549, and Hela).	China
**291**	*Aspergillus* sp.	Sponge.	Inactive: cytotoxicity (K562, BEL-7042, SGC-7901, A549, and Hela).	China
**292**	*Aspergillus* sp.	Sponge.	Inactive: cytotoxicity (K562, BEL-7042, SGC-7901, A549, and Hela).	China
**293**	*Aspergillus* sp.	Sponge.	Inactive: cytotoxicity (K562, BEL-7042, SGC-7901, A549, and Hela).	China
**294**	*Eurotium* sp.	Sponge *Ircinia variabilis.*	Active: affinity for the cannabinoid CB1 receptor at low micromolar concentrations.	Egypt
**295**	*Penicillium* sp.	Sea anemone *Haliplanella luciae*, Qingdao coastline.	Inactive: antimicrobial (*Edwardsiella tarda*, *Escherichia coli*, *Micrococcus luteus*, *Pseudomonas aeruginosa*, *Vibrio alginolyticus*,	China

			V . harveyi, *V . parahaemolyticus*, *Alternaria alternata*, *A. brassicae*, *Colletotrichum gloeosprioides*, *Fusarium graminearum*, *F . oxysporum*, *Gaeumannomyces graminis*, *Phytophthora nicotiana*, *Physalospora piricola*, and *Valsa mali*).	
**296**	*Penicillium* sp.	Sea anemone *Haliplanella luciae*, Qingdao coastline.	Active: antimicrobial (*Vibrio parahaemolyticus*).	China
**297**	*Penicillium* sp.	Sea anemone *Haliplanella luciae*, Qingdao coastline.	Inactive: antimicrobial (*Edwardsiella tarda*, *Escherichia coli*, *Micrococcus luteus*, *Pseudomonas aeruginosa*, *Vibrio alginolyticus*, *V . harveyi*, *V . parahaemolyticus*, and plant pathogenic fungi *Alternaria alternata*, *A. brassicae*, *Colletotrichum gloeosprioides*, *Fusarium graminearum*, *F . oxysporum*, *Gaeumannomyces graminis*, *Phytophthora nicotiana*, *Physalospora piricola*, and *Valsa mali*).	China
**298**	*Aspergillus* sp.	Colonial ascidian (Shikotan Island, Pacific Ocean)	Active: Cytotoxicity (22Rv1).	Russia
**299**	*Aspergillus* sp.	Colonial ascidian (Shikotan Island, Pacific Ocean)	Not detected.	Russia
**300**	*Aspergillus* sp.	Colonial ascidian (Shikotan Island, Pacific Ocean)	Inactive: cytotoxicity (PC-3, LNCaP and 22Rv1).	Russia
**301**	*Aspergillus* sp.	Colonial ascidian (Shikotan Island, Pacific Ocean)	Not detected.	Russia
**302**	*Penicillium* sp.	Deep-sea water -4500 m, Yap Trench in West Pacific Ocean (138̊0.74′E, 8 ̊0.36′N).	Active: cytotoxicity (A549 and HeLa).	China
**303**	*Aspergillus* sp.	Sponge Xuwen County, Guangdong Province, China.	Active: antagonistic activity (HL60 and LNCaP).	China
**304**	*Chaetomium globosum*	Gut of the marine fish *Epinephelus drummondhayi*, Yellow Sea, Yancheng City,	Active: antibacterial (*Xanthomonas oryzae pv. oryzae*, *Ralstonia solanacearum*, *Xanthomonas oryzae pv. oryzicola*, and *Pseudomonas*	China

		China.	*syringae pv. Lachrymans*); inhibit the growth of the rice-pathogenic bacteria *Xanthomonas oryzae pv. oryzae* both *in vitro* and *in vivo.*	
**305**	*Chaetomium globosum*	Gut of the marine fish *Epinephelus drummondhayi*, Yellow Sea, Yancheng City, China.	Inactive: antifungal (*S. sclerotiorum, Botrytis cinerea*, *Fusarium solani*, and *Rhizoctonia cerealis*); antibacterial (*Xanthomonas oryzae pv. oryzae, Ralstonia solanacearum*, *Xanthomonas oryzae pv. oryzicola*, and *Pseudomonas syringae pv. Lachrymans*).	China
**306**	*Chaetomium globosum*	Gut of the marine fish *Epinephelus drummondhayi*, Yellow Sea, Yancheng City, China.	Active: antibacterial (*Xanthomonas oryzae pv. oryzae*, *Ralstonia solanacearum*, *Xanthomonas oryzae pv. oryzicola*, and *Pseudomonas syringae pv. Lachrymans*).	China
**307**	*Chaetomium globosum*	Gut of the marine fish *Epinephelus drummondhayi*, Yellow Sea, Yancheng City, China.	Inactive: antifungal (*S. sclerotiorum, Botrytis cinerea*, *Fusarium solani*, and *Rhizoctonia cerealis*); antibacterial (*Xanthomonas oryzae pv. oryzae, Ralstonia solanacearum*, *Xanthomonas oryzae pv. oryzicola*, and *Pseudomonas syringae pv. Lachrymans*).	China
**308**	*Chaetomium globosum*	Gut of the marine fish *Epinephelus drummondhayi*, Yellow Sea, Yancheng City, China.	Active: antibacterial (*Xanthomonas oryzae pv. oryzae*, *Ralstonia solanacearum*, *Xanthomonas oryzae pv. oryzicola*, and *Pseudomonas syringae pv. Lachrymans*).	China
**309**	*Chaetomium globosum*	Gut of the marine fish *Epinephelus drummondhayi*, Yellow Sea, Yancheng City, China.	Active: antibacterial (*Xanthomonas oryzae pv. oryzae*, *Ralstonia solanacearum*, *Xanthomonas oryzae pv. oryzicola*, and *Pseudomonas syringae pv. Lachrymans*).	China
**310**	*Penicillium brasilianum*	Bohai Sea (Huanghua, Hebei Province, China.	Active: cytotoxicity (HL-60).	China
**311**	*Penicillium brasilianum*	Bohai Sea (Huanghua, Hebei Province, China.	Active: cytotoxicity (MCF-7).	China
**312**	*Penicillium brasilianum*	Bohai Sea (Huanghua, Hebei Province, China.	Active: cytotoxicity (MCF-7).	China

**313**	*Aspergillus austroafricanus*	Seawater -30, Indian Ocean (88˚59′51′′E, 2˚ 59′54′′S).	Inactive: pro-angiogenic and anti-inflammatory; cytotoxicity (HepG2).	China
**314**	*Aspergillus austroafricanus*	Seawater, -30m, Indian Ocean (88˚59′51′′E, 2˚ 59′54′′S).	Active: pro-angiogenic activity in a PTK787-induced vascular injury.	China
**315**	*Aspergillus* sp.	Marine isopod Ligia oceanica was collected in Zhoushan, Zhejiang province of China, in December 2011.	Inactive: cytotoxicity (PC3 and HCT116).	China
**316**	*Aspergillus* sp.	Marine shrimp, Dinghai in Zhoushan, Zhejiang Province of China in December 2013.	Active: cytotoxicity (PC3).	China
**317**	*Aspergillus* sp.	Marine shrimp, Dinghai in Zhoushan, Zhejiang Province of China in December 2013.	Active: cytotoxicity (PC3).	China
**318**	*Aspergillus* sp.	Marine shrimp, Dinghai in Zhoushan, Zhejiang Province of China in December 2013.	Active: cytotoxicity (PC3).	China
**319**	*Aspergillus* sp.	Unidentified marine organism, Ross Sea (S 76°06.256′ , E 169°12.675′).	Active: cytotoxicity (inhibitory effects in BV2 cells and the same situation on LPS-stimulated NO production in RAW 264.7 and BV2 cells).	Republic of Korea
**320**	*Aspergillus* sp.	Unidentified marine organism, Ross Sea (S 76°06.256′ , E 169°12.675′).	Active: cytotoxicity (decreased PGE2 production in RAW 264.7 and BV2 cells).	Republic of Korea
**321**	*Aspergillus* sp.	Annelid *Osedax* sp., bone-eating worm, São Paulo Ridge, Brazil, -4203 m, 28°31.1′S and 41°39.4′W.	Active: antimicrobial (*S. parasitica*).	Japan

Table S3. Summary of the marine indole alkaloids isolated from marine invertebrates and plants.

No.	Source	Bioactivities	Country
**322**	Sponge *Hyrtios sp.*, Unten Port, Okinawa.	Active: antifungal (*A. niger*).	Japan
**323**	Sponge *Hyrtios sp.*, Unten Port, Okinawa.	Active: antibacterial (*E. coli*, *B. subtilis*).	Japan
**324**	Sponge *Hyrtios sp.*, Ishigaki island, Okinawa, Japan	Active: cytotoxicity (L1210).	Japan
**325**	Sponge *Hyrtios sp.*, Egyptian, coasts of the Red Sea, Sharm el-Sheikh, -30 ft.	Not detected.	Egypt
**326**	Sponge Hyrtios sp., Egyptian, coasts of the Red Sea, Sharm el-Sheikh, -9.14 m.	Active: antitrypanosomal.	Japan
**327**	Sponge *Hyrtios sp*, Weno island, Chuuk Atoll Federated States, Micronesia, -10 to -20 m.	Active: inhibitory effect (isocitrate lyase from Candida albicans).	Republic of Korea
**328**	Sponge *Hyrtios erectus*, Egypt.	Active: antiproliferative(HCT-116, MCF-7, HepG2); antibacterial activity(*S. aureus*, *E. coli)*.	Saudi Arabia
**329**	Sponge *Fascaplysinopsis reticulata*, Xisha Island of South China Sea..	Inactive: cytotoxicity (A549, HeLa, K562, Jurkat); inhibitory effect (tyrosine phosphatase 1B).	China
**330**	Sponge *Fascaplysinopsis reticulata*, Xisha Island of South China Sea.	Inactive: cytotoxicity (A549, HeLa, K562, Jurkat); inhibitory effect (tyrosine phosphatase 1B).	China
**331**	Sponge *Fascaplysinopsis reticulata*, Xisha Island of South China Sea.	Active: inhibitory effect (tyrosine phosphatase 1B).	China
**332**	Sponge *Fascaplysinopsis reticulata*, Xisha Island of South China Sea.	Active: inhibitory effect (tyrosine phosphatase 1B).	China
**333**	Sponge *Fascaplysinopsis reticulata*, Xisha Island of South China Sea.	Active: cytotoxicity (HeLa).	China
**334**	Sponge *Fascaplysinopsis reticulata*, Xisha Island of South China Sea.	Active: cytotoxicity (HeLa).	China

**335**	Sponge *Fascaplysinopsis reticulata*, Xisha Island of South China Sea.	Inactive: cytotoxicity (A549, HeLa, K562, Jurkat); inhibitory effect (tyrosine phosphatase 1B).	China
**336**	Sponge *Fascaplysinopsis reticulata*, Xisha Island of South China Sea.	Inactive: cytotoxicity (A549, HeLa, K562, Jurkat); inhibitory effect (tyrosine phosphatase 1B).	China
**337**	Sponge *Fascaplysinopsis reticulata*, Xisha Island of South China Sea.	Inactive: cytotoxicity (A549, HeLa, K562, Jurkat); inhibitory effect (tyrosine phosphatase 1B).	China
**338**	Sponge *Fascaplysinopsis reticulata*, Xisha Island of South China Sea.	Inactive: cytotoxicity (A549, HeLa, K562, Jurkat); inhibitory effect (tyrosine phosphatase 1B).	China
**339**	Sponge *Fascaplysinopsis reticulata*, Xisha Island of South China Sea.	Inactive: cytotoxicity (A549, HeLa, K562, Jurkat); inhibitory effect (tyrosine phosphatase 1B).	China
**340**	Sponge *Fascaplysinopsis reticulata*, Xisha Island of South China Sea.	Inactive: cytotoxicity (A549, HeLa, K562, Jurkat); inhibitory effect (tyrosine phosphatase 1B).	China
**341**	Sponge *Fascaplysinopsis reticulata*, Xisha Island of South China Sea.	Inactive: cytotoxicity (A549, HeLa, K562, Jurkat); inhibitory effect (tyrosine phosphatase 1B).	China
**342**	Sponge *Fascaplysinopsis reticulata*, Xisha Island of South China Sea	Inactive: cytotoxicity (A549, HeLa, K562, Jurkat); inhibitory effect (tyrosine phosphatase 1B).	China
**343**	Sponge *Fascaplysinopsis reticulata*, Passe Bateau, Mayotte (12˚58,653’S, 44 ˚58,949’E), -15 to –17 m.	Inactive: antibacterial (*Roseobacter litoralis*, *Shewanella putrefaciens*, *Vibrio carchariae*, *Vibrio natrigens*, *Vibrio proteolyticus*); antiplasmodial (*Plasmodium falciparum*).	France
**344**	Sponge *Fascaplysinopsis reticulata*, Passe Bateau, Mayotte (12˚58,653’S, 44 ˚58,949’E), -15 to –17 m.	Active: antimicrobial (*Vibrio natrigens*).	France
**345**	Sponge *Fascaplysinopsis reticulata*, Passe Bateau, Mayotte (12˚58,653’S, 44 ˚58,949’E), -15 to –17 m.	Active: antimicrobial (*Vibrio natrigens*).	France
**346**	Sponge *Acanthostrongylophora ingens*, -12 m, Sulawesi Island in Indonesia.	Active: cytotoxicity (MCF7, HCT116).	Saudi Arabia
**347**	Sponge *Acanthostrongylophora ingens*, -12 m, Sulawesi	Active: cytotoxicity (MCF7, HCT116).	Saudi Arabia

	Island in Indonesia.		
**348**	Sponge *Acanthostrongylophora ingens*, -12 m, Sulawesi Island in Indonesia in 2010	Active: cytotoxicity (MCF7, HCT116, A549).	Saudi Arabia
**349**	Sponge *Acanthostrongylophora ingens*, Sulawesi Island, -12 m, in 2010.	Active: cytotoxicity (MCF7, HCT116, A549).	Saudi Arabia
**350**	Sponge *Acanthostrongylophora sp.*, Kepulauan Seribu Marine National Park, north of Jakarta, -10 m, on February 19, 2012.	Active: cytotoxicity (K562, A549); antibacterial (*Staphylococcus aureus*, *Bacillus subtilis*, *Kocuria rhizophila*, *Salmonella enterica*, *Proteus hauseri*, *Escherichia coli*).	Korea
**351**	Sponge *Acanthostrongylophora sp.*, Kepulauan Seribu Marine National Park, north of Jakarta, -10 m, on February 19, 2012.	Active: cytotoxicity (K562, A549); antibacterial (*Bacillus subtilis*, *Kocuria rhizophila*, *Salmonella enterica*).	Korea
**352**	Sponge *Acanthostrongylophora sp.*, Kepulauan Seribu Marine National Park, north of Jakarta, -10 m, on February 19, 2012.	Active: cytotoxicity (K562, A549); antibacterial (*Staphylococcus aureus*, *Bacillus subtilis*, *Kocuria rhizophila*, *Salmonella enterica*, *Proteus hauseri*); inhibitory effect (Na+/K+-ATPase).	Korea
**353**	Sponge *Acanthostrongylophora sp.*, Kepulauan Seribu Marine National Park, north of Jakarta, -10 m, on February 19, 2012.	Active: cytotoxicity (K562, A549); antibacterial (*Staphylococcus aureus*, *Bacillus subtilis*, *Kocuria rhizophila*, *Salmonella enterica*, *Proteus hauseri*, *Escherichia coli*); inhibitory effect (Na+/K+-ATPase).	Korea
**354**	Sponge *Acanthostrongylophora sp.*, Kepulauan Seribu Marine National Park, north of Jakarta, -10 m, on February 19, 2012.	Active: cytotoxicity (K562, A549); antibacterial (*Staphylococcus aureus*, *Bacillus subtilis*, *Kocuria rhizophila*, *Salmonella enterica*, *Proteus hauseri*); inhibitory effect (Na+/K+-ATPase).	Korea
**355**	Sponge *Discodermia calyx*, an Escherichia coli clone incorporating metagenomic libraries.	Active: antimicrobial (*Bacillus cereus*, methicillin-sensitive *Staphylococcus aureus*, *Candida albicans*); cytotoxicity (P388).	Japan
**356**	Sponge *Discodermia calyx*, the metagenomic library of the marine sponge *Discodermia calyx*.	Inactive: antibacterial (*Bacillus cereus*).	China
**357**	Sponge *Spongia* sp., South Sea of Korea.	Inactive: antagonizing activitiy(FXR); cytotoxicity (CV-1).	Korea
**358**	Sponge *Spongia* sp., South Sea of Korea.	Inactive: antagonizing activitiy(FXR); cytotoxicity (CV-1).	Korea

**359**	Sponge *Haliclona* sp., SharmObhur, Jeddah, Saudi Arabian Red Sea coast.	Inactive: cytotoxicity (HepG-2, Daoy, HeLa).	Saudi Arabia
**360**	Sponge *Lipastrotethya* sp., Kurose (between 33°20.9082 N, 139°41.1862 E and 33°21.0722 N, 139°40.5142 E), north of Hachijo Island, -185 to -213 m.	Active: cytotoxicity (HeLa).	Japan
**361**	Sponge *Lipastrotethya* sp., Kurose (between 33°20.9082 N, 139°41.1862 E and 33°21.0722 N, 139°40.5142 E), north of Hachijo Island, -185 to -213 m.	Active: cytotoxicity (HeLa).	Japan
**362**	Sponge *Jaspis splendens*, Mid Reef (S 14.44813, E 144.88139), Great Barrier Reef, North Queensland, Australia on July 3, 2003, −23 m.	Active: multidimensional phenotype assay using nontransformed and nonimmortalized hONS cells.	Australia
**363**	Sponge *Jaspis splendens*, Mid Reef (S 14.44813, E 144.88139), Great Barrier Reef, North Queensland, Australia on July 3, 2003, −23 m.	Active: multidimensional phenotype assay using nontransformed and nonimmortalized hONS cells.	Australia
**364**	Sponge *Jaspis splendens,* three neighboring islands of East Kalimantan (Indonesia), namely Samama, Panjang and Shoal Islands, -10 m.	Active: cytotoxicity (L5178Y).	Egypt
**365**	Sponge *Jaspis splendens,* three neighboring islands of East Kalimantan (Indonesia), namely Samama, Panjang and Shoal Islands, -10 m.	Active: cytotoxicity (L5178Y).	Egypt
**366**	Sponge *Plakina jamaicensis*, Plana Cays (−31.4 m, 22°36.262′N, 73° 33.360′ W) ,Bahamas.	Not detected.	United States
**367**	Sponge *Geodia barretti*, Norwegian coast.	Inactive: inhibitory effect (electric eel AchE);<br>Active: inhibitory effect (BchE).	Norway
**368**	Sponge *Geodia barretti,* west of Iceland (65°27.6′ N–30°46.6′ W) in September 2010, -388 m.	Inactive: anti-inflammatory (inhibit DC secretion of IL-12p40 or IL-10).	Iceland

**369**	Sponge *Geodia barretti,* west of Iceland (65°27.6′ N–30°46.6′ W) in September 2010, -388 m.	Active: anti-inflammatory (inhibit DC secretion of IL-12p40, decrease T cell secretion of IFN-γ).	Iceland
**370**	Sponge *Geodia barretti,* west of Iceland (65°27.6′ N–30°46.6′ W) in September 2010, -388 m.	Active: anti-inflammatory (inhibit DC secretion of IL-12p40, decrease T cell secretion of IFN-γ).	Iceland
**371**	Sponge *Topsentia* sp., Palau, -140 m.	Active: antibacterial (*S. aureus*); anti-HIV activity (CCR5-tropic primary isolate YU2, CXCR4-tropic strain HxB2);<br>Inactive: cytotoxicity (BSC-1, HCT-116).	United States
**372**	Sponge *Topsentia* sp., Palau, -140 m.	Active: antibacterial (*S. aureus*); anti-HIV activity (CCR5-tropic primary isolate YU2, CXCR4-tropic strain HxB2);<br>Inactive: cytotoxicity (BSC-1, HCT-116).	United States
**373**	Sponge *Haplosclerida* , coast of Sulawesi, Indonesia, -10 m.	Active: cytotoxicity (PANC-1).	United States
**374**	Sponge *Dragmacidon* sp., coast of Tanzania ,-80 m.	Active: cytotoxicity (A549, HT29, MDA-MB-231); inhibitory effect (PP1 and/or PP2A phosphatases).	Spain
**375**	Sponge *Dragmacidon* sp., coast of Tanzania ,-80 m.	Active: cytotoxicity (A549, HT29, MDA-MB-231); inhibitory effect (PP1 and/or PP2A phosphatases).	Spain
**376**	Sponge *Trikentrion flabelliforme*, Exmouth Gulf (800 m south of Bundegi Beach), Western Australia, −9 m.	Not detected.	United States
**377**	Sponge *Trikentrion flabelliforme*, Exmouth Gulf (800 m south of Bundegi Beach), Western Australia, −9 m.	Not detected.	United States
**378**	Sponge *Trikentrion flabelliforme*, Exmouth Gulf (800 m south of Bundegi Beach), Western Australia, −9 m.	Not detected.	United States
**379**	Sponge *Trikentrion flabelliforme*, Exmouth Gulf (800 m south of Bundegi Beach), Western Australia, −9 m.	Not detected.	United States
**380**	Sponge *Trikentrion flabelliforme*, Exmouth Gulf (800 m south of Bundegi Beach), Western Australia, −9 m.	Not detected.	United States
**381**	Sponge *Trikentrion flabelliforme*, Exmouth Gulf (800 m	Not detected.	United States

	south of Bundegi Beach), Western Australia, −9 m.		
**382**	Sponge *Spongosorites calcicola*, Rathlin Island (Co. Antrim, Northern Ireland) ,-18 m.	Active: very weak or no cytotoxicity (Hela).	Ireland
**383**	Sponge *Spongosorites calcicola*, Rathlin Island (Co. Antrim, Northern Ireland) ,-18 m.	Active: very weak or no cytotoxicity (Hela).	Ireland
**384**	Sponge *Lamellomorpha strongylata*, the Western Continental Slope (Station J954), Northland, New Zealand, (34.6333S, 172.2250E) ,-200 m.	Inactive: antimicrobial (Methicillin-resistant *Staphylococcus aureus, Escherichia coli, Klebsiella pneumoniae, Acinetobacter baumannii, Pseudomonas aeruginosa, Candida albicans, Cryptococcus neoformans var. grubii*).	Australia
**385**	Sponge *Lamellomorpha strongylata*, the Western Continental Slope (Station J954), Northland, New Zealand, (34.6333S, 172.2250E) ,-200 m.	Active: antimicrobial (Methicillin-resistant *Staphylococcus aureus*).	Australia
**386**	Sponge *Lamellomorpha strongylata*, the Western Continental Slope (Station J954), Northland, New Zealand, (34.6333S, 172.2250E) ,-200 m.	Active: antimicrobial (Methicillin-resistant *Staphylococcus aureus*).	Australia
**387**	Sponge *Lamellomorpha strongylata*, the Western Continental Slope (Station J954), Northland, New Zealand, (34.6333S, 172.2250E) ,-200 m.	Inactive: antimicrobial (Methicillin-resistant *Staphylococcus aureus, Escherichia coli, Klebsiella pneumoniae, Acinetobacter baumannii, Pseudomonas aeruginosa, Candida albicans, Cryptococcus neoformans var. grubii*).	Australia
**388**	Sponge *Lamellomorpha strongylata*, the Western Continental Slope (Station J954), Northland, New Zealand, (34.6333S, 172.2250E) ,-200 m.	Inactive: antimicrobial (Methicillin-resistant *Staphylococcus aureus, Escherichia coli, Klebsiella pneumoniae, Acinetobacter baumannii, Pseudomonas aeruginosa, Candida albicans, Cryptococcus neoformans var. grubii*).	Australia
**389**	Sponge *Lamellomorpha strongylata*, the Western Continental Slope (Station J954), Northland, New Zealand, (34.6333S, 172.2250E) ,-200 m.	Active: antimicrobial (Methicillin-resistant *Staphylococcus aureus*).	Australia

**390**	Sponge *Guitarra fimbriata*, near Chirpoy Island, Pacific Ocean (46°14,8 N; 150°45,4 E, -250 m).	Inactive: inhibitory effect (alkaline phosphatase).	Russian Federation
**391**	Sponge *Guitarra fimbriata*, near Chirpoy Island, Pacific Ocean (46°14,8 N; 150°45,4 E, -250 m).	Inactive: inhibitory effect (alkaline phosphatase).	Russian Federation
**392**	Sponge *Guitarra fimbriata*, near Chirpoy Island, Pacific Ocean (46°14,8 N; 150°45,4 E, -250 m).	Active: inhibitory effect (alkaline phosphatase).	Russian Federation
**393**	Sponge *Guitarra fimbriata*, near Chirpoy Island, Pacific Ocean (46°14,8 N; 150°45,4 E, -250 m).	Active: inhibitory effect (alkaline phosphatase).	Russian Federation
**394**	Sponge *Guitarra fimbriata*, near Chirpoy Island, Pacific Ocean (46°14,8 N; 150°45,4 E, -250 m).	Inactive: inhibitory effect (alkaline phosphatase).	Russian Federation
**395**	Sponge *Guitarra fimbriata*, near Chirpoy Island, Pacific Ocean (46°14,8 N; 150°45,4 E, -250 m).	Inactive: inhibitory effect (alkaline phosphatase).	Russian Federation
**396**	Sponge *Callyspongia siphonella*, Hurghada along the Red Sea Coast (27°15048″ north (N), 33°4903″ east (E)) , -7 m.	Active: antibacterial (*Staphylococcus aureus* , *Bacillus subtilis*); biofilminhibitory (*Pseudomonas aeruginosa*); antitrypanosomal activity; cytotoxicity (HT29, OVCAR-3, MM.1S).	Egypt
**397**	Sponge *Callyspongia siphonella*, Hurghada along the Red Sea Coast (27°15048″ north (N), 33°4903″ east (E)) , -7 m.	Active: antibacterial (*Staphylococcus aureus* , *Bacillus subtilis*); biofilminhibitory (*Pseudomonas aeruginosa*); antitrypanosomal activity; cytotoxicity (HT29, OVCAR-3, MM.1S).	Egypt
**398**	Sponge *Gelliodes* sp., Hon Thom, Phu Quoc, Vietnam.	Inactive: cytotoxicity (HeLa, MCF-7, A549).	Japan
**399**	Sponge *Myrmekioderma* sp., southern Japan.	Active: antibacterial (*Escherichia coli*, *Bacillus subtilis*).	Japan
**400**	Sponge *Echinodictyum sp.*, Great Australian Bight, -65 m.	Active: antibacterial (*Serratia marcescens*, *Micrococcus luteus*, *Staphylococcus aureus*).	Australia
**401**	Sponge *Echinodictyum sp.*, Great Australian Bight, -65 m.	Active: antibacterial (*Serratia marcescens*, *Micrococcus luteus*, *Staphylococcus aureus*).	Australia
**402**	Sponge *Echinodictyum sp.*, Great Australian Bight, -65 m.	Active: antibacterial (*Serratia marcescens*, *Micrococcus luteus*, *Staphylococcus aureus*).	Australia

**403**	Sponge *Echinodictyum sp.*, Great Australian Bight, -65 m.	Active: antibacterial (*Serratia marcescens*, *Micrococcus luteus*, *Staphylococcus aureus*).	Australia
**404**	Sponge *Psammoclemma sp.*, Canal Woodin, New Caledonia (latitude 2233 270′ S; longitude 16716 343′ E), -25 to -40 m.	Active: cytotoxicity (KB).	France
**405**	Sponge *Plakortis sp.*, Zampa, Okinawa, Japan, -30 m.	Inactive: cytotoxicity (P388, B16).	Indonesia
**406**	Sponge *Plakortis sp.*, Zampa, Okinawa, Japan, -30 m.	Active: cytotoxicity (P388, B16).	Indonesia
**407**	Sponge *Amphimedon sp.*, Zamami, Okinawa, on October 29, 1982.	Active: antibacterial (*Escherichia coli*, *Stapylococcus aureus*, *Micrococcus luteus*, *Aspergillus niger*, *Trichophyton mentagrophytes*, *Candida albicans*, *Cryptococcus neoformans*).	Japan
**408**	Sponge *Damiria sp.*, Phuket Island, Thailand, in April 2014.	Active: cytotoxicity (sufficiently active to all NCI-60 cancer cell lines, and most effective to MALME-3M, MDA-MB-468, SW620, HCC-2998, MOLT4, K-562).	USA
**409**	Sponge *Damiria sp.*, Phuket Island, Thailand, in April 2014.	No data was repoted for the cytotoxicity evaluated on NCI-60 cell line panel.	USA
**410**	Sponge *Strongylodesma tongaensis*, Vava’u Island group, Kingdom of Tonga.	Inactive: cytotoxicity (HL-60).	New Zealand
**411**	Sponge collected from a reef, the west coast of Viti Levu, Fiji Islands (−17.839 S, 177.199 E), in March 2009.	Not detected.	U.K.
**412**	Sponge collected from a reef, the west coast of Viti Levu, Fiji Islands (−17.839 S, 177.199 E), in March 2009.	Not detected.	U.K.
**413**	Sponge *Narrabeena nigra*, the Alofi Island coast (1420′30″ S, 17804′53″ W), in December 2016, -8 m.	Inactive: cytotoxicity (SH-SY5Y, microglia BV2).	Ireland
**414**	Sponge *Narrabeena nigra*, the Alofi Island coast (1420′30″ S, 17804′53″ W), in December 2016, -8 m.	Inactive: cytotoxicity (SH-SY5Y, microglia BV2).	Ireland
**415**	Sponge *Narrabeena nigra*, the Alofi Island coast (1420′30″	Inactive: cytotoxicity (SH-SY5Y, microglia BV2).	Ireland

	S, 178°04′53″ W), in December 2016, -8 m.		
**416**	Sponge *Narrabeena nigra,* the Alofi Island coast (14°20′30″ S, 178°04′53″ W), in December 2016, -8 m.	Inactive: cytotoxicity (SH-SY5Y, microglia BV2).	Ireland
**417**	Sponge *Narrabeena nigra,* the Alofi Island coast (14°20′30″ S, 178°04′53″ W), in December 2016, -8 m.	Inactive: cytotoxicity (SH-SY5Y, microglia BV2).	Ireland
**418**	Sponge *Geodia sp.* , the Great Australian Bight in May 1990.	Inactive: antibacterial (*E. coli*, *Bacillus subtilis*, *Candida albicans*); cytotoxicity (SW620, NCI-H460).	Australia
**419**	Sponge *Geodia sp.* , the Great Australian Bight in May 1990.	Inactive: antibacterial (*E. coli*, *Bacillus subtilis*, *Candida albicans*); cytotoxicity (SW620, NCI-H460).	Australia
**420**	Sponge *Geodia sp.* , the Great Australian Bight in May 1990.	Inactive: antibacterial (*E. coli*, *Bacillus subtilis*, *Candida albicans*); cytotoxicity (SW620, NCI-H460).	Australia
**421**	Sponge *Geodia sp.* , the Great Australian Bight in May 1990.	Inactive: antibacterial (*E. coli*, *Bacillus subtilis*, *Candida albicans*); cytotoxicity (SW620, NCI-H460).	Australia
**422**	Sponge *Geodia sp.* , the Great Australian Bight in May 1990.	Inactive: antibacterial (*E. coli*, *Bacillus subtilis*, *Candida albicans*); cytotoxicity (SW620, NCI-H460).	Australia
**423**	Sponge *Geodia sp.* , the Great Australian Bight in May 1990.	Inactive: antibacterial (*E. coli*, *Bacillus subtilis*, *Candida albicans*); cytotoxicity (SW620, NCI-H460).	Australia
**424**	Sponge *Inf latella coelosphaeroides*, aboard the R/V Nathaniel B. Palmer research vessel.	Active: inhibition of *P. falciparum* liver-stage infection and development.	Australia
**425**	Sponge *Petrosia sp.,* the shore of Youngdeok-Gun, East Sea, Republic of Korea, -10 to -20m.	Active: antagonist effects on hFXR;<br>Inactive: cytotoxicity (HepG2).	Republic of Korea
**426**	Sponge *Petrosia sp.,* the shore of Youngdeok-Gun, East Sea, Republic of Korea, -10 m to -20m.	Inactive: cytotoxicity (HepG2).	Republic of Korea
**427**	Sponge *Petrosia sp.,* the shore of Youngdeok-Gun, East Sea, Republic of Korea, -10 m to -20m.	Inactive: cytotoxicity (HepG2).	Republic of Korea
**428**	Sponge *Microscleroderma sp.,* -1000 ft, Norfolk Rise, the	Active: antifungal (*C. albicans*).	New

	coast of New Caledonia.		Caledonia
**429**	Sponge *Microscleroderma herdmani*, Mauritius (latitude 20° 17.60′S, longitude 057° 21.06′E), on November 13, 1999, -45 m.	Active: antimicrobial (*Candida albicans*, *Candida glabrata*, *Candida krusei*, *Cryptococcus neoformans*, *Aspergillus fumigatus*).	USA
**430**	Sponge *Hexadella sp.*, (-40m) , Agamemnon Channel, Jervis Inlet, B.C.	Inactive: cytotoxicity (L1210).	Russia
**431**	Cnidarian *Heteractis aurora*, Bali, Indonesia, -3 m to -15 m.	Not detected.	Saudia Arabia
**432**	Cnidarian *Heteractis aurora*, Bali, Indonesia, -3 m to -15 m.	Not detected.	Saudia Arabia
**433**	Cnidarian *Abietinaria abietina*, Matua Island (48°51.8 N; 154°16.1 E), -102 m.	Active: increase the basal NF-κB-dependent transcriptional activity in JB6 Cl 41 NF-κB cells.	Russia
**434**	Cnidarian *Abietinaria abietina*, Matua Island (48°51.8 N; 154°16.1 E), -102 m.	Active: increase the basal NF-κB- dependent transcriptional activity in JB6 Cl 41 NF-κB cells.	Russia
**435**	Cnidarian *Thuiaria breitfussi*, Bjørnøya, Svalbard (79.0293 N, 20.8574 E), -48 m.	Active: cytotoxicity (MCF-7).	Norway
**436**	Cnidarian *Thuiaria breitfussi*, Bjørnøya, Svalbard (79.0293 N, 20.8574 E), -48 m.	Active: show preferential binding to kinases and inhibition of cell viability at promising potency and selectivity.	Norway
**437**	Cnidarian *Thuiaria breitfussi*, Bjørnøya, Svalbard (79.0293 N, 20.8574 E), -48 m.	Active: show preferential binding to kinases and inhibition of cell viability at promising potency and selectivity.	Norway
**438**	Cnidarian *Thuiaria breitfussi*, Bjørnøya, Svalbard (79.0293 N, 20.8574 E), -48 m.	Active: cytotoxicity (MCF-7, HT-29, MOLT-4, MV-4−11, MRC-5, MT3, SK-BR-3, JIMT1, HS578T, RDES); inhibition (PIM1 and DRAK1 kinases).	Norway
**439**	Cnidarian *Thuiaria breitfussi*, Bjørnøya, Svalbard (79.0293 N, 20.8574 E), -48 m.	Not dectected.	Norway
**440**	Cnidarian *Thuiaria breitfussi*, Bjørnøya, Svalbard (79.0293 N, 20.8574 E), -48 m.	Not dectected.	Norway
**441**	Bryozoan *Securiflustra securifrons*, coast of Hjelmsøya,	Active: cytotoxicity (A2058, HT-29, MCF-7, MRC-5).	Norway

	Norway.		
**442**	Bryozoan *Securiflustra securifrons*, coast of Hjelmsøya, Norway.	Active: cytotoxicity (A2058, HT-29, MCF-7, MRC-5).	Norway
**443**	Bryozoan *Securiflustra securifrons*, coast of Hjelmsøya, Norway.	Inactive: cytotoxicity (A2058, HT-29, MCF-7, MRC-5).	Norway
**444**	Bryozoan *Terminoflustra membranaceatruncata*, Marine Biological Station (Saint Petersburg State University), the White Sea in August 2014.	Inactive: antimicrobial (*Escherichia coli*, *Micrococcus luteus* B-6003, *Enterococcus faecalis*, *Candida albicans* 820, *Saccharomyces cerevisiae* 889/463).	Russia
**445**	Bryozoan *Terminoflustra membranaceatruncata*, Marine Biological Station (Saint Petersburg State University), the White Sea in August 2014.	Inactive: antimicrobial (*Escherichia coli*, *Micrococcus luteus* B-6003, *Enterococcus faecalis*, *Candida albicans* 820, *Saccharomyces cerevisiae* 889/463).	Russia
**446**	Bryozoan *Amathia verticillata*, Cabo Frio (RJ) and Porto Belo (SC), Little Jim and Coon Island, Florida.	Not detected.	Brazil
**447**	Bryozoan *A. lamourouxi*, rock pools of Woolgoolga, New South Wales, Australia.	Inactive: cytotoxicity (HEK293); antiplasmodial (chloroquine-sensitive 3D7 *P. falciparum* strain, chloroquine-resistant Dd2 *P. falciparum* strain).	Australia
**448**	Tunicate *Didemnum* sp.<br>collected by SCUBA near Haegeumgang, Geoje in the South Sea of Korea.	Inactive: antibacterial; antagonizing (farnesoid-X-receptor).	Republic of Korea
**449**	Tunicate *Cnemidocarpa stolonifera*, Peel Island, Myora Light, North Stradbroke Island, Queensland, Australia in 2005, -15 m.	Active: increased cell size, induced mitochondrial texture elongation and caused apoptosis in PC3;<br>Inactive: cytotoxicity (PC3).	Australia
**450**	Tunicate *Cnemidocarpa stolonifera*, Peel Island, Myora Light, North Stradbroke Island, Queensland, Australia in 2005, -15 m.	Active: increased cell size, induced mitochondrial texture elongation and caused apoptosis in PC3;<br>Inactive: cytotoxicity (PC3).	Australia
**451**	Pink mottled tunicate, the west coast of Viti Levu, Fiji	Not detected.	U.K.

	Islands (−17.839 S, 177.199 E), in March 2009.		
**452**	Pink mottled tunicate, the west coast of Viti Levu, Fiji Islands (−17.839 S, 177.199 E), in March 2009.	Not detected.	U.K.
**453**	Mollusk *Codakia orbicularis*, Thalassia testudinum seagrass sediments , Guadeloupe (lat 169′ 0.596″ N, long 6133′41.797″ W).	Inactive: antibacterial (*Enterococcus faecalis*, *Streptococcus pneumonia*, *Klebsiella pneumonia*, *Escherichia coli*, *Pseudomonas aeruginosa*); cytotoxicity (HCT116, U87-MG); kinases inhibition (Hs_CDK2/CyclinA, Hs_CDK5/p25, Hs_CDK9/CyclinT,Hs_RIPK3, Hs_Haspin, Hs_AuroraB, Ld_TLK, Hs-Pim1, Ssc_GSK3 a/b, Lm_CK1, and Rn_Dyrk1A).	France
**454**	Soft coral *Cespitularia taeniata*, Green Island, Taiwan, in March 2004.	Active: cytotoxicity (MCF-7, Daoy, Hela).	Taiwan, China
**455**	Cyanobacteria *Symploca* sp., Minna Island (‘‘croissant island”, Okinawa).	Active: inhibitory effect (NO production in LPS-stimulated RAW 264.3 cells).	Japan
**456**	Cyanobacteria *Symploca hydnoides*, shores of Trikora beach, Bintan Island in April 2018 and 2019.	Active: cytotoxicity (MOLT-4, AML2).	Singapore
**457**	Red algae *Rhodophyllis membranacea*, Moa Point, Wellington, New Zealand, -3 m to −10 m.	Active: cytotoxicity (HL-60);<br>Inactive: antifungal (wild-type *Saccharomyces cerevisiae* (baker’s yeast)).	New Zealand
**458**	Red algae *Rhodophyllis membranacea*, Moa Point, Wellington, New Zealand, -3 m to −10 m.	Not detected.	New Zealand
**459**	Red algae *Rhodophyllis membranacea*, Moa Point, Wellington, New Zealand, -3 m to −10 m.	Active: cytotoxicity (HL-60);<br>Inactive: antifungal (wild-type *Saccharomyces cerevisiae*).	New Zealand
**460**	Red algae *Rhodophyllis membranacea*, Moa Point, Wellington, New Zealand, -3 m to −10 m.	Active: cytotoxicity (HL-60);<br>Inactive: antifungal (wild-type *Saccharomyces cerevisiae*).	New Zealand
**461**	Red algae *Rhodophyllis membranacea*, Moa Point, Wellington, New Zealand, -3 m to −10 m.	Active: cytotoxicity (HL-60);<br>Inactive: antifungal (wild-type *Saccharomyces cerevisiae*).	New Zealand

**462**	Red algae *Rhodophyllis membranacea*, Moa Point, Wellington, New Zealand, -3 m to −10 m.	Not detected.	New Zealand
**463**	Red algae *Rhodophyllis membranacea*, Moa Point, Wellington, New Zealand, -3 m to −10 m.	Not detected.	New Zealand
**464**	Red algae *Rhodophyllis membranacea*, Moa Point, Wellington, New Zealand, -3 m to −10 m.	Not detected.	New Zealand
**465**	Red algae *Rhodophyllis membranacea*, Moa Point, Wellington, New Zealand, -3 m to −10 m.	Not detected.	New Zealand
**466**	Red algae *Rhodophyllis membranacea*, Moa Point, Wellington, New Zealand, -3 m to −10 m.	Not detected.	New Zealand
**467**	Red algae *Rhodophyllis membranacea*, Moa Point, Wellington, New Zealand, -3 m to −10 m.	Not detected.	New Zealand
**468**	Red algae *Laurencia similis,* South China Sea.	Active: antibacterial (*Staphylococcus aureus*, *Bacillus subtilis*, *Bacillus thuringiensis*, *Pseudomonas lachrymans*, *Agrobacterium tumefaciens*, *Xanthomonas vesicatoria*, *Ralstonia solanacearum*).	China
**469**	Red algae *Laurencia similis,* South China Sea.	Active: antibacterial (*Staphylococcus aureus*, *Bacillus subtilis*, *Bacillus thuringiensis*, *Pseudomonas lachrymans*, *Agrobacterium tumefaciens*, *Ralstonia solanacearum*).	China
**470**	Red algae *Laurencia similis,* South China Sea.	Active: antibacterial (*Xanthomonas vesicatoria*).	China
**471**	Mangrove *Acanthus ilicifolius*, leaves and stems, Zhanjiang mangrove national nature reserve, Guangdong Province, China.	Inactive: cytotoxicity (MCF-7, HL 60); protective effects (H_2O_2−, $A\beta_{25-35}$− and OGD-induced impairment in SH-SY5Y).	China
**473**	Mangrove *Hypocrea virens,* true mangrove flora (R. apiculata) , Shatian country, Guangxi province, China, in 2007.	Not detected.	China
